# A quantum-inspired classifier for clonogenic assay evaluations

**DOI:** 10.1038/s41598-021-82085-8

**Published:** 2021-02-02

**Authors:** Giuseppe Sergioli, Carmelo Militello, Leonardo Rundo, Luigi Minafra, Filippo Torrisi, Giorgio Russo, Keng Loon Chow, Roberto Giuntini

**Affiliations:** 1 University of Cagliari, Cagliari, Italy; 2 Institute of Molecular Bioimaging and Physiology, Italian National Research Council, Cefalú, Palermo Italy; 3 Department of Radiology, University of Cambridge, Cambridge, UK; 4 Cancer Research UK Cambridge Centre, University of Cambridge, Cambridge, UK; 5 Department of Biomedical and Biotechnological Sciences, University of Catania, Catania, Italy; 6 Centro Linceo Interdisciplinare “Beniamino Segre”, Accademia dei Lincei, Rome, Italy

**Keywords:** Computer science, Cellular imaging, Quantum information

## Abstract

Recent advances in Quantum Machine Learning (QML) have provided benefits to several computational processes, drastically reducing the time complexity. Another approach of combining quantum information theory with machine learning—without involving quantum computers—is known as Quantum-inspired Machine Learning (QiML), which exploits the expressive power of the quantum language to increase the accuracy of the process (rather than reducing the time complexity). In this work, we propose a large-scale experiment based on the application of a binary classifier inspired by quantum information theory to the biomedical imaging context in clonogenic assay evaluation to identify the most discriminative feature, allowing us to enhance cell colony segmentation. This innovative approach offers a two-fold result: (1) among the extracted and analyzed image features, *homogeneity* is shown to be a relevant feature in detecting challenging cell colonies; and (2) the proposed quantum-inspired classifier is a novel and outstanding methodology, compared to conventional machine learning classifiers, for the evaluation of clonogenic assays.

## Introduction

The synergies between machine learning and quantum theory has received a massive increase in the last decades^1–4^. One reason is due to the need for dealing with the current exponential growth of data being captured and stored^5^. Standard procedures frequently exhibit relevant slowdown in performances once these procedures are used in the treatment of big data. The advantages of quantum computation over conventional computation are widely discussed including the drastic reduction in the time complexity of a large set of algorithms. Moreover, recent progress made in the direction of producing real quantum computers suggested the combination between machine learning and quantum computing as a natural connection. However, the discussion involving real quantum computers is not the only way to exploit the properties of quantum theory at the service of machine learning; recent works showed that quantum information can inspire new ways to design machine learning algorithms without requiring the use of quantum computers^6,7^. In other words, it is possible to develop classical algorithms that are inspired by quantum information. This formalism, known as Quantum-inspired Machine Learning (QiML)^8^, is motivated by the fact that the expressive power of the quantum language makes it possible to gain relevant benefits for computational processes. QiML effectively exploits properties of quantum information theory to increase the accuracy of the process, rather than reducing the time complexity, such as in the case of standard Quantum Machine Learning.

Recently, promising results from QiML have shown to efficiently solve different kinds of classification problems, i.e., the problem of assigning each object of a given dataset to a membership class^9^. In particular, the work^6^ proposed a QiML technique for binary classification inspired by the theory of quantum state discrimination^10^, whereby the idea was in that discrimination between quantum states produces a very efficient classification process. The authors compared the QiML algorithm—called the Helstrom Quantum Classifier (HQC)—with other commonly used classifiers, by applying these classifiers to several conventional machine learning repository datasets, and they had obtained results which showed an average supremacy of the HQC compared to the other classifiers. This innovative approach suggested applications of the HQC on real-world datasets. A first attempt of the application of QiML technique to biological datasets have also previously been introduced^11^.

In this work, we show how the application of quantum information theory to machine learning turns out to be particularly beneficial in the context of biomedical images. In particular, we show a large-scale application of the HQC to support the evaluation in clonogenic assays. A clonogenic assay is a quantification technique of the survival degree of in vitro cell cultures, which is based on the ability of a single cell to grow and form a colony. To quantify the number and size of cell colonies after irradiation or drug administration (e.g., cytotoxic agents)^12,13^, a measure to assess the anti-proliferative use of these treatments is required. After some preparatory phases (i.e., plating, incubation, cell treatment^14^) the standard procedure includes colony counting with a stereo-microscope^15,16^. Traditionally, clonogenic assay evaluation is performed by manually counting the colonies composed of at least 50 densely-packed cells. To estimate the effect of the treatment on cell survival, the Plating Efficiency (PE), which is the fraction of colonies obtained from untreated cells, and the Surviving Fraction (SF) of cells after any treatment, are measured^14^. From a biological point of view, this quantification—which aims at identifying and quantifying the colonies grown following a specific treatment (e.g., radiation or drug/substance administration)—still represents an open problem. In fact, there are critical issues that are not completely solved yet, such as: (1) the high variability in the scenario related to the specific cell line used, and (2) the subjectivity in human quantification procedures. Depending on the cell line analyzed, the generated colonies can have very different characteristics, such as size, shape and heterogeneity (i.e., some colonies are small with well-defined boundaries and high-contrast compared to the background, whereas others are large and evanescent). A further difficulty of the evaluation process involves colonies which grow considerably and tend to merge together. Along with these high variabilities, human subjectivity can also affect the manual procedure. These issues introduce compelling challenges in manual procedures used in colony detection and quantification. Biologists typically attempt to reduce this lack of reliability, by considering the average of several manual counts.

Considering these challenging scenarios, recent research efforts^17–19^ have proposed an alternative solution to common counting procedures. In particular, rather than quantifying the number of colonies, the area covered by cell colonies is determined. Experimental evidence showed that the area covered by a colony is correlated to the colony number and size. In fact, area-based approaches—which determines the area of the well plates covered by the colonies—represent a useful alternative, allowing us to provide a measure equivalent to the exact count of colonies. To quantify the number of colonies grown after a treatment, a post-processing step, which evaluates the number of colonies contained in the segmented regions, would be integrated into the processing pipeline in area-based approaches. This surrogate measure allows us to overcome some of the problems highlighted above, such as the difficulty of correctly quantifying the colonies which, due to the growth, have merged together.

In this work, an area-based approach is proposed, which is based on imaging characteristics that are not observable by the naked human eye. In particular, we start from the intrinsic assumption that biomedical images often convey information—contained in so-called descriptors (i.e., *contrast*, *correlation*, *energy* and *homogeneity*)—about the phenotype of the underlying physiopathology, which is not always easily identifiable by a simple visual inspection by the human eye. These descriptors can be revealed by quantitative analysis, by converting the images into a high-dimensional dataset, and making it possible to extract further information. In our biological setting, along with the native imaging characteristics—i.e., Red Green Blue (*RGB*) and International Commission on Illumination (CIE) L*u*v* pixel values—these descriptors are used in the classification of colonies vs. background area, where these high-dimensional set of descriptor features makes it possible to enhance the detection of difficult cell lines.

Summarizing, the area-based approach strictly depends on the colonies vs. background binary classification, where the descriptors assume the role of the features. Several algorithms and techniques have already been explored in the classification of colonies vs. background area and, specifically, in the context of clonogenic assays^17,20–25^. We here introduce a multidisciplinary effort which involves image processing, machine learning, quantum information theory, and cell biology (see Fig. 2a). In particular, we apply the HQC to the binary classification of colonies vs. background area over four different cell lines. Each cell line is given by a dataset where each row in the dataset is a vector that the HQC has to classify as belonging to a colony area or to a background area by using the information provided by the corresponding features. Our experimental study is divided into two stages: (1) we analyze the relevance of different features (descriptors) during the classification process to identify the one that optimizes the accuracy in the colonies vs. background discrimination, and (2) we provide a full comparison between HQC and other conventional classifiers aiming to show that the HQC deserves to be considered as a performant classifier in the real context of clonogenic assay evaluations.

## Materials and methods

This section first describes the datasets analyzed in our experiments (i.e., the well plates with cell colonies) along with how the features—which are the inputs of the HQC—were extracted and prepared from the Grey Level Co-occurrence Matrix (GLCM) of the well plate images^26,27^. The section then outlines the setup of the HQC.

### Dataset description

**Figure 1:**
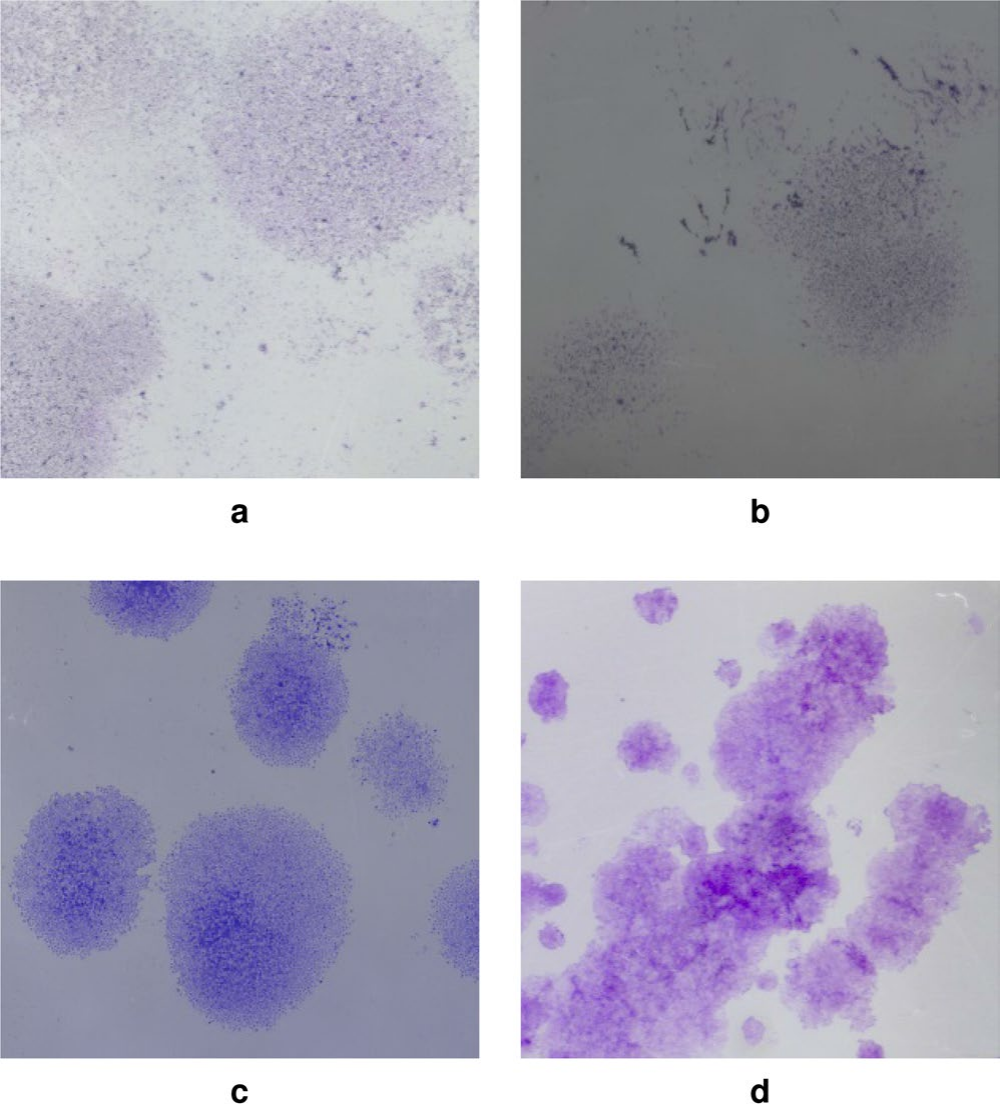
Examples of the wells analyzed in this study. Colony images are displayed for each cell line: (**a**) MDA-MD-231. (**b**) U87-MG. (**c**) U251. (**d**) MCF7. Images are depicted by a reduced 0.25 factor to the original acquisition size.

The imaging data used for clonogenic assay evaluation were images of 6-well plates (produced by Corning Inc., Corning, NY, USA) regarding four different cell lines: (1) MDA-MD-231 is a human metastatic breast cancer cell line which represents an *in vitro* model of a subgroup of breast cancer, particularly radioresistant and refractory to conventional therapies; (2) U87-MG is a human glioblastoma multiforme cell line; (3) MCF7 is a breast epithelial cell line which is often used in the field of cell biology; and (4) U251 is a human glioblastoma cell line used in brain cancer research and drug development. Figure 1 shows an example of each cell line analyzed in this study.

The images of the well plates were acquired using a common desktop flat-bed scanner, with a resolution of 800 dpi and a 24-bit color-depth. For each well plate image, only a squared area (about $300 \times 300$ pixels) composed of about $10^5$ pixels was considered to reduce the computational time. Thirty well plates for each cell line were considered, treated with different doses of particles (i.e., protons, photons) and/or cytotoxic agents (e.g., curcumin, SLNB).

Such cell lines considered in this work have different characteristics, with colonies having different size, shape, contrast, and uniformity. In these trials, we considered the most challenging scenarios where MDA-MD-231 and U87-MG are cell lines particularly difficult to quantify in clonogenic assays because it produces non-compact colonies, and can sometimes be evanescent because they tend to take up very few crystal-violet, a dye commonly added to the culture plate by biologists to increase the contrast of the colonies.

### Dataset preparation

The initial part of the experiment was devoted to the preparation of the datasets. In this experiment, we applied the HQC to the four considered cell lines. For each cell line, we considered 30 images, which were obtained from 30 different well plates. In order to quantify the effectiveness of the classifier and to determine the most discriminative feature, before applying the HQC, each image was segmented to define the ground-truth, which is then used to compare the classification result achieved by the HQC and the competing classifiers. These masks—validated by biologists—were the result of colonies-background segmentation by means of spatial Fuzzy C-Means (sFCM) clustering using the pixelwise entropy feature maps of the well plate. The value 1 (or 0) associated with each pixel within this mask represented the class membership (or not) of the pixel to a colony. Finally, the mask obtained by sFCM clustering underwent a post-processing step which removes small connected-components, to consider only the colonies comprising of at least 50 densely-packed cells^14^. The choice of entropy to determine the ground-truth was motivated by a previous work^19^, which showed a high correlation between area-based quantification by entropy and manual quantification.

A particular aim of the experiment is to compare different inputs to find out whether, in general, any feature outperforms the others in the classification process. In particular, the six investigated features in our experiment were: the *RGB* and L*u*v* (where L* represents the lightness, while u* and v* denote the chromaticity) color space encodings, as well as the *contrast*, *correlation*, *energy* and *homogeneity* descriptors. For this reason, the dataset was split into 6 different datasets (one for each feature) and properly formatted to obtain a file suitable for the HQC. In particular, the obtained segmentation mask (our ground truth) was ‘serialized’ forming a set where each row, which represents the characteristics of each pixel, is structured as follows: (1) the first two columns represent the two-dimensional coordinates of the pixel, (2) the last column denotes the class label of the pixel (1 if belongs to a colony, and 0 if the pixel belongs to a background), and (3) the middle columns store the values of the features for each pixel. Prior to classification, all the values of the features were normalized in the range [1, 255]. Hence, we performed the experiment over 4 cell lines, each one included 30 different well images that yielded 6 distinct datasets; therefore, the total number of datasets is 720.

### Extracted features

For each input image, along with the original encoding in the *RGB* and L*u*v* color spaces, the following feature maps were also extracted from the GLCM, namely: *contrast*, *correlation*, *energy*, and *homogeneity*.

More specifically: (1) *contrast* represents a measure of the intensity contrast between a pixel and its neighbor over the whole image, (2) *correlation* denotes a measure of how correlated a pixel is to its neighbor over the whole image, (3) *energy* (i.e., angular second moment) yields the sum of squared elements in the GLCM, and (4) *homogeneity* quantifies the closeness of the distribution of elements in the GLCM to the GLCM diagonal. The feature maps were computed using the MatLab (The Mathworks, Natick, MA, USA) built-in function graycoprops, which relies upon the graycomatrix function.

These GLCM-based local texture descriptors are comprised among the so-called Haralick’s features^26,27^. In particular, the input images were quantitized (i.e., histogram rebinning) by using *L* gray-levels and processed by a sliding squared window of size $\omega \times \omega$ pixels^28^. The parameters for the feature extraction were: sliding window size $\omega \times \omega = 5$ pixels, number of gray-level bins $L = 256$. For a detailed description of the mathematical formulation, please refer to Supplementary Material S1.

We compared the various features extracted individually, with the goal of understanding the most discriminative one. In summary, the HQC was tested on the following set of features: (1) *RGB* color space triplet, (2) L*u*v* color space triplet, (3) *contrast*, (4) *correlation*, (5) *energy*, and (6) *homogeneity*. The use of such a procedure, which separately analyzed the 6 image features, rather than a wrapper method for feature selection^29^ was mostly motivated by computational limitations^30^. In wrapper methods, the feature selection criterion is based on the performance of a subset of the predictors, by searching for the highest classification performance. Indeed, wrapper methods rely on the classification evaluation for obtaining the optimal feature subset: this search in the feature space is a non-deterministic polynomial-time hard (NP-hard) problem. Exhaustive search methods are computationally intensive and infeasible for large-scale datasets, thus search methods and metaheuristics are typically used to find sub-optimal solutions in the search space^31^. Importantly, overuse of the accuracy estimates in feature subset selection may cause overfitting in the feature subset space due to multiple comparisons and hinders generalization capabilities^32^. Therefore, in our experiments, we aimed at identifying the most discriminative feature in colony vs. background classification by fairly evaluating several different binary classifiers.

### Setup of the HQC

Following standard procedures, pre-processing was applied to the 720 datasets before training the HQC on these datasets. In particular, the pre-processing phase consisted of three steps: (1) random sampling, (2) standardization, and (3) splitting the sampled dataset into development and test sets ($80\%$ and $20\%$, respectively).

The random sampling simply consisted of the random extraction of a subset over each of the initial 720 datasets. Each of the initial dataset has cardinality $301^2$ (the number of the pixels) while the sampled dataset has cardinality 181, hence we considered a random sampling pre-processing step that randomly extracted a different $0.2\%$ sample from each of the 720 datasets. In particular, there were 30 datasets for each cell line and each feature, and a different $0.2\%$ random sample was extracted from each of the 30 datasets, to train the HQC. In the standardization step, the six features of the sampled dataset (*RGB*, L*u*v*, *contrast*, *correlation*, *energy* and *homogeneity*) were standardized to have mean equal to 0 and standard deviation equal to 1 by using the individual feature’s mean and standard deviation values (i.e., *z*-score standardization).

The HQC was trained on the training set and hypertuning is performed simultaneously using the classifier’s four hyperparameters: (1) the rescaling factor, (2) the encoding method, (3) the number of copies taken for the density matrices, and (4) the class weights assigned to the quantum centroids in the HQC.

**Figure 2:**
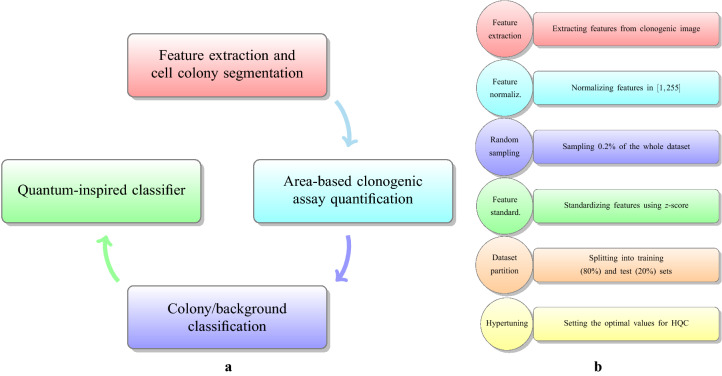
Conceptualization. The general scheme of the process: (**a**) conceptual scheme of the proposed multidisciplinary approach involving image processing, machine learning, quantum information theory and cell biology. (**b**) The pre-processing steps.

The first hyperparameter, the rescaling factor, involves the multiplication of the values of each feature with a scalar factor. As already shown^33^, even though this procedure is generally not beneficial for conventional classification approaches, a suitable choice for the rescaling factor can produce relevant advantages for the HQC in terms of the improvement to the classifier’s performance. We considered rescaling factors in the set $\{0.5,1,1.5,2\}$. The second hyperparameter was the encoding method that was adopted. In order to apply the HQC, we need to encode each row data ${\mathbf {X}}$ (a real vector whose elements are the the respective features) into a density matrix (also called *density pattern*), $\rho _X$, which is the standard mathematical object representing a quantum state. In our experiment we considered two different encoding methods: the *stereographic encoding* (SE) and the *amplitude encoding* (AE). Intuitively, the SE is inspired by geometrical considerations and associates each real vector ${\mathbf {X}}$ to a point of a hypersphere with unitary radius, which has a natural interpretation in the standard quantum scenario. On the other hand, the AE is based on the idea of keeping the information about the amplitude of the vector by considering this as a particular feature^34^. Both the SE and AE were previously detailed^35^. The third hyperparameter was given by taking a certain number of *copies* for each row vector ${\mathbf {X}}$ of the encoded training set (which has now been encoded into density matrices). Formally, taking a certain number of *copies* is provided by tensor products of the density patterns $\rho _X$ with itself (i.e., $\rho _X\otimes \rho _X\otimes \ldots \otimes \rho _X$), obtaining a new set of density patterns. The idea for this procedure originates from quantum information theory where—unlike in the classical case—taking copies of a given state $\rho$ provides additional information with respect to the initial state. In particular, considering more copies of the states can increase the probability of providing a correct discrimination between two quantum states^6^. Let us remark how this is relevant because it suggests that the performance of the HQC could be, in principle, improved by increasing the number of the copies for each density pattern obtained from the initial dataset. In the experiment, we considered a number of copies equal to $\{1,2,3,4\}$ for the image features *RGB* and L*u*v*; and $\{1,2,3,4,5\}$ for the image features *contrast*, *correlation*, *energy* and *homogeneity*. The last hyperparameter was represented by two types of class weights assigned to the two quantum centroids in the HQC. The first type, called equiprobable, assigns equal weights of 1/2 to both of the two quantum centroids; the second type, called weighted, assigns to each centroid a weight which is proportional to the cardinality of the respective classes^6^. The pre-processing and hypertuning steps are outlined in Fig. 2b. The performance metrics considered in the experiment were the balanced accuracy and Area Under the Receiver Operating Characteristic (AUROC) scores. The balanced accuracy score was chosen to ensure the evaluation of the classification task of a pixel as either a colony or a background are both equally relevant. The AUROC score was chosen to enable the evaluation of the overall performance of a classifier. To obtain the combination of hyperparameters which maximizes any of the two performance metrics, we first partitioned the development set into 5 subsets of the same cardinality. According to the most common experimental procedures, during the development phase, we performed a 5-fold cross-validation. The model performance for each combination of hyperparameters is obtained by averaging the validation set’s performance over the 5 rounds.

The same procedure was performed to determine the best combination of hyperparameters for the other 18 (generally, high performing and well-established) conventional machine learning classifiers. The 18 classifiers considered were: AdaBoost, Bernoulli Naïve Bayes, Dummy Classifier, Extra Trees, Gaussian Naïve Bayes, Gradient Boosting, Linear Discriminant Analysis, Logistic Regression, Multi Layer Perceptron, Nearest Centroid, Nearest Neighbors, Passive Aggressive Classifier, Perceptron, Quadratic Discriminant Analysis, Random Forest, SVM (with linear kernel), SVM (with polynomial kernel), and SVM (with RBF kernel). For the performance metric AUROC score, three classifiers—Nearest Centroid, Passive Aggressive Classifier and Perceptron—were excluded from this performance metric analysis due to the unavailability of the predicted class probabilities required in the AUROC score calculation.

The HQC was also compared with these 18 other classifiers (for the balance accuracy score) or 15 other classifiers (for the AUROC score) by determining the best image feature (among *RGB*, L*u*v*, *contrast*, *correlation*, *energy*, or *homogeneity*) and the best classifier—i.e., the HQC or the other 18 (or 15) classifiers—which yielded the highest performance on the test set, individually for each of the two performance metrics, balance accuracy and AUROC scores respectively.

## Experimental results

Experimental tests were carried out exhaustively, in order to quantify the effectiveness of the classifier and to establish the most discriminative feature (in terms of colonies vs. background). As a reference for comparing the HQC classification results against the other standard classifiers, we used the ground-truth masks previously calculated and validated by experimental biologists. These masks were the result of colonies-background segmentation by means of sFCM clustering using entropy as a discriminant^19^. The mask obtained by the sFCM clustering, prior to be used as ground-truth, underwent a post-processing step (i.e., morphological operations and small connected-component removal), aiming to cope with the noise in the well background and to consider only the colonies composed of, at least, 50 densely-packed cells^14^.

We first present the experimental results for the best performing image feature for each of the four cell lines, which can be found under section Supplementary Material S2. The results for each cell line consists of two parts. The first part shows: (1) heatmaps of the balanced accuracy scores over the 30 datasets for the HQC and the other 18 classifiers, obtained by hypertuning the hyperparameters of each classifier in order to optimize the balanced accuracy score; (2) heatmaps of a classifier outperforming (“wins”) over another classifier (“losses”) out of the 30 datasets; and (3) a table showing the averaged scores over the 30 datasets for each of the 6 image features and 18 classifiers. The second part for each cell line is analogous to the first, where the role of the balanced accuracy score is replaced by the AUROC score. The whole performance evaluation was executed using the test set. The aim of this experimental procedure was to find the most informative feature that classifies a pixel as either a colony or a background, i.e., the feature that maximizes the value of the balanced accuracy and the AUROC scores, respectively.

The experimental results shown in Supplementary Material S2 are summarized in Tables 1 and 2. These tables were obtained by extracting the best performing image feature and, for this image feature, the best classifier up to and including the HQC were presented. The corresponding Jaccard index and Dice coefficient values are also shown for each table.

A premise is needed. We observe the colony vs. background classification task on the datasets considered in this paper generally produces a high performance score. An explanation for this is because most of the pixels belonging to a given colony or background class are concentrated together in a large part in each of the images (see Fig. 1). Hence, the performance of most of the classifiers are generally good. For this reason, the performance for a number of classifiers will generally be quite high and the differences in the performances observed among these classifiers are very subtle. The ease of the classification task on these datasets further gives rise to the sufficient need for extracting only $0.2\%$ samples from each of the 720 datasets used in the training pipeline of the classifiers.

The results in Tables 1, 2 and 3 (which we will discuss in more detail below) clearly show that, on average, the best performing image feature and classifier combination is given by *homogeneity* and the HQC.

For cell line MDA-MD-231, Tables S2.1.1 and S2.1.2 (see Supplementary Material S2.1) show the best image feature for both the balanced accuracy and AUROC scores is *homogeneity*. For this image feature, we can observe that the HQC was the best performing classifier for the balanced accuracy score and it was also one of the best performing classifier for the AUROC score (see Tables 1, 2). In previous work^6^, we discussed the potential of the HQC achieving a higher performance is dependent upon the number of copies taken for the density patterns. In other words, increasing the number of copies increases, on average, the performance of the classifier. Consequently, the computation complexity during training increases (whereby the computational complexity is ${\mathcal {O}}(n^m)$, where *n* and *m* are the number of features and number of copies, respectively). In principle, a multiple-core computational platform or server would allow the HQC to achieve a higher performance which leads to potential future experiments to be explored assessing the real limits of the HQC with more powerful computing infrastructures. Inspired by this motivation, we repeated the experiment by increasing the number of copies by an additional copy for cases where the HQC is not, initially, the best performing classifier. As an example, in Table 3a we show how by adding one more copy, the AUROC score (averaged across 30 *homogeneity* feature datasets) increases from 0.954 to 0.969, making the HQC the best performing classifier for cell line MDA-MD-231.

For cell line U87-MG, Tables S2.2.1 and S2.2.2 (see Supplementary Material S2.2) and Tables 1 and 2 show a subtle but clear supremacy of the image feature and classifier combination of *homogeneity* and the HQC, for both balanced accuracy and AUROC scores. However, it is worth noting that the balanced accuracy and AUROC scores obtained for cell line U87-MG for all classifiers (including HQC) were lower compared to the other three cell lines, indicating the classification task of discriminating a pixel being a colony or a background class to be slightly more difficult for the cell line U87-MG in comparison to the other three cell lines.

Unlike U87-MG, the balanced accuracy and AUROC scores obtained for cell line MCF7 were high, thus indicating this cell line was particularly simple to classify and naturally resulting in the comparison among the best performing classifiers more unstable. For this cell line, Tables S2.3.1 and S2.3.2 (see Supplementary Material S2.3) show the best image feature is L*u*v*, and for this image feature, the best classifier was *Gaussian Naïve Bayes* for both the balanced accuracy and AUROC scores (see Tables 1, 2). Even though the balanced accuracy and AUROC scores obtained with the HQC were not considerably different from that of the *Gaussian Naïve Bayes*, we repeated the experiment by considering the HQC with one additional copy (using the similar procedure as described for cell line MDA-MD-231 above). In Tables 3b and 3c, we show the scores (averaged across 30 L*u*v* feature datasets) of the HQC obtained with the additional copy outperformed (for the balance accuracy score) and equalizes (for the AUROC score) the performance of the *Gaussian Naïve Bayes*.

Finally, for cell line U251, Tables S2.4.1 and S2.4.2 (see Supplementary Material S2.4) show the best image feature for both the balanced accuracy and AUROC scores is *homogeneity*. For this image feature, we can observe the HQC is close to (for the balance accuracy score) or is one of the best (for the AUROC score) performing classifier (see Tables 1, 2). We also note the balanced accuracy and AUROC scores obtained are high, indicating this cell line was also particularly simple to classify and naturally resulting in the comparison among the best performing classifiers more unstable. Again, we repeated the experiment for the balanced accuracy score by considering taking one more additional copy for the HQC and this gave us a further small increase of the performance of the HQC to equalize the performance of the SVM (with linear kernel) (see Table 3d).

Along the whole experiment, the calculation of the Jaccard index and the Dice coefficient confirms a good similarity of the sample sets. Moreover, in order to show how, for these clonogenic assay datasets, the $0.2\%$ random sample extraction of the datasets is sufficiently representative, a sub-experiment was performed where the trained HQC model was tested on a new unseen test set extracted from the remaining $99.8\%$ of the datasets. This experiment was done by randomly selecting 10 datasets (out of the 30 datasets) from the best performing image feature for each of the four cell lines. The results are shown in Tables S3.1–S3.4 (see Supplementary Material S3) where we have presented a comparison of the performance on this new unseen test set against the performance on the test set from the $0.2\%$ random sample used in the main experiment. We could see, on average over the 10 datasets, the performance on both of these test sets is generally not considerably different, suggesting that a small training set is sufficient for the HQC to perform well on classification tasks on these type of clonogenic assay datasets. In conclusion, our results showed how the HQC is particularly efficient for the colony vs. background classification in the context of clonogenic assays. Moreover, this work gave rise to the discovery of the *homogeneity* image feature as the most informative and discriminant feature for this classification. From a biological perspective, this result represented a relevant confirmation regarding the evidence that *homogeneity*—at the phenotypic level in a radiobiology experiment—might be a very important feature to count the number of colonies in a reliable and reproducible manner and to, finally, determine the surviving fraction of the dose response curves.

**Table 1:** The mean and standard deviation balance accuracy score (with respect to 30 datasets) for the best performing image feature and classifiers (up to and including HQC and classifiers where the score was tied with HQC), with corresponding mean and standard deviation Jaccard index and Dice coefficient, for cell lines MDA-MD-231, U87-MG, MCF7 and U251.

Cell line	Best image feature	Best classifier	Balanced accuracy	Jaccard index	Dice coefficient
MDA-MD-231	**Homogeneity**	**Helstrom Quantum Classifier**	**0.959** ± **0.036**	**0.918** ± **0.080**	**0.955** ± **0.046**
U87-MG	**Homogeneity**	**Helstrom Quantum Classifier**	**0.919** ± **0.050**	**0.790** ± **0.121**	**0.877** ± **0.078**
MCF7	L*u*v*	Gaussian Naïve Bayes	0.969 ± 0.034	0.892 ± 0.088	0.941 ± 0.050
	L*u*v*	**Helstrom Quantum Classifier**	**0.965** ± **0.033**	**0.882** ± **0.084**	**0.935** ± **0.048**
	L*u*v*	Multi Layer Perceptron	0.965 ± 0.042	0.898 ± 0.100	0.943 ± 0.059
U251	Homogeneity	SVM - RBF	0.980 ± 0.033	0.948 ± 0.079	0.971 ± 0.047
	**Homogeneity**	**Helstrom Quantum Classifier**	**0.979** ± **0.029**	**0.944** ± **0.078**	**0.970** ± **0.045**

The rows in boldface denote the results achieved by the HQC.

**Table 2:** The mean and standard deviation AUROC score (with respect to 30 datasets) for the best performing image feature and classifiers (up to and including HQC and classifiers whose score are tied with HQC), with corresponding mean and standard deviation Jaccard index and Dice coefficient, for cell lines MDA-MD-231, U87-MG, MCF7 and U251.

Cell line	Best image feature	Best classifier	AUROC	Jaccard index	Dice coefficient
MDA-MD-231	Homogeneity	Quadratic discriminant analysis	0.957 ± 0.039	0.914 ± 0.083	0.953 ± 0.048
	Homogeneity	Nearest neighbors	0.956 ± 0.039	0.914 ± 0.083	0.953 ± 0.049
	Homogeneity	Linear discriminant analysis	0.955 ± 0.049	0.912 ± 0.102	0.951 ± 0.060
	Homogeneity	Gaussian Naïve Bayes	0.954 ± 0.045	0.906 ± 0.095	0.948 ± 0.056
	**Homogeneity**	**Helstrom quantum classifier**	**0.954** ± **0.050**	**0.910** ± **0.093**	**0.950** ± **0.055**
U87-MG	**Homogeneity**	**Helstrom quantum classifier**	**0.917** ± **0.048**	**0.794** ± **0.099**	**0.882** ± **0.062**
MCF7	L*u*v*	Gaussian Naïve Bayes	0.969 ± 0.034	0.892 ± 0.088	0.941 ± 0.050
	L*u*v*	Bernoulli Naïve Bayes	0.964 ± 0.030	0.844 ± 0.128	0.910 ± 0.083
	L*u*v*	Quadratic discriminant analysis	0.961 ± 0.036	0.892 ± 0.088	0.940 ± 0.051
	L*u*v*	Linear discriminant analysis	0.961 ± 0.047	0.899 ± 0.114	0.943 ± 0.071
	L*u*v*	**Helstrom quantum classifier**	**0.960** ± **0.041**	**0.869** ± **0.139**	**0.923** ± **0.097**
	L*u*v*	SVM-linear	0.960 ± 0.052	0.894 ± 0.135	0.938 ± 0.086
U251	**Homogeneity**	**Helstrom quantum classifier**	**0.978** ± **0.027**	**0.944** ± **0.068**	**0.970** ± **0.037**
	Homogeneity	Nearest neighbors	0.978 ± 0.028	0.944 ± 0.069	0.970 ± 0.038
	Homogeneity	SVM-linear	0.978 ± 0.035	0.945 ± 0.081	0.970 ± 0.048

The rows in boldface denote the results achieved by the HQC.

**Table 3:** Performance of HQC when increasing the number of copies by an addition of one copy for cases where HQC does not outperform the other classifiers.

(a) Dataset	Hyperpar. used in exp.	Hyperpar. used in exp. and rescale=0.5, encod.=amplitude, #copies=6, class weight =weighted	(b) Dataset	Hyperpar. used in exp.	Hyperpar. used in exp. and rescale=0.5 encod.=amplitude #copies=5, class weight =weighted	(c) Dataset	Hyperpar. used in exp.	Hyperpar. used in exp. and rescale=0.5, encod.=amplitude, #copies=5, class weight =weighted	(d) Dataset	Hyperpar. used in exp.	Hyperpar. used in exp. and rescale=1.0, encod.=amplitude, #copies=6, class weight=weighted
1	0.962	0.962	1	0.913	0.935	1	0.935	0.935	1	1.000	1.000
2	0.979	0.979	2	1.000	1.000	2	1.000	1.000	2	1.000	1.000
3	0.950	0.967	3	1.000	1.000	3	1.000	1.000	3	1.000	1.000
4	1.000	1.000	4	0.938	0.938	4	0.878	0.920	4	1.000	1.000
5	0.900	0.900	5	0.941	0.941	5	0.958	0.958	5	1.000	1.000
6	0.977	0.977	6	0.958	0.958	6	1.000	1.000	6	1.000	1.000
7	0.946	0.946	7	0.982	1.000	7	0.944	1.000	7	0.974	0.974
8	0.911	0.955	8	0.938	0.980	8	0.980	0.980	8	1.000	1.000
9	0.774	0.888	9	0.982	0.982	9	0.982	0.982	9	1.000	1.000
10	0.916	0.916	10	0.918	0.918	10	0.918	0.918	10	1.000	1.000
11	0.920	0.920	11	1.000	1.000	11	1.000	1.000	11	0.975	0.975
12	1.000	1.000	12	1.000	1.000	12	0.979	1.000	12	1.000	1.000
13	0.891	0.950	13	0.958	0.979	13	0.979	0.979	13	1.000	1.000
14	1.000	1.000	14	0.960	0.960	14	0.960	0.960	14	1.000	1.000
15	0.947	0.947	15	0.941	0.979	15	0.979	0.979	15	0.962	0.962
16	0.967	0.967	16	0.984	0.984	16	0.984	0.984	16	0.975	0.975
17	1.000	1.000	17	0.981	0.981	17	1.000	1.000	17	0.896	0.917
18	0.971	0.971	18	0.940	0.940	18	0.940	0.940	18	0.927	0.927
19	1.000	1.000	19	0.985	0.985	19	0.985	0.985	19	0.946	0.946
20	1.000	1.000	20	1.000	1.000	20	1.000	1.000	20	1.000	1.000
21	1.000	1.000	21	0.967	0.967	21	0.895	0.929	21	0.950	0.950
22	0.974	0.974	22	1.000	1.000	22	1.000	1.000	22	0.974	0.974
23	0.955	0.955	23	0.984	0.984	23	0.884	0.884	23	1.000	1.000
24	1.000	1.000	24	1.000	1.000	24	1.000	1.000	24	0.980	0.980
25	1.000	1.000	25	0.875	0.875	25	0.879	0.879	25	1.000	1.000
26	0.955	0.977	26	0.946	0.946	26	0.971	0.971	26	0.983	0.983
27	0.946	0.974	27	0.911	1.000	27	0.911	1.000	27	0.982	0.982
28	0.957	0.978	28	0.982	0.982	28	0.982	0.982	28	0.895	0.895
29	0.976	1.000	29	1.000	1.000	29	0.981	1.000	29	0.980	0.980
30	0.853	0.971	30	0.969	0.969	30	0.900	0.900	30	0.982	0.982
Mean	0.954	0.969	Mean	0.965	0.973	Mean	0.960	0.969	Mean	0.979	0.980

(a) For cell line MDA-MD-231, comparison of AUROC score for HQC when increasing the number of copies to 6 for 30 homogeneity image feature datasets. (b) For cell line MCF7, comparison of balance accuracy score for HQC when increasing the number of copies to 5 for 30 L*u*v* image feature datasets. (c) For cell line MCF7, comparison of AUROC score for HQC when increasing the number of copies to 5 for 30 L*u*v* image feature datasets. (d) For cell line U251, comparison of balance accuracy score for HQC when increasing the number of copies to 6 for 30 homogeneity image feature datasets.

## Discussion and further developments

The approach proposed in this work was based on fundamental synergies between machine learning, quantum information theory and biological analysis. Overall, the achieved results are accurate and reliable. In fact, from a computational point of view, the used approach, both in terms of features and quantum-like classifier types, allowed us to obtain effective segmentation performance, with results (in particular, considering the balanced accuracy) being very similar to the reference ground-truth. The HQC being proposed, which has already shown^6^ excellent performance even when compared to other quantum-like classifiers, performed well when applied to the problem at hand. Furthermore, the extracted descriptors made it possible to further improve the classifier capabilities, compared to the *RGB* and L*u*v* color space encodings.

From a biological point of view, the results obtained would provide support in the quantification of the well area covered by cell colonies in clonogenic survival assays. Indeed, the main problems still unsolved in a radiobiology experiment for studying the effect of a cell treatment—such as irradiation or drug administration—and quantifying the surviving cells are the high variability related to the specific cell line used, as well as the subjectivity, due to operator-dependence, in evaluation methods. Therefore, it is extremely important to use an approach that allows us to quantify the cell survival in a reliable and reproducible manner to determine the dose response curves, which represent the primary study models in radiobiology.

In the future, we plan to extend the application of the proposed classification approach, which currently provides a clonogenic assay evaluation based only on the colony area alone. We aim to integrate the developed classifier into a processing pipeline together with an *ad hoc* post-processing step allowing us to accurately quantify the number of colonies grown, as required in traditional clonogenic assay evaluations.

From the classifier’s perspective, future challenges are the following: (1) develop a *pure quantum* version of the HQC (i.e., the quantum algorithm for the HQC running on quantum computers) which will enable both the advantages of a reduction in time complexity and an improvement in the accuracy at the same time, (2) investigate an optimal strategy exploiting parallel computing to allow us the use of the HQC with higher number of copies (producing a further improvement in the performance), and (3) find a multi-class generalization of the HQC (i.e., to extend the classification capability of the HQC to more than two classes). This would allow us to expand considerably the potential applications to other real-world contexts, including—but not limited to—the field of biomedical imaging.

## Supplementary Information

Supplementary material 1

## Data Availability

The software for the HQC was developed in Python and the experiment was conducted on a server with 128 GB RAM memory and 16 CPU cores. The software package is available in the public repository https://github.com/leockl/helstrom-quantum-centroid-classifier.
