# Supplementary material for: A quantum-inspired classifier for clonogenic assay evaluations
Source: Sci Rep. 2021 Feb 2;11:2830. doi: 10.1038/s41598-021-82085-8 (PMC7854718; doi:10.1038/s41598-021-82085-8)
Supplement: Supplementary file 1 — Supplementary material 1 [file 41598_2021_82085_MOESM1_ESM.pdf]

A quantum-inspired classifier for clonogenic assay evaluations - Supplementary material

Giuseppe Sergioli[1,*], Carmelo Militello[2], Leonardo Rundo[3,4], Luigi Minafra[2], Filippo Torrisi[5], Giorgio Russo[2], Keng Loon Chow[1], and Roberto Giuntini[1,6]

[1]University of Cagliari, Cagliari, Italy.
[2]Institute of Molecular Bioimaging and Physiology, Italian National Research Council, Cefalú, Palermo, Italy.
[3]Department of Radiology, University of Cambridge, Cambridge, United Kingdom.
[4]Cancer Research UK Cambridge Centre, University of Cambridge, Cambridge, United Kingdom.
[5]Department of Biomedical and Biotechnological Sciences, University of Catania, Catania, Italy.
[6]Centro Linceo Interdisciplinare "Beniamino Segre", Accademia dei Lincei, Rome, Italy.
[*]Corresponding author: giuseppe.sergioli@gmail.com

ABSTRACT

This file contains all the supplementary material regarding the experimental trials described and carried out in the manuscript "A quantum-inspired classifier for clonogenic assay evaluations". In the first section, detailed descriptions of the extracted texture features are provided. In the second section, we show the experimental results obtained for each of the four cell lines MDA-MD-231, U87-MG, MCF7, and U251, by considering the best performing image feature. The third section is devoted to show the performance of the Helstrom Quantum Classifier (HQC) on a new unseen test set. Finally, in the last section we summarize the full results of the whole experiment, for all the investigated cell lines and image features.

S1. The extracted Haralick's features

We start from the assumption that biomedical images contain information phenotype of the underlying physiopathology, which is not always easily identifiable by simple 'visual' inspection. These information can be revealed through quantitative analysis, by extracting the so called 'descriptors' in order to make it possible to acquire further knowledge on the dominion. The Gray-Level Co-occurrence Matrix (GLCM) computation is the first step to obtain the features.

Formally, let a GLCM with size $L \times L$, where L represents the maximum number of gray-levels according to a given quantization scheme, denote the second-order joint probability function $p(i, j)$ of an image region (where $i, j \in [0, 1, \ldots, L-1]$ represent a gray-level pair) after the normalization by the total number of pixels. These descriptors are generally called Haralick's features[1,2].

Given a squared window of size $\omega \times \omega$ pixels sliding over the whole image[3], we computed the following GLCM-based features (with $i, j \in [0, 1, \ldots, L-1]$):

- $\mathsf{contrast} \in [0, (L-1) \times (L-1)$ yields a measure of the intensity contrast between neighboring pixels:

$$\mathsf{contrast}(i, j) = \sum_{i=0}^{L-1} \sum_{j=0}^{L-1} |i-j|^2 \cdot p(i, j), \tag{1}$$

  $\mathsf{contrast} = 0$ for a constant image;

- $\mathsf{correlation} \in [-1, 1]$ indicates the degree of correlation between a pixel and its neighbor:

$$\mathsf{correlation}(i, j) = \frac{\sum_{i=0}^{L-1} \sum_{j=0}^{L-1} (i-\mu_x)(j-\mu_y) \cdot p_(i, j)}{\sigma_x \sigma_y}, \tag{2}$$

  where: $\mu_x = \sum_i \sum_j i \cdot p(i, j)$, $\mu_y = \sum_i \sum_j j \cdot p(i, j)$, $\sigma_x = \sum_i \sum_j (i - \mu_x) \cdot p(i, j)$, and $\sigma_y = \sum_i \sum_j (j - \mu_y) \cdot p(i, j)$ (with $\sum_i$ and $\sum_j$ denoting $\sum_{i=0}^{L-1}$ and $\sum_{j=0}^{L-1}$, respectively). This feature is 1 or -1 for a perfectly positively or negatively correlated image, respectively;

- energy $\in [0,1]$ calculates the sum of squared elements in the GLCM:

$$\text{energy}(i,j) = \sum_{i=0}^{L-1}\sum_{j=0}^{L-1} p(i,j)^2, \tag{3}$$

energy $= 1$ for a constant image;

- homogeneity $\in [0,1]$ Returns a value that measures the closeness of the distribution of elements in the GLCM to the GLCM diagonal:

$$\text{homogeneity}(i,j) = \frac{\sum_{i=0}^{L-1}\sum_{j=0}^{L-1} p(i,j)}{(1+|i-j|)}, \tag{4}$$

homogeneity $= 1$ for a diagonal GLCM.

S2. Experimental results

This section is divided into four groups, one for each cell line MDA-MD-231, U87-MG, MCF7 and U251, respectively. Each group contains two reports, the first shows: (1) the balanced accuracy score over 30 datasets (for the best performing image feature) for the HQC and the 18 competing classifiers, obtained by hypertuning the hyperparameters of each classifier in order to optimize the balanced accuracy score; (2) heatmaps of a classifier outperforming ("wins") over another classifier ("losses") out of the 30 datasets (for the best performing image feature); and (3) a table showing the averaged balanced accuracy score over the 30 datasets for each of the six image features, *RGB*, *L*u*v**, *contrast*, *correlation*, *energy* and *homogeneity*. The second report is the analogous of the first, where the role of the balanced accuracy is replaced by the AUROC score. All performance evaluation is performed using the test set. The aim of the experimental procedure is to find the most informative image feature in discriminating a pixel between a colony or a background, i.e., the image feature which maximizes the value of the balanced accuracy and the AUROC scores, respectively.

S2.1. Cell line MDA-MD-231

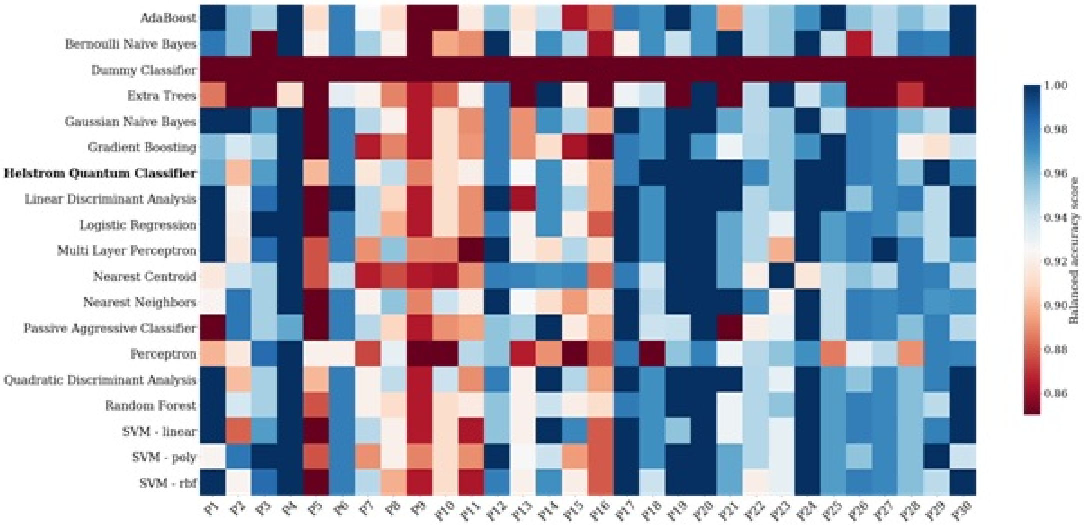

Fig. 2.1.1 | Balance accuracy score of 19 classifiers across 30 homogeneity image feature datasets for cell line MDA-MD-231.

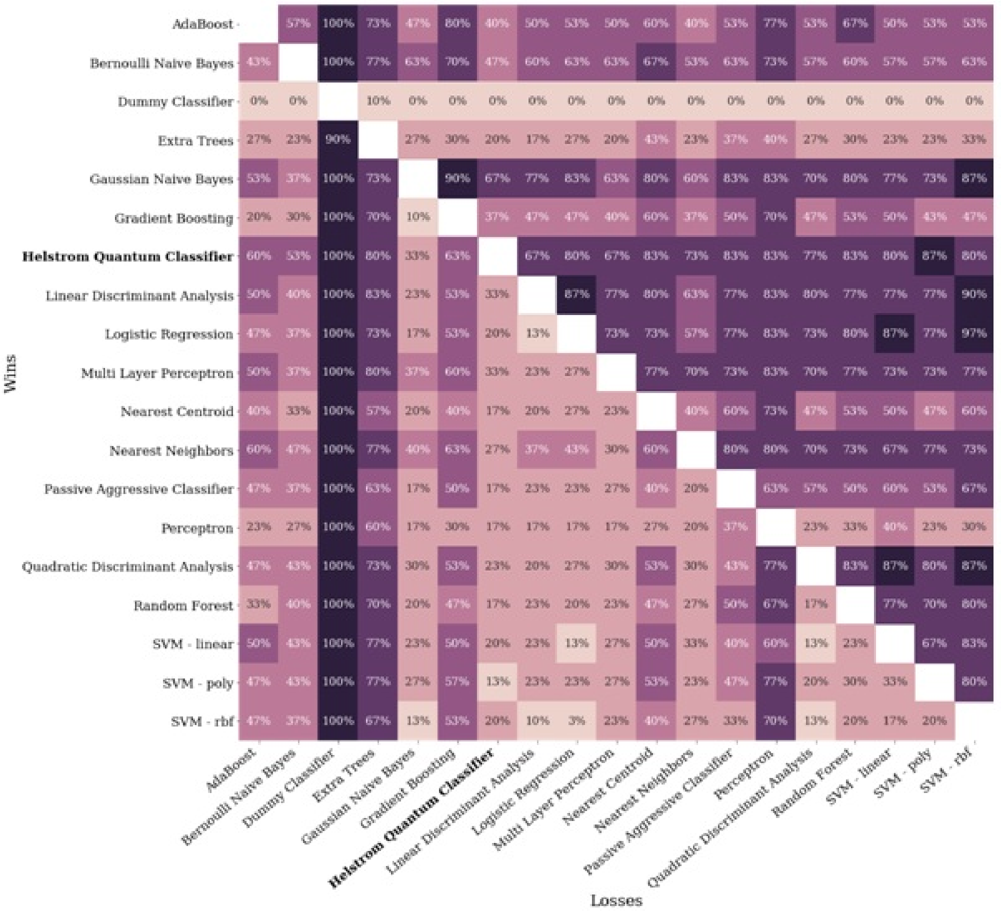

Fig. 2.1.2 | Percentage of datasets where model A ("Wins") outperformed model B ("Losses") out of 30 homogeneity image feature datasets for cell line MDA-MD-231 (balanced accuracy score).

Table 2.1.1 | The mean and standard deviation balanced accuracy score (with respect to 30 datasets) for cell line MDA-MD-231

	**Image features**					
**Classifiers**	**RGB**	**L*u*v***	**Contrast**	**Correlation**	**Energy**	**Homogeneity**
AdaBoost	0.861 ± 0.088	0.878 ± 0.065	0.898 ± 0.065	0.738 ± 0.110	0.936 ± 0.052	0.942 ± 0.046
Bernoulli Naive Bayes	0.841 ± 0.060	0.855 ± 0.079	0.840 ± 0.097	0.630 ± 0.126	0.854 ± 0.087	0.940 ± 0.057
Dummy Classifier	0.507 ± 0.047	0.513 ± 0.056	0.506 ± 0.069	0.518 ± 0.042	0.497 ± 0.069	0.485 ± 0.060
Extra Trees	0.822 ± 0.120	0.839 ± 0.141	0.709 ± 0.163	0.616 ± 0.121	0.767 ± 0.169	0.851 ± 0.139
Gaussian Naive Bayes	0.855 ± 0.063	0.874 ± 0.084	0.886 ± 0.063	0.711 ± 0.102	0.923 ± 0.067	0.954 ± 0.045
Gradient Boosting	0.876 ± 0.076	0.874 ± 0.069	0.899 ± 0.063	0.835 ± 0.113	0.941 ± 0.047	0.933 ± 0.050
**Helstrom Quantum Classifier**	0.895 ± 0.058	0.883 ± 0.069	0.902 ± 0.070	0.775 ± 0.081	0.938 ± 0.047	**0.959 ± 0.036**
Linear Discriminant Analysis	0.850 ± 0.066	0.865 ± 0.080	0.837 ± 0.075	0.660 ± 0.122	0.860 ± 0.089	0.955 ± 0.049
Logistic Regression	0.882 ± 0.061	0.879 ± 0.086	0.883 ± 0.066	0.657 ± 0.131	0.932 ± 0.056	0.951 ± 0.045
Multi Layer Perceptron	0.890 ± 0.055	0.885 ± 0.069	0.901 ± 0.069	0.833 ± 0.086	0.942 ± 0.052	0.940 ± 0.091
Nearest Centroid	0.839 ± 0.058	0.868 ± 0.078	0.846 ± 0.070	0.686 ± 0.114	0.867 ± 0.063	0.941 ± 0.044
Nearest Neighbors	0.890 ± 0.069	0.875 ± 0.064	0.903 ± 0.073	0.808 ± 0.070	0.942 ± 0.049	0.953 ± 0.040
Passive Aggressive Classifier	0.831 ± 0.105	0.809 ± 0.113	0.828 ± 0.118	0.607 ± 0.137	0.907 ± 0.062	0.935 ± 0.052
Perceptron	0.790 ± 0.117	0.832 ± 0.106	0.831 ± 0.112	0.631 ± 0.103	0.914 ± 0.054	0.916 ± 0.060
Quadratic Discriminant Analysis	0.872 ± 0.065	0.876 ± 0.074	0.876 ± 0.076	0.731 ± 0.112	0.925 ± 0.066	0.957 ± 0.039
Random Forest	0.874 ± 0.078	0.883 ± 0.074	0.919 ± 0.058	0.827 ± 0.119	0.944 ± 0.043	0.951 ± 0.036
SVM - linear	0.880 ± 0.063	0.873 ± 0.078	0.880 ± 0.071	0.655 ± 0.141	0.929 ± 0.052	0.949 ± 0.046
SVM - poly	0.883 ± 0.055	0.892 ± 0.069	0.899 ± 0.065	0.811 ± 0.084	0.926 ± 0.050	0.950 ± 0.042
SVM - rbf	0.876 ± 0.062	0.874 ± 0.084	0.890 ± 0.069	0.662 ± 0.134	0.931 ± 0.052	0.948 ± 0.046

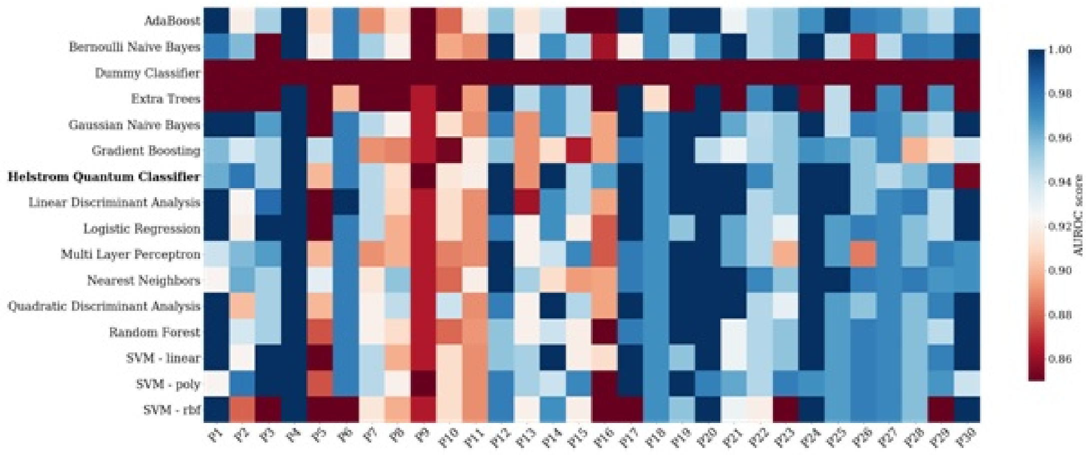

Fig. 2.1.3 | AUROC score of 19 classifiers across 30 homogeneity image feature datasets for cell line MDA-MD-231.

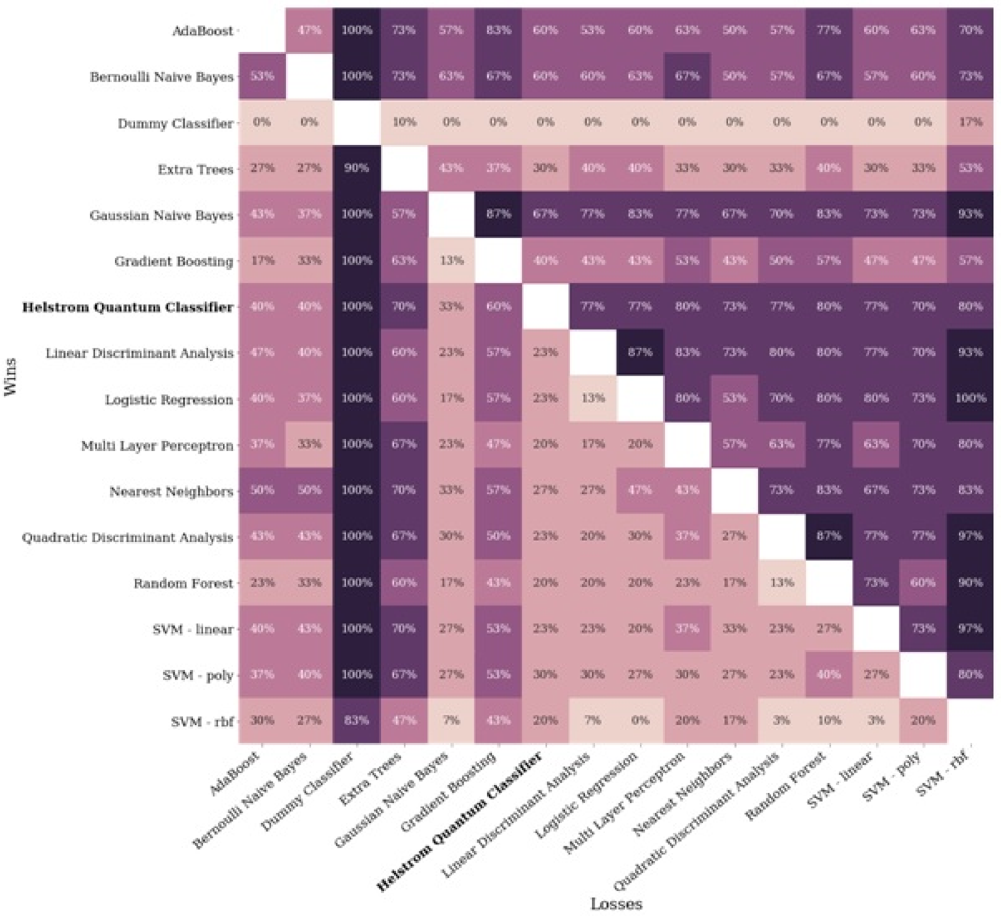

Fig. 2.1.4 | Percentage of datasets where model A ("Wins") outperformed model B ("Losses") out of 30 homogeneity image feature datasets for cell line MDA-MD-231 (AUROC score).

Table 2.1.2 | The mean and standard deviation AUROC score (with respect to 30 datasets) for cell line MDA-MD-231

	Image features					
**Classifiers**	**RGB**	**L*u*v***	**Contrast**	**Correlation**	**Energy**	**Homogeneity**
AdaBoost	0.874 ± 0.076	0.881 ± 0.067	0.898 ± 0.063	0.739 ± 0.130	0.932 ± 0.051	0.943 ± 0.048
Bernoulli Naive Bayes	0.841 ± 0.060	0.855 ± 0.079	0.840 ± 0.097	0.630 ± 0.126	0.854 ± 0.087	0.940 ± 0.057
Dummy Classifier	0.505 ± 0.027	0.498 ± 0.072	0.484 ± 0.046	0.497 ± 0.070	0.503 ± 0.077	0.497 ± 0.049
Extra Trees	0.813 ± 0.132	0.812 ± 0.159	0.678 ± 0.162	0.615 ± 0.127	0.740 ± 0.178	0.837 ± 0.159
Gaussian Naive Bayes	0.855 ± 0.063	0.874 ± 0.084	0.886 ± 0.063	0.711 ± 0.102	0.923 ± 0.067	0.954 ± 0.045
Gradient Boosting	0.880 ± 0.074	0.868 ± 0.076	0.902 ± 0.066	0.834 ± 0.114	0.930 ± 0.056	0.935 ± 0.039
**Helstrom Quantum Classifier**	0.879 ± 0.081	0.884 ± 0.066	0.905 ± 0.062	0.787 ± 0.082	0.927 ± 0.058	**0.954 ± 0.050**
Linear Discriminant Analysis	0.850 ± 0.066	0.865 ± 0.080	0.837 ± 0.075	0.660 ± 0.122	0.860 ± 0.089	0.955 ± 0.049
Logistic Regression	0.872 ± 0.072	0.874 ± 0.088	0.886 ± 0.068	0.660 ± 0.138	0.927 ± 0.051	0.950 ± 0.044
Multi Layer Perceptron	0.889 ± 0.056	0.889 ± 0.057	0.894 ± 0.065	0.804 ± 0.104	0.932 ± 0.041	0.946 ± 0.042
Nearest Neighbors	0.883 ± 0.070	0.877 ± 0.066	0.902 ± 0.064	0.800 ± 0.075	0.937 ± 0.049	0.956 ± 0.039
Quadratic Discriminant Analysis	0.872 ± 0.065	0.876 ± 0.074	0.876 ± 0.076	0.731 ± 0.112	0.925 ± 0.066	0.957 ± 0.039
Random Forest	0.881 ± 0.068	0.878 ± 0.077	0.912 ± 0.062	0.817 ± 0.117	0.943 ± 0.040	0.946 ± 0.043
SVM - linear	0.879 ± 0.064	0.876 ± 0.076	0.881 ± 0.073	0.648 ± 0.135	0.914 ± 0.049	0.953 ± 0.043
SVM - poly	0.880 ± 0.058	0.864 ± 0.082	0.897 ± 0.064	0.805 ± 0.087	0.920 ± 0.052	0.950 ± 0.047
SVM - rbf	0.845 ± 0.115	0.860 ± 0.104	0.831 ± 0.147	0.652 ± 0.132	0.879 ± 0.138	0.843 ± 0.193

Results exclude Nearest Centroid, Passive Aggressive Classifier and Perceptron.

S2.2. Cell line U87-MG

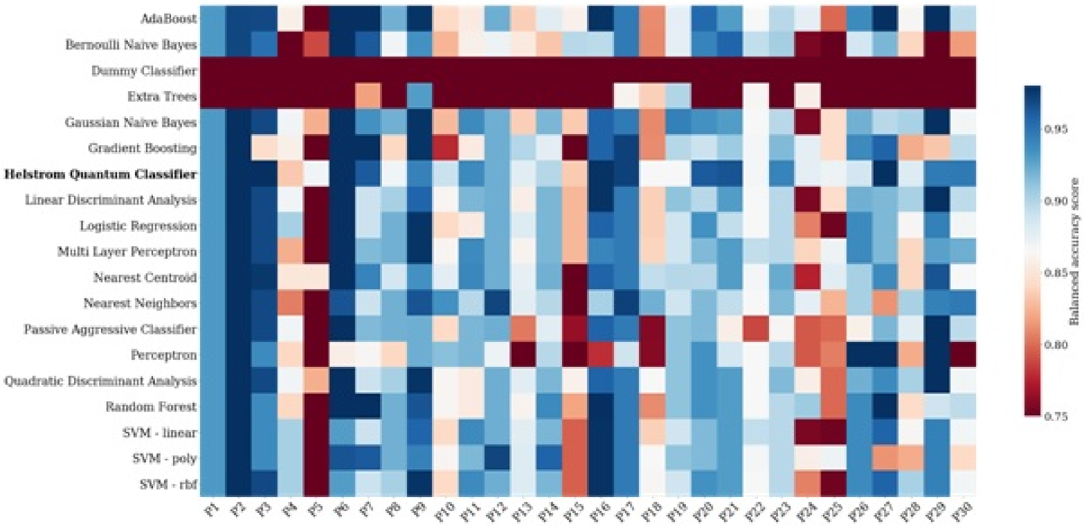

Fig. 2.2.1 | Balance accuracy score of 19 classifiers across 30 homogeneity image feature datasets for cell line U87-MG.

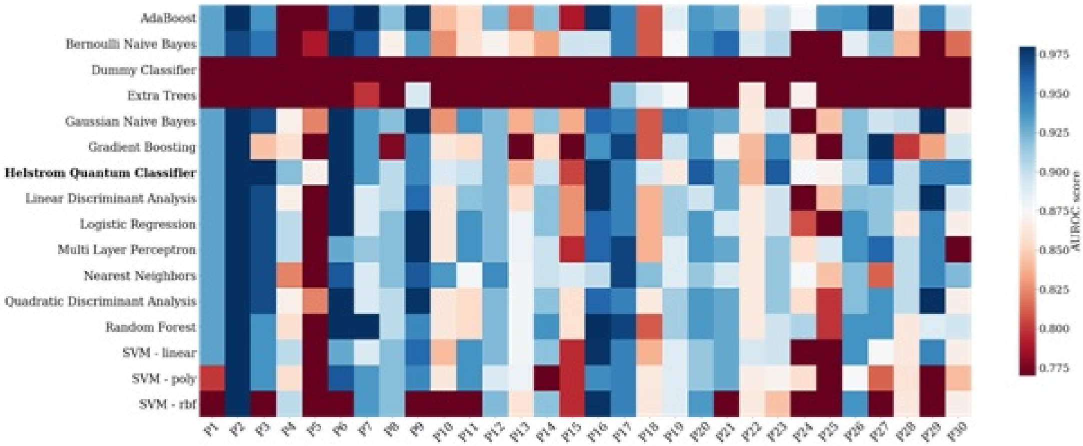

Fig. 2.2.3 | AUROC score of 19 classifiers across 30 homogeneity image feature datasets for cell line U87-MG.

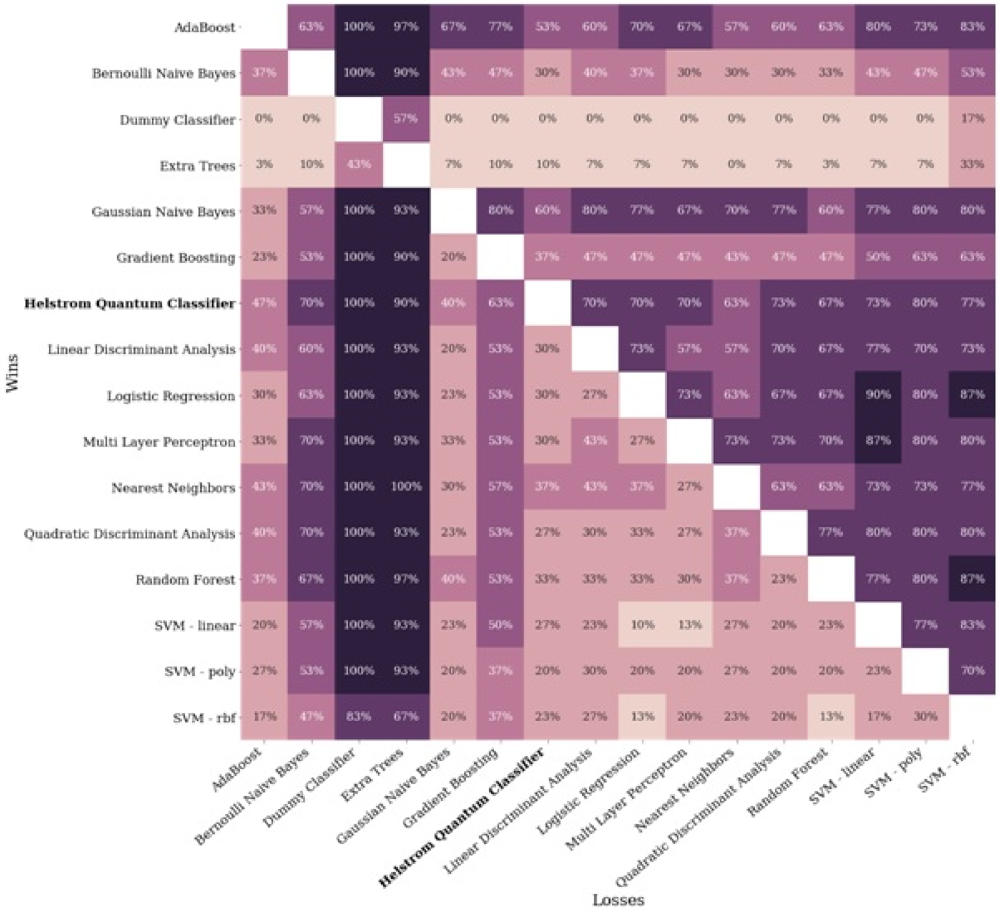

Fig. 2.2.4 | Percentage of datasets where model A ("Wins") outperformed model B ("Losses") out of 30 homogeneity image feature datasets for cell line U87-MG (AUROC score).

Table 2.2.2 | The mean and standard deviation AUROC score (with respect to 30 datasets) for cell line U87-MG						
	Image features					
Classifiers	RGB	L*u*v*	Contrast	Correlation	Energy	Homogeneity
AdaBoost	0.776 ± 0.117	0.779 ± 0.110	0.864 ± 0.060	0.704 ± 0.105	0.873 ± 0.082	0.898 ± 0.077
Bernoulli Naive Bayes	0.765 ± 0.095	0.788 ± 0.084	0.841 ± 0.070	0.629 ± 0.088	0.771 ± 0.126	0.869 ± 0.082
Dummy Classifier	0.504 ± 0.050	0.504 ± 0.056	0.512 ± 0.055	0.480 ± 0.076	0.505 ± 0.055	0.516 ± 0.058
Extra Trees	0.577 ± 0.098	0.583 ± 0.120	0.529 ± 0.078	0.515 ± 0.043	0.525 ± 0.068	0.613 ± 0.156
Gaussian Naive Bayes	0.734 ± 0.089	0.752 ± 0.096	0.780 ± 0.086	0.674 ± 0.074	0.890 ± 0.065	0.905 ± 0.059
Gradient Boosting	0.815 ± 0.091	0.827 ± 0.085	0.877 ± 0.064	0.758 ± 0.095	0.879 ± 0.075	0.871 ± 0.085
**Helstrom Quantum Classifier**	0.791 ± 0.099	0.792 ± 0.090	0.838 ± 0.066	0.768 ± 0.075	0.902 ± 0.057	**0.917 ± 0.048**
Linear Discriminant Analysis	0.752 ± 0.095	0.736 ± 0.085	0.740 ± 0.076	0.639 ± 0.086	0.842 ± 0.107	0.900 ± 0.065
Logistic Regression	0.770 ± 0.100	0.764 ± 0.102	0.805 ± 0.085	0.636 ± 0.086	0.877 ± 0.071	0.897 ± 0.070
Multi Layer Perceptron	0.792 ± 0.109	0.784 ± 0.115	0.840 ± 0.067	0.768 ± 0.079	0.877 ± 0.099	0.901 ± 0.070
Nearest Neighbors	0.793 ± 0.105	0.793 ± 0.090	0.847 ± 0.044	0.739 ± 0.086	0.884 ± 0.076	0.902 ± 0.054
Quadratic Discriminant Analysis	0.773 ± 0.100	0.750 ± 0.102	0.782 ± 0.100	0.667 ± 0.074	0.879 ± 0.081	0.908 ± 0.050
Random Forest	0.808 ± 0.106	0.803 ± 0.100	0.861 ± 0.059	0.763 ± 0.083	0.900 ± 0.070	0.902 ± 0.068
SVM - linear	0.764 ± 0.100	0.751 ± 0.113	0.798 ± 0.088	0.591 ± 0.103	0.891 ± 0.065	0.888 ± 0.076
SVM - poly	0.745 ± 0.119	0.746 ± 0.111	0.780 ± 0.084	0.720 ± 0.091	0.865 ± 0.095	0.864 ± 0.090
SVM - rbf	0.756 ± 0.123	0.741 ± 0.114	0.682 ± 0.157	0.575 ± 0.096	0.859 ± 0.130	0.770 ± 0.179

Results exclude Nearest Centroid, Passive Aggressive Classifier and Perceptron.

S2.3. Cell line MCF7

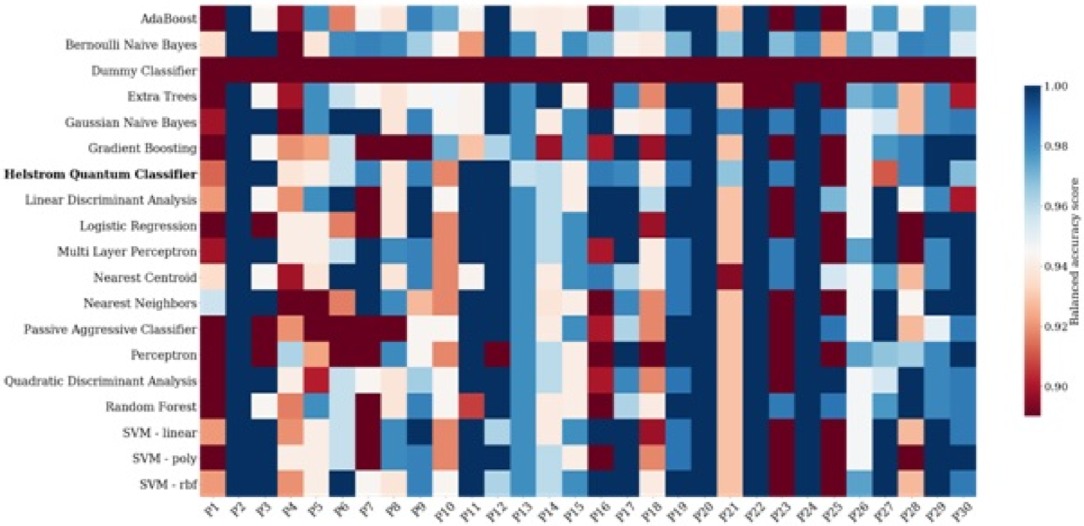

Fig. 2.3.1 | Balance accuracy score of 19 classifiers across 30 L*u*v* image feature datasets for cell line MCF7.

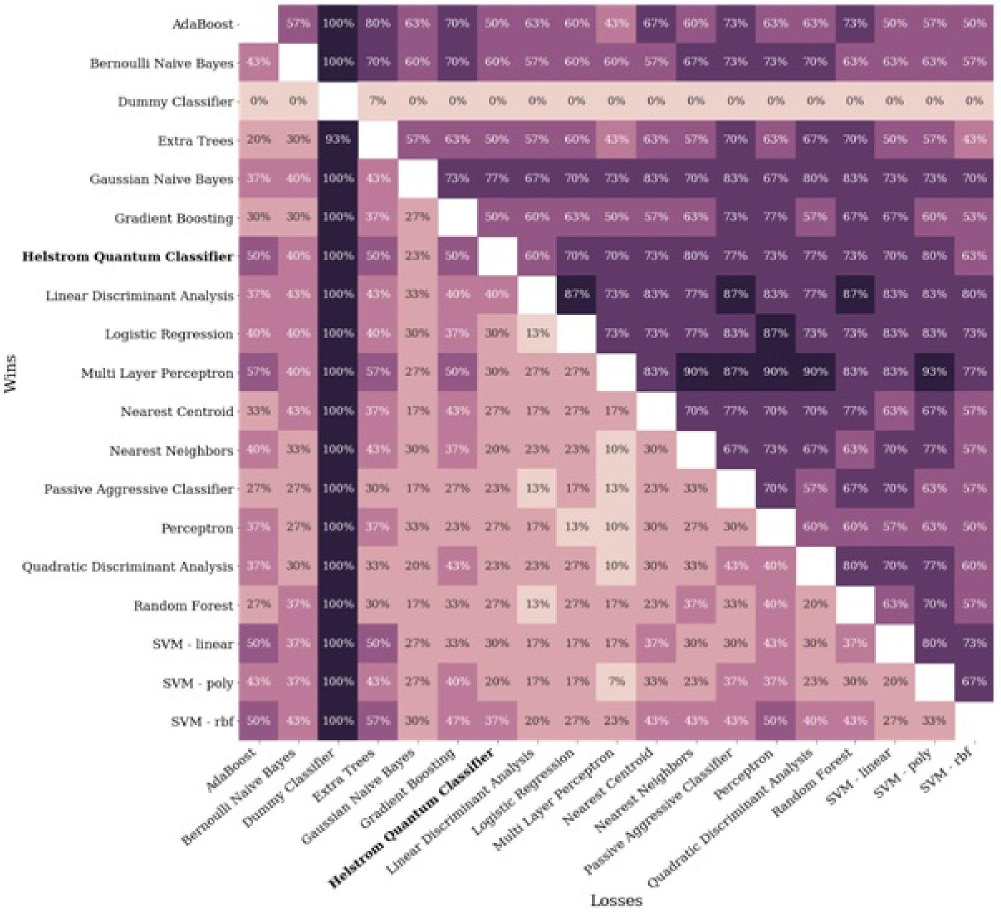

Fig. 2.3.2 | Percentage of datasets where model A ("Wins") outperformed model B ("Losses") out of 30 L*u*v* image feature datasets for cell line MCF7 (balanced accuracy score).

Table 2.3.1 | The mean and standard deviation balanced accuracy score (with respect to 30 datasets) for cell line MCF7

	Image features					
**Classifiers**	**RGB**	**L*u*v***	**Contrast**	**Correlation**	**Energy**	**Homogeneity**
AdaBoost	0.959 ± 0.046	0.940 ± 0.070	0.827 ± 0.106	0.699 ± 0.099	0.851 ± 0.111	0.875 ± 0.092
Bernoulli Naive Bayes	0.951 ± 0.048	0.964 ± 0.030	0.717 ± 0.124	0.661 ± 0.107	0.718 ± 0.133	0.837 ± 0.118
Dummy Classifier	0.505 ± 0.064	0.509 ± 0.041	0.494 ± 0.048	0.484 ± 0.044	0.500 ± 0.057	0.491 ± 0.044
Extra Trees	0.858 ± 0.150	0.914 ± 0.125	0.507 ± 0.037	0.526 ± 0.061	0.523 ± 0.078	0.585 ± 0.124
Gaussian Naive Bayes	0.943 ± 0.043	0.969 ± 0.034	0.641 ± 0.095	0.715 ± 0.091	0.886 ± 0.069	0.882 ± 0.078
Gradient Boosting	0.934 ± 0.080	0.939 ± 0.062	0.814 ± 0.103	0.706 ± 0.088	0.844 ± 0.093	0.866 ± 0.110
**Helstrom Quantum Classifier**	0.949 ± 0.052	**0.965 ± 0.033**	0.798 ± 0.109	0.775 ± 0.081	0.875 ± 0.059	0.892 ± 0.065
Linear Discriminant Analysis	0.947 ± 0.047	0.961 ± 0.047	0.626 ± 0.097	0.685 ± 0.098	0.781 ± 0.139	0.874 ± 0.095
Logistic Regression	0.946 ± 0.055	0.948 ± 0.060	0.638 ± 0.107	0.674 ± 0.095	0.866 ± 0.097	0.856 ± 0.096
Multi Layer Perceptron	0.946 ± 0.052	0.965 ± 0.042	0.760 ± 0.106	0.718 ± 0.089	0.867 ± 0.089	0.856 ± 0.111
Nearest Centroid	0.923 ± 0.056	0.964 ± 0.032	0.770 ± 0.117	0.739 ± 0.095	0.851 ± 0.068	0.890 ± 0.063
Nearest Neighbors	0.951 ± 0.056	0.947 ± 0.063	0.769 ± 0.109	0.759 ± 0.075	0.861 ± 0.075	0.854 ± 0.101
Passive Aggressive Classifier	0.945 ± 0.048	0.943 ± 0.051	0.690 ± 0.120	0.616 ± 0.145	0.787 ± 0.145	0.770 ± 0.154
Perceptron	0.938 ± 0.056	0.923 ± 0.094	0.652 ± 0.167	0.630 ± 0.145	0.774 ± 0.122	0.795 ± 0.149
Quadratic Discriminant Analysis	0.959 ± 0.050	0.961 ± 0.036	0.638 ± 0.096	0.710 ± 0.111	0.883 ± 0.072	0.872 ± 0.089
Random Forest	0.948 ± 0.064	0.956 ± 0.044	0.816 ± 0.112	0.740 ± 0.097	0.851 ± 0.113	0.860 ± 0.110
SVM - linear	0.955 ± 0.050	0.956 ± 0.052	0.654 ± 0.138	0.696 ± 0.107	0.868 ± 0.110	0.873 ± 0.108
SVM - poly	0.940 ± 0.054	0.949 ± 0.060	0.762 ± 0.108	0.730 ± 0.080	0.862 ± 0.078	0.874 ± 0.105
SVM - rbf	0.950 ± 0.053	0.962 ± 0.048	0.681 ± 0.134	0.697 ± 0.108	0.868 ± 0.112	0.877 ± 0.102

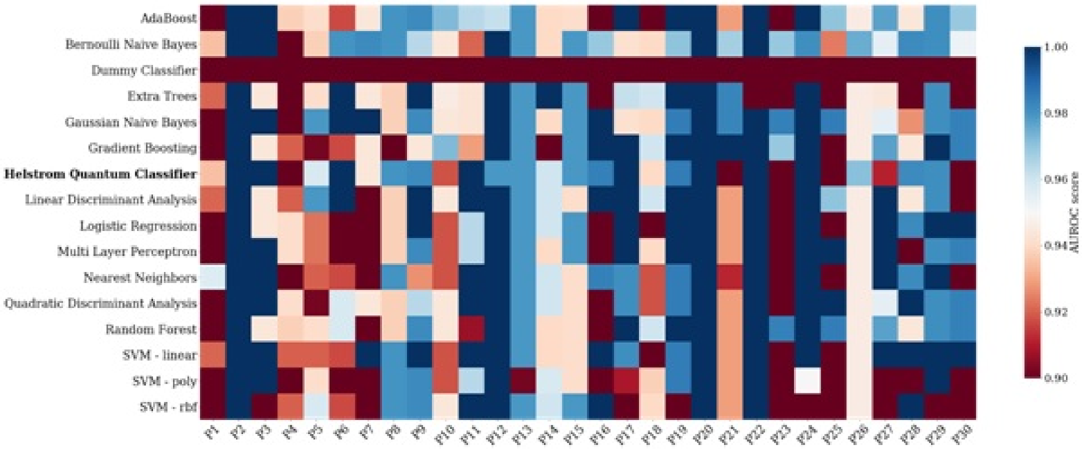

Fig. 2.3.3 | AUROC score of 19 classifiers across 30 L*u*v* image feature datasets for cell line MCF7.

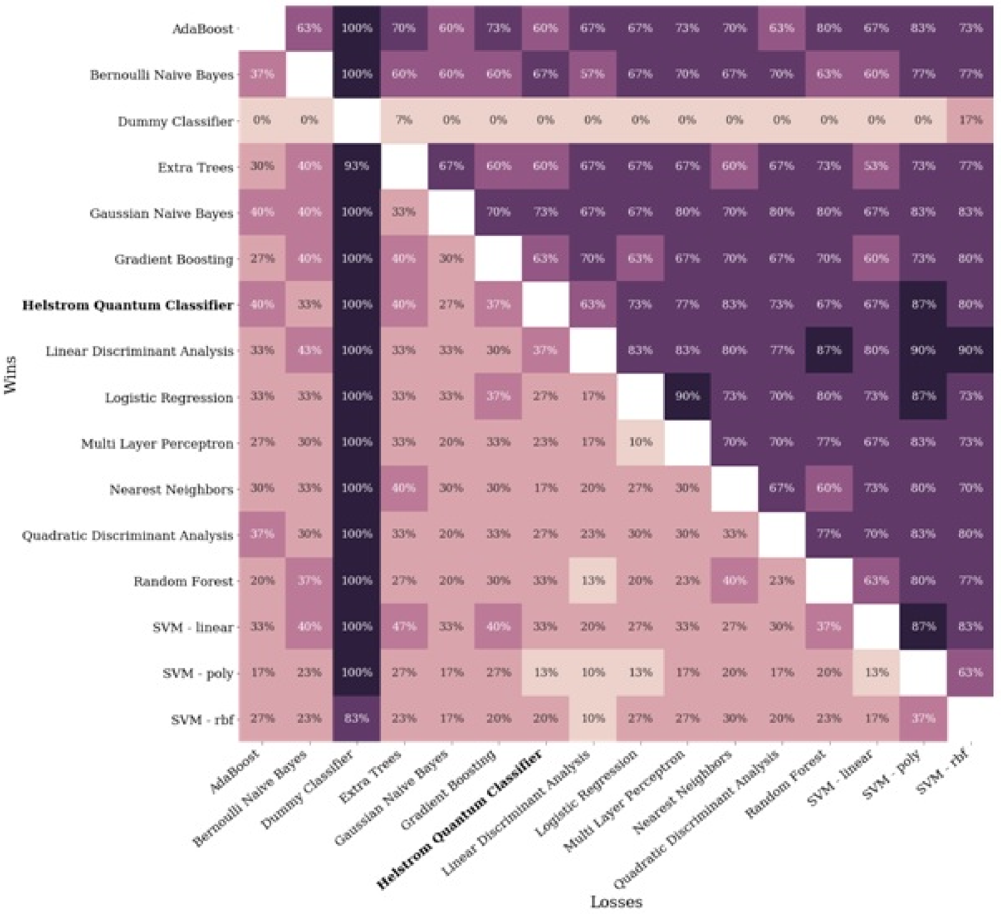

Fig. 2.3.4 | Percentage of datasets where model A ("Wins") outperformed model B ("Losses") out of 30 L*u*v* image feature datasets for cell line MCF7 (AUROC score).

Table 2.3.2 | The mean and standard deviation AUROC score (with respect to 30 datasets) for cell line MCF7

	Image features					
**Classifiers**	**RGB**	**L*u*v***	**Contrast**	**Correlation**	**Energy**	**Homogeneity**
AdaBoost	0.944 ± 0.076	0.951 ± 0.058	0.821 ± 0.110	0.704 ± 0.107	0.845 ± 0.103	0.857 ± 0.102
Bernoulli Naive Bayes	0.951 ± 0.048	0.964 ± 0.030	0.717 ± 0.124	0.661 ± 0.107	0.718 ± 0.133	0.837 ± 0.118
Dummy Classifier	0.475 ± 0.066	0.506 ± 0.073	0.504 ± 0.041	0.506 ± 0.051	0.496 ± 0.069	0.493 ± 0.051
Extra Trees	0.856 ± 0.148	0.918 ± 0.129	0.505 ± 0.025	0.511 ± 0.038	0.509 ± 0.043	0.527 ± 0.068
Gaussian Naive Bayes	0.943 ± 0.043	0.969 ± 0.034	0.641 ± 0.095	0.715 ± 0.091	0.886 ± 0.069	0.882 ± 0.078
Gradient Boosting	0.911 ± 0.112	0.952 ± 0.060	0.773 ± 0.121	0.719 ± 0.091	0.820 ± 0.092	0.837 ± 0.117
**Helstrom Quantum Classifier**	0.955 ± 0.045	**0.960 ± 0.041**	0.753 ± 0.120	0.766 ± 0.090	0.866 ± 0.078	0.883 ± 0.071
Linear Discriminant Analysis	0.947 ± 0.047	0.961 ± 0.047	0.626 ± 0.096	0.685 ± 0.098	0.780 ± 0.141	0.874 ± 0.095
Logistic Regression	0.943 ± 0.062	0.950 ± 0.054	0.637 ± 0.108	0.672 ± 0.095	0.853 ± 0.115	0.848 ± 0.107
Multi Layer Perceptron	0.953 ± 0.056	0.950 ± 0.054	0.770 ± 0.120	0.700 ± 0.103	0.850 ± 0.082	0.869 ± 0.104
Nearest Neighbors	0.948 ± 0.045	0.947 ± 0.061	0.767 ± 0.115	0.751 ± 0.087	0.862 ± 0.080	0.850 ± 0.110
Quadratic Discriminant Analysis	0.959 ± 0.050	0.961 ± 0.036	0.638 ± 0.096	0.710 ± 0.111	0.883 ± 0.072	0.872 ± 0.089
Random Forest	0.949 ± 0.066	0.953 ± 0.057	0.811 ± 0.116	0.733 ± 0.100	0.838 ± 0.133	0.852 ± 0.104
SVM - linear	0.946 ± 0.054	0.960 ± 0.052	0.635 ± 0.135	0.687 ± 0.113	0.849 ± 0.126	0.858 ± 0.130
SVM - poly	0.887 ± 0.093	0.927 ± 0.063	0.713 ± 0.125	0.682 ± 0.113	0.841 ± 0.107	0.866 ± 0.121
SVM - rbf	0.872 ± 0.144	0.859 ± 0.176	0.586 ± 0.111	0.601 ± 0.121	0.839 ± 0.142	0.717 ± 0.200

Results exclude Nearest Centroid, Passive Aggressive Classifier and Perceptron.

S2.4. Cell line U251

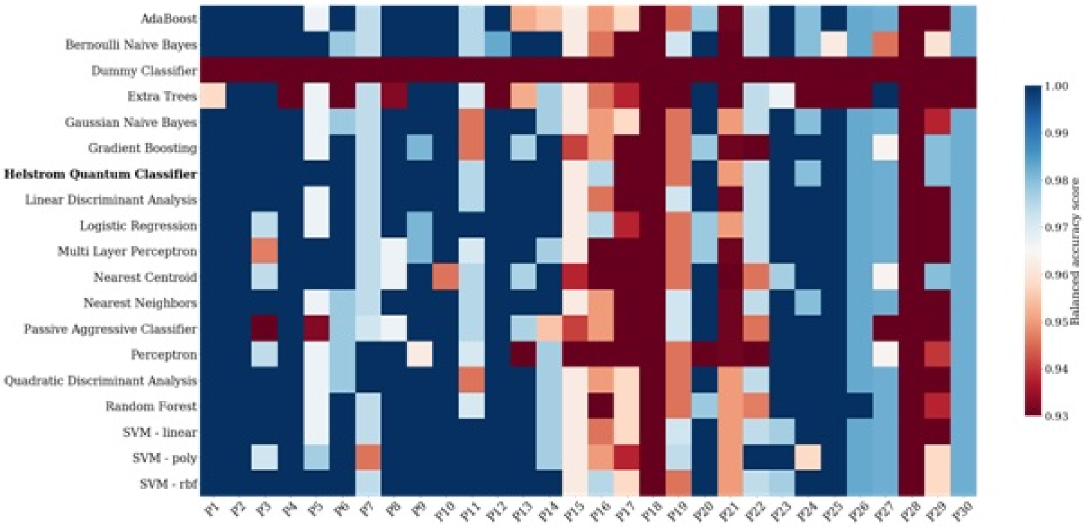

Fig. 2.4.1 | Balance accuracy score of 19 classifiers across 30 homogeneity image feature datasets for cell line U251.

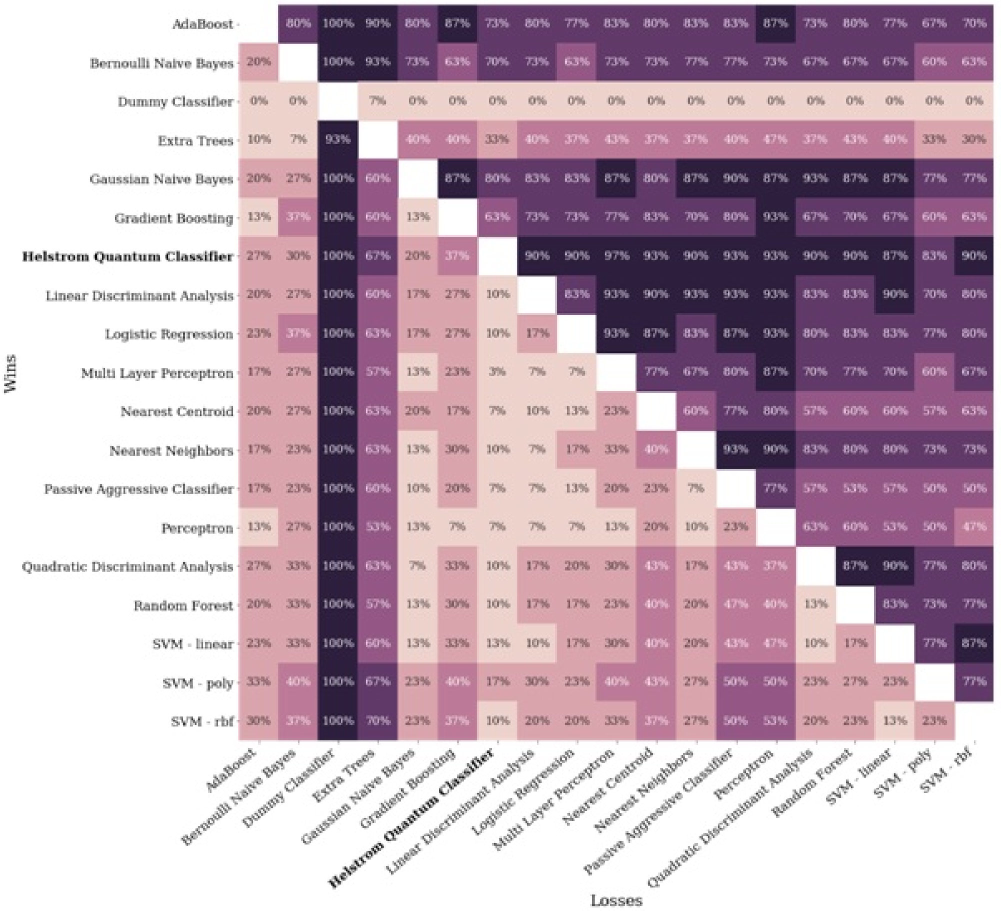

Fig. 2.4.2 | Percentage of datasets where model A ("Wins") outperformed model B ("Losses") out of 30 homogeneity image feature datasets for cell line U251 (balanced accuracy score).

Table 2.4.1 | The mean and standard deviation balanced accuracy score (with respect to 30 datasets) for cell line U251

	Image features					
**Classifiers**	**RGB**	**L*u*v***	**Contrast**	**Correlation**	**Energy**	**Homogeneity**
AdaBoost	0.911 ± 0.049	0.909 ± 0.046	0.969 ± 0.027	0.771 ± 0.081	0.968 ± 0.045	0.972 ± 0.032
Bernoulli Naive Bayes	0.890 ± 0.047	0.903 ± 0.043	0.934 ± 0.055	0.649 ± 0.108	0.859 ± 0.118	0.967 ± 0.059
Dummy Classifier	0.504 ± 0.068	0.494 ± 0.058	0.495 ± 0.049	0.485 ± 0.057	0.495 ± 0.051	0.509 ± 0.069
Extra Trees	0.832 ± 0.126	0.865 ± 0.098	0.714 ± 0.188	0.571 ± 0.110	0.692 ± 0.206	0.867 ± 0.164
Gaussian Naive Bayes	0.908 ± 0.052	0.912 ± 0.051	0.969 ± 0.030	0.698 ± 0.084	0.964 ± 0.033	0.976 ± 0.027
Gradient Boosting	0.917 ± 0.049	0.907 ± 0.054	0.958 ± 0.034	0.845 ± 0.064	0.965 ± 0.039	0.970 ± 0.035
**Helstrom Quantum Classifier**	0.917 ± 0.045	0.907 ± 0.039	0.966 ± 0.032	0.792 ± 0.070	0.973 ± 0.024	**0.979 ± 0.029**
Linear Discriminant Analysis	0.892 ± 0.062	0.904 ± 0.047	0.916 ± 0.065	0.674 ± 0.087	0.935 ± 0.058	0.975 ± 0.036
Logistic Regression	0.911 ± 0.051	0.918 ± 0.044	0.965 ± 0.030	0.677 ± 0.087	0.964 ± 0.031	0.976 ± 0.031
Multi Layer Perceptron	0.915 ± 0.045	0.924 ± 0.046	0.965 ± 0.035	0.837 ± 0.087	0.968 ± 0.034	0.969 ± 0.035
Nearest Centroid	0.892 ± 0.051	0.892 ± 0.048	0.927 ± 0.033	0.694 ± 0.091	0.923 ± 0.048	0.967 ± 0.038
Nearest Neighbors	0.914 ± 0.046	0.907 ± 0.050	0.969 ± 0.032	0.836 ± 0.078	0.974 ± 0.036	0.974 ± 0.033
Passive Aggressive Classifier	0.875 ± 0.070	0.881 ± 0.070	0.951 ± 0.050	0.575 ± 0.111	0.958 ± 0.047	0.959 ± 0.046
Perceptron	0.902 ± 0.054	0.889 ± 0.060	0.945 ± 0.060	0.586 ± 0.117	0.951 ± 0.039	0.952 ± 0.056
Quadratic Discriminant Analysis	0.923 ± 0.053	0.924 ± 0.051	0.972 ± 0.028	0.712 ± 0.085	0.969 ± 0.033	0.976 ± 0.028
Random Forest	0.926 ± 0.048	0.918 ± 0.047	0.973 ± 0.033	0.815 ± 0.086	0.970 ± 0.044	0.974 ± 0.034
SVM - linear	0.916 ± 0.047	0.914 ± 0.051	0.970 ± 0.027	0.656 ± 0.103	0.975 ± 0.028	0.976 ± 0.035
SVM - poly	0.925 ± 0.052	0.911 ± 0.064	0.972 ± 0.027	0.821 ± 0.072	0.970 ± 0.031	0.978 ± 0.025
SVM - rbf	0.913 ± 0.051	0.917 ± 0.053	0.969 ± 0.032	0.667 ± 0.100	0.976 ± 0.027	0.980 ± 0.033

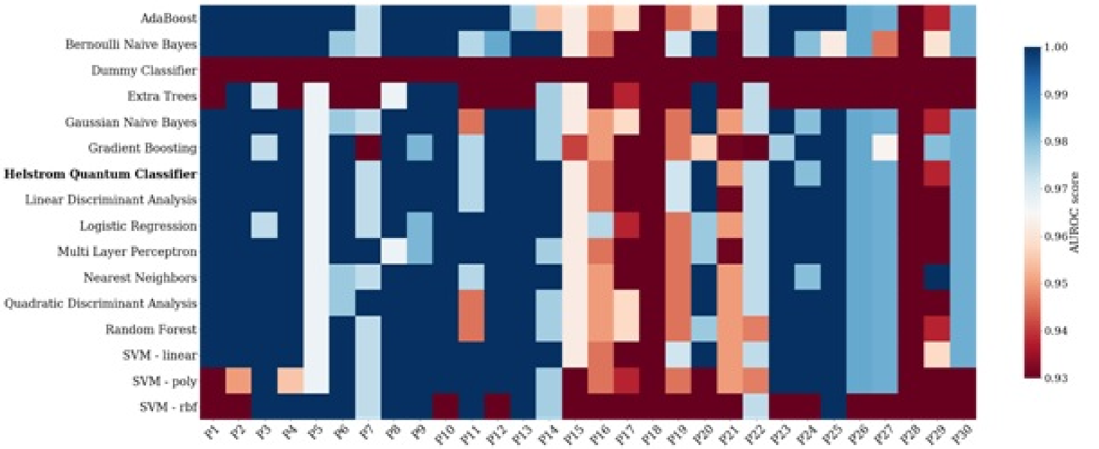

Fig. 2.4.3 | AUROC score of 19 classifiers across 30 homogeneity image feature datasets for cell line U251.

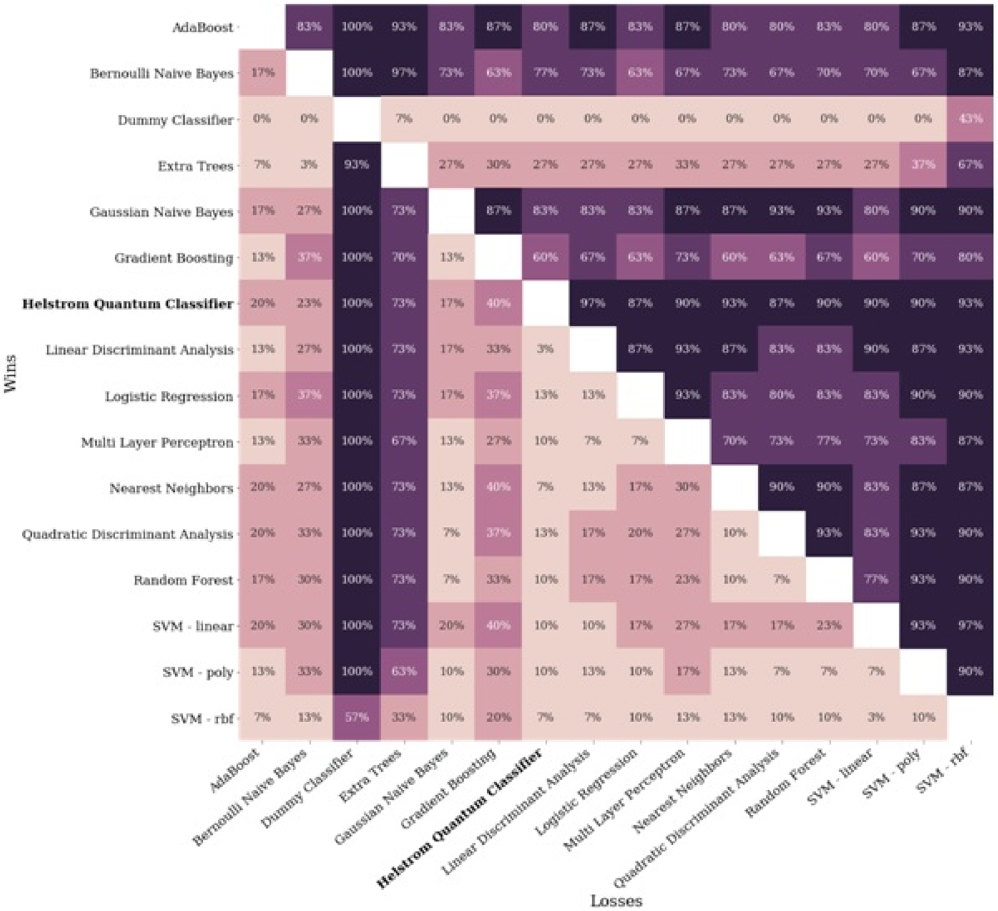

Fig. 2.4.4 | Percentage of datasets where model A ("Wins") outperformed model B ("Losses") out of 30 homogeneity image feature datasets for cell line U251 (AUROC score).

Table 2.4.2 | The mean and standard deviation AUROC score (with respect to 30 datasets) for cell line U251

	Image features					
Classifiers	RGB	L*u*v*	Contrast	Correlation	Energy	Homogeneity
AdaBoost	0.920 ± 0.046	0.911 ± 0.048	0.971 ± 0.027	0.741 ± 0.099	0.967 ± 0.045	0.975 ± 0.034
Bernoulli Naive Bayes	0.890 ± 0.047	0.903 ± 0.043	0.934 ± 0.055	0.649 ± 0.108	0.859 ± 0.118	0.967 ± 0.059
Dummy Classifier	0.500 ± 0.050	0.492 ± 0.049	0.499 ± 0.059	0.502 ± 0.054	0.503 ± 0.055	0.511 ± 0.055
Extra Trees	0.808 ± 0.152	0.843 ± 0.110	0.653 ± 0.177	0.558 ± 0.098	0.673 ± 0.186	0.838 ± 0.155
Gaussian Naive Bayes	0.908 ± 0.052	0.912 ± 0.051	0.969 ± 0.030	0.698 ± 0.084	0.964 ± 0.033	0.976 ± 0.027
Gradient Boosting	0.924 ± 0.052	0.906 ± 0.046	0.964 ± 0.031	0.830 ± 0.060	0.966 ± 0.040	0.968 ± 0.034
**Helstrom Quantum Classifier**	0.899 ± 0.057	0.907 ± 0.053	0.948 ± 0.063	0.797 ± 0.093	0.966 ± 0.031	**0.978 ± 0.027**
Linear Discriminant Analysis	0.892 ± 0.062	0.904 ± 0.047	0.916 ± 0.065	0.674 ± 0.087	0.935 ± 0.058	0.975 ± 0.036
Logistic Regression	0.911 ± 0.050	0.917 ± 0.046	0.962 ± 0.030	0.676 ± 0.088	0.962 ± 0.036	0.975 ± 0.034
Multi Layer Perceptron	0.921 ± 0.046	0.918 ± 0.051	0.965 ± 0.034	0.830 ± 0.093	0.962 ± 0.034	0.971 ± 0.040
Nearest Neighbors	0.914 ± 0.051	0.907 ± 0.050	0.964 ± 0.040	0.823 ± 0.062	0.972 ± 0.036	0.978 ± 0.028
Quadratic Discriminant Analysis	0.923 ± 0.053	0.924 ± 0.051	0.972 ± 0.028	0.712 ± 0.085	0.969 ± 0.033	0.976 ± 0.028
Random Forest	0.921 ± 0.050	0.925 ± 0.049	0.975 ± 0.025	0.836 ± 0.076	0.973 ± 0.038	0.972 ± 0.035
SVM - linear	0.916 ± 0.049	0.909 ± 0.047	0.962 ± 0.030	0.651 ± 0.103	0.971 ± 0.030	0.978 ± 0.035
SVM - poly	0.897 ± 0.063	0.901 ± 0.062	0.952 ± 0.061	0.801 ± 0.089	0.969 ± 0.030	0.955 ± 0.051
SVM - rbf	0.849 ± 0.150	0.833 ± 0.156	0.776 ± 0.221	0.634 ± 0.114	0.871 ± 0.189	0.723 ± 0.240

Results exclude Nearest Centroid, Passive Aggressive Classifier and Perceptron.

S3. Performance of HQC on a new unseen test set (for the best image feature for each cell line)

In this sub-experiment the trained HQC model was tested on a new unseen test set extracted from the remaining 99.8% of the datasets. This experiment was done by randomly selecting 10 datasets (out of the 30 datasets) from the best performing image feature for each of the four cell lines. We show a comparison of the performance on this new unseen test set against the performance on the test set from the 0.2% random sample used in the main experiment.

Table 3.1 | Balance accuracy and AUROC score for HQC on the test set used in the experiment and a new unseen test set for 10 randomly selected homogeneity image feature datasets for cell line MDA-MD-231

Datasets	Balanced accuracy Test set used in the experiment	Balanced accuracy New unseen test set	AUROC Test set used in the experiment	AUROC New unseen test set
P5	0.900	0.943	0.900	0.943
P6	0.977	0.921	0.977	0.921
P7	0.916	0.897	0.946	0.896
P8	0.944	0.937	0.911	0.943
P14	0.971	0.935	1.000	0.933
P15	0.921	0.869	0.947	0.888
P23	0.955	0.968	0.955	0.967
P26	0.977	0.947	0.955	0.944
P27	0.974	0.953	0.946	0.951
P30	0.971	0.951	0.853	0.811
Mean	0.951	0.932	0.939	0.920

Table 3.2 | Balance accuracy and AUROC score for HQC on the test set used in the experiment and a new unseen test set for 10 randomly selected homogeneity image feature datasets for cell line U87-MG

Datasets	Balanced accuracy Test set used in the experiment	Balanced accuracy New unseen test set	AUROC Test set used in the experiment	AUROC New unseen test set
P1	0.931	0.947	0.931	0.947
P2	0.985	0.953	0.985	0.954
P5	0.869	0.908	0.869	0.908
P9	0.943	0.928	0.943	0.940
P10	0.891	0.894	0.891	0.897
P14	0.898	0.868	0.898	0.870
P21	0.964	0.910	0.929	0.901
P22	0.867	0.868	0.839	0.870
P25	0.871	0.893	0.871	0.893
P27	0.980	0.869	0.960	0.871
Mean	0.920	0.904	0.912	0.905

Table 3.3 | Balance accuracy and AUROC score for HQC on the test set used in the experiment and a new unseen test set for 10 randomly selected L*u*v* image feature datasets for cell line MCF7

Datasets	Balanced accuracy Test set used in the experiment	Balanced accuracy New unseen test set	AUROC Test set used in the experiment	AUROC New unseen test set
P6	0.958	0.955	1.000	0.958
P8	0.938	0.949	0.980	0.951
P9	0.982	0.958	0.982	0.955
P13	0.958	0.966	0.979	0.970
P14	0.960	0.978	0.960	0.978
P16	0.984	0.959	0.984	0.951
P19	0.985	0.977	0.985	0.976
P23	0.984	0.967	0.884	0.947
P25	0.875	0.867	0.879	0.886
P26	0.946	0.980	0.971	0.946
Mean	0.957	0.956	0.960	0.952

Table 3.4 | Balance accuracy and AUROC score for HQC on the test set used in the experiment and a new unseen test set for 10 randomly selected homogeneity image feature datasets for cell line U251

Datasets	Balanced accuracy Test set used in the experiment	Balanced accuracy New unseen test set	AUROC Test set used in the experiment	AUROC New unseen test set
P2	1.000	0.985	1.000	0.977
P7	0.974	0.962	0.974	0.962
P15	0.962	0.956	0.962	0.898
P18	0.927	0.971	0.927	0.971
P20	1.000	0.960	1.000	0.959
P21	0.950	0.964	0.950	0.964
P22	0.974	0.941	0.974	0.948
P27	0.982	0.965	0.982	0.973
P29	0.980	0.926	0.938	0.932
P30	0.982	0.976	0.982	0.968
Mean	0.973	0.961	0.969	0.955

S4. Experimental results - extended version

This section contains tables showing the experimental results for the balanced accuracy and AUROC scores for the six image features *RGB*, *L*u*v**, *contrast*, *correlation*, *energy* and *homogeneity*, for each of the four cell lines MDA-MD-231, U87-MG, MCF7 and U251 respectively.

Table 4.1 | Balanced accuracy score for 30 RGB image feature datasets for cell line MDA-MD-231

Classifiers	P1	P2	P3	P4	P5	P6	P7	P8	P9	P10	P11	P12	P13	P14	P15	P16	P17	P18	P19	P20	P21	P22	P23	P24	P25	P26	P27	P28	P29	P30
AdaBoost	0.855	0.647	0.586	0.820	0.917	0.808	0.851	0.938	0.907	0.789	0.838	0.921	0.858	0.925	0.734	0.938	0.896	0.858	0.875	0.866	0.978	1.000	0.890	0.917	0.863	0.881	0.796	0.885	0.980	0.807
Bernoulli Naive Bayes	0.738	0.756	0.755	0.897	0.889	0.720	0.850	0.906	0.857	0.741	0.814	0.891	0.916	0.866	0.778	0.878	0.946	0.865	0.761	0.875	0.828	0.843	0.904	0.863	0.807	0.894	0.789	0.856	0.878	0.871
Dummy Classifier	0.612	0.500	0.500	0.500	0.500	0.506	0.500	0.558	0.418	0.500	0.500	0.500	0.568	0.500	0.461	0.500	0.544	0.484	0.500	0.599	0.418	0.500	0.561	0.500	0.599	0.428	0.473	0.500	0.468	0.510
Extra Trees	0.855	0.577	0.500	0.835	0.917	0.742	0.938	0.938	0.893	0.750	0.813	0.935	0.864	0.866	0.764	0.625	0.941	0.923	0.795	0.900	0.857	0.825	0.890	0.890	0.864	0.500	0.820	0.885	0.897	0.866
Gaussian Naive Bayes	0.795	0.816	0.804	0.897	0.917	0.742	0.850	0.938	0.821	0.789	0.787	0.891	0.955	0.871	0.742	0.938	0.946	0.904	0.795	0.900	0.750	0.864	0.890	0.917	0.807	0.913	0.789	0.856	0.918	0.846
Gradient Boosting	0.917	0.763	0.685	0.851	0.890	0.753	0.818	0.969	0.885	0.882	0.705	0.871	0.935	0.896	0.792	0.938	0.971	0.897	0.858	0.866	0.943	1.000	0.890	0.947	0.863	0.931	0.851	0.899	0.980	0.837
**Helstrom Quantum Classifier**	0.918	0.816	0.820	0.866	0.889	0.841	0.914	0.938	0.964	0.851	0.893	0.885	0.897	0.925	0.734	0.980	0.921	0.923	0.875	0.950	0.871	0.979	0.935	0.889	0.835	0.963	0.812	0.943	0.980	0.841
Linear Discriminant Analysis	0.837	0.857	0.734	0.842	0.917	0.764	0.866	0.938	0.835	0.781	0.789	0.870	0.916	0.900	0.778	0.897	0.971	0.885	0.688	0.850	0.835	0.938	0.825	0.833	0.756	0.881	0.812	0.899	0.940	0.866
Logistic Regression	0.857	0.920	0.817	0.890	0.917	0.798	0.882	0.938	0.871	0.757	0.839	0.899	0.935	0.896	0.778	0.938	0.946	0.885	0.795	0.900	0.835	0.979	0.890	0.917	0.782	0.981	0.820	0.907	0.980	0.921
Multi Layer Perceptron	0.857	0.861	0.817	0.882	0.917	0.798	0.859	0.945	0.964	0.859	0.760	0.849	0.916	0.921	0.770	0.940	0.921	0.942	0.875	0.866	0.893	0.979	0.935	0.920	0.889	0.931	0.875	0.871	0.980	0.896
Nearest Centroid	0.797	0.812	0.804	0.874	0.889	0.741	0.897	0.906	0.857	0.789	0.788	0.848	0.916	0.791	0.742	0.897	0.946	0.827	0.716	0.850	0.764	0.825	0.890	0.861	0.808	0.913	0.812	0.856	0.918	0.846
Nearest Neighbors	0.857	0.668	0.702	0.890	0.917	0.855	0.859	0.976	0.929	0.844	0.867	0.849	0.909	0.921	0.814	0.938	0.946	0.942	0.875	0.946	0.943	0.958	0.890	0.918	0.863	0.931	0.851	0.943	0.980	0.916
Passive Aggressive Classifier	0.793	0.784	0.868	0.914	0.863	0.508	0.890	0.890	0.762	0.741	0.732	0.834	0.878	0.744	0.778	0.918	0.946	0.885	0.750	0.875	0.770	0.979	0.916	0.833	0.890	0.963	0.827	0.814	1.000	0.593
Perceptron	0.857	0.647	0.500	0.882	0.917	0.655	0.750	0.897	0.821	0.621	0.785	0.685	0.684	0.816	0.562	0.920	0.825	0.734	0.778	0.694	0.814	0.941	0.753	0.861	0.756	0.931	0.851	0.877	0.980	0.891
Quadratic Discriminant Analysis	0.877	0.760	0.884	0.890	0.889	0.752	0.921	0.914	0.835	0.812	0.893	0.863	0.916	0.896	0.778	0.960	0.971	0.904	0.750	0.841	0.814	0.979	0.935	0.838	0.808	0.913	0.882	0.899	0.980	0.807
Random Forest	0.875	0.784	0.618	0.914	0.917	0.730	0.969	0.938	0.871	0.772	0.839	0.871	0.935	0.925	0.756	0.918	0.971	0.923	0.875	0.866	0.893	0.941	0.890	0.917	0.863	0.931	0.827	0.863	0.980	0.841
SVM - linear	0.857	0.798	0.884	0.914	0.917	0.798	0.859	0.938	0.835	0.757	0.788	0.856	0.935	0.925	0.778	0.938	0.971	0.904	0.795	0.871	0.835	0.979	0.890	0.917	0.863	0.963	0.820	0.907	0.980	0.921
SVM - poly	0.835	0.784	0.868	0.859	0.917	0.876	0.914	0.938	0.964	0.820	0.814	0.871	0.832	0.950	0.778	0.837	0.846	0.942	0.858	0.941	0.835	0.962	0.890	0.944	0.947	0.876	0.890	0.835	0.980	0.896
SVM - rbf	0.857	0.857	0.884	0.914	0.889	0.753	0.859	0.938	0.857	0.757	0.788	0.842	0.935	0.925	0.778	0.938	0.971	0.885	0.795	0.871	0.835	0.979	0.890	0.889	0.863	0.963	0.812	0.907	0.980	0.871

Table 4.2 | Balanced accuracy score for 30 L*u*v* image feature datasets for cell line MDA-MD-231

Classifiers	P1	P2	P3	P4	P5	P6	P7	P8	P9	P10	P11	P12	P13	P14	P15	P16	P17	P18	P19	P20	P21	P22	P23	P24	P25	P26	P27	P28	P29	P30
AdaBoost	0.734	0.789	0.875	0.943	0.890	0.804	0.895	0.796	0.866	0.898	0.946	0.967	0.795	0.906	0.757	0.769	0.898	0.941	0.849	0.921	0.864	0.921	0.978	0.835	0.952	0.871	0.947	0.913	0.918	0.895
Bernoulli Naive Bayes	0.904	0.866	0.844	0.907	0.906	0.804	0.784	0.750	0.701	0.712	0.918	0.944	0.692	0.859	0.741	0.824	0.932	0.878	0.842	0.941	0.727	0.921	0.899	0.938	0.881	0.871	0.946	0.863	0.918	0.946
Dummy Classifier	0.399	0.500	0.606	0.500	0.500	0.484	0.430	0.456	0.583	0.500	0.458	0.500	0.500	0.582	0.500	0.543	0.517	0.500	0.505	0.500	0.509	0.487	0.453	0.653	0.606	0.500	0.500	0.500	0.500	0.621
Extra Trees	0.844	0.632	0.500	0.943	0.906	0.784	0.864	0.796	0.820	0.500	0.918	0.967	0.738	0.952	0.850	0.500	0.932	0.979	0.778	0.946	0.778	0.921	0.957	0.921	0.929	0.750	1.000	0.863	0.958	0.947
Gaussian Naive Bayes	0.897	0.796	0.775	0.929	0.938	0.801	0.865	0.796	0.820	0.732	0.918	0.967	0.707	0.882	0.821	0.681	0.932	0.958	0.885	0.941	0.781	0.895	0.957	0.938	0.976	0.821	1.000	0.899	0.958	0.947
Gradient Boosting	0.825	0.804	0.853	0.943	0.914	0.766	0.895	0.796	0.890	0.815	0.867	0.911	0.795	0.859	0.762	0.698	0.865	1.000	0.828	0.891	0.836	0.921	0.964	0.914	0.952	0.921	0.921	0.921	0.938	0.947
**Helstrom Quantum Classifier**	0.844	0.859	0.838	0.943	0.938	0.822	0.892	0.796	0.905	0.733	0.920	0.933	0.708	0.882	0.707	0.879	0.898	0.979	0.885	0.916	0.892	0.921	0.935	0.914	0.905	0.929	0.972	0.899	0.938	0.918
Linear Discriminant Analysis	0.890	0.890	0.791	0.907	0.906	0.819	0.865	0.750	0.804	0.752	0.917	0.967	0.740	0.906	0.821	0.626	0.909	0.938	0.849	0.921	0.753	0.895	0.935	0.906	0.929	0.821	0.946	0.891	0.938	0.974
Logistic Regression	0.844	0.859	0.938	0.943	0.938	0.804	0.838	0.796	0.835	0.733	0.944	0.967	0.708	0.906	0.821	0.626	0.898	0.958	0.842	0.946	0.781	0.921	0.978	0.938	0.921	0.893	0.972	0.899	0.958	0.974
Multi Layer Perceptron	0.844	0.859	0.938	0.885	0.938	0.804	0.838	0.766	0.929	0.837	0.946	0.967	0.763	0.827	0.757	0.824	0.921	0.979	0.820	0.812	0.838	0.921	0.957	0.906	0.952	0.964	0.972	0.877	0.938	0.974
Nearest Centroid	0.916	0.921	0.844	0.907	0.938	0.760	0.865	0.750	0.851	0.653	0.864	0.967	0.723	0.851	0.821	0.790	0.876	0.917	0.863	0.916	0.754	0.895	0.957	0.938	0.929	0.821	0.972	0.913	0.938	0.918
Nearest Neighbors	0.890	0.882	0.822	0.899	0.938	0.784	0.864	0.800	0.859	0.813	0.893	0.967	0.802	0.859	0.766	0.714	0.898	0.941	0.863	0.837	0.835	0.920	0.921	0.921	0.929	0.929	0.974	0.835	0.918	0.974
Passive Aggressive Classifier	0.942	0.820	0.875	0.943	0.945	0.745	0.753	0.712	0.701	0.580	0.944	0.830	0.638	0.668	0.678	0.643	0.732	0.920	0.755	0.691	0.751	0.868	0.935	0.890	0.976	0.857	0.918	0.792	0.792	0.974
Perceptron	0.935	0.796	0.838	0.929	0.859	0.825	0.868	0.753	0.763	0.673	0.892	0.865	0.804	0.906	0.746	0.483	0.886	0.774	0.863	0.866	0.567	0.895	0.929	0.938	0.921	0.821	0.944	0.792	0.918	0.918
Quadratic Discriminant Analysis	0.935	0.757	0.922	0.943	0.906	0.804	0.839	0.825	0.859	0.732	0.918	0.967	0.802	0.906	0.762	0.698	0.932	0.920	0.863	0.891	0.781	0.895	0.978	0.914	0.952	0.857	0.947	0.899	0.938	0.947
Random Forest	0.871	0.882	0.838	0.907	0.938	0.843	0.895	0.800	0.874	0.775	0.920	0.967	0.763	0.882	0.787	0.698	0.932	0.979	0.863	0.921	0.727	0.921	0.978	0.945	0.976	0.871	0.947	0.899	0.938	0.947
SVM - linear	0.890	0.890	0.838	0.907	0.906	0.804	0.839	0.771	0.772	0.753	0.944	0.944	0.723	0.906	0.821	0.698	0.909	0.958	0.842	0.946	0.753	0.921	0.913	0.938	0.952	0.857	0.972	0.899	0.938	0.974
SVM - poly	0.890	0.827	0.838	0.943	0.945	0.843	0.921	0.796	0.897	0.775	0.944	0.933	0.716	0.859	0.821	0.790	0.898	0.979	0.863	0.921	0.838	0.921	0.957	0.969	0.976	0.893	1.000	0.885	0.938	0.974
SVM - rbf	0.890	0.921	0.822	0.907	0.906	0.825	0.867	0.771	0.772	0.733	0.944	0.967	0.762	0.906	0.771	0.626	0.932	0.958	0.842	0.946	0.781	0.895	0.935	0.938	0.952	0.857	0.972	0.899	0.938	0.974

Table 4.3 | Balanced accuracy score for 30 contrast image feature datasets for cell line MDA-MD-231

Classifiers	P1	P2	P3	P4	P5	P6	P7	P8	P9	P10	P11	P12	P13	P14	P15	P16	P17	P18	P19	P20	P21	P22	P23	P24	P25	P26	P27	P28	P29	P30
AdaBoost	0.835	0.823	0.879	0.918	0.866	0.744	0.795	0.909	0.945	0.978	0.881	0.975	0.890	0.890	0.804	0.784	0.933	0.921	0.909	0.974	0.972	0.925	0.920	0.891	0.921	0.799	0.971	0.905	0.969	1.000
Bernoulli Naive Bayes	0.817	0.657	0.500	0.917	0.765	0.846	0.778	0.888	0.812	0.943	0.882	0.875	0.890	0.839	0.739	0.869	0.933	0.929	0.909	0.918	0.890	0.850	0.784	0.771	0.944	0.824	0.925	0.921	0.699	0.886
Dummy Classifier	0.500	0.500	0.500	0.488	0.567	0.556	0.500	0.500	0.500	0.500	0.500	0.500	0.591	0.500	0.596	0.500	0.608	0.500	0.500	0.535	0.500	0.500	0.484	0.544	0.417	0.356	0.500	0.307	0.440	0.685
Extra Trees	0.500	0.500	0.500	0.750	0.625	0.588	0.914	0.911	0.531	0.500	0.719	0.946	0.556	0.864	0.801	0.500	0.500	0.820	0.727	0.722	0.579	0.950	0.731	0.746	0.933	0.588	0.921	0.571	0.844	0.932
Gaussian Naive Bayes	0.817	0.886	0.862	0.918	0.789	0.782	0.881	0.888	0.875	0.821	0.914	0.975	0.890	0.839	0.878	0.800	0.933	0.945	0.864	0.946	0.841	0.950	0.840	0.796	0.977	0.824	1.000	0.897	1.000	0.955
Gradient Boosting	0.855	0.841	0.879	0.918	0.914	0.719	0.929	0.842	0.921	0.943	0.905	0.916	0.835	0.918	0.825	0.784	0.967	0.890	0.909	1.000	1.000	0.921	0.864	0.866	0.955	0.857	0.941	0.874	0.969	1.000
**Helstrom Quantum Classifier**	0.911	0.834	0.812	0.917	0.882	0.699	0.857	0.865	0.906	1.000	0.952	0.950	0.836	0.813	0.804	0.800	0.933	0.952	0.909	0.946	0.972	0.925	0.899	0.921	0.977	0.857	0.975	0.969	1.000	0.977
Linear Discriminant Analysis	0.800	0.795	0.643	0.917	0.695	0.807	0.802	0.855	0.844	0.857	0.882	0.900	0.863	0.865	0.801	0.800	0.867	0.842	0.773	0.972	0.762	0.875	0.724	0.771	0.967	0.794	0.950	0.859	0.905	0.909
Logistic Regression	0.873	0.886	0.879	0.890	0.789	0.715	0.818	0.888	0.906	0.835	0.952	0.975	0.890	0.839	0.822	0.800	0.900	0.914	0.818	0.972	0.893	0.925	0.843	0.821	0.944	0.824	0.975	0.929	1.000	0.977
Multi Layer Perceptron	0.909	0.744	0.845	0.917	0.859	0.803	0.921	0.955	0.938	1.000	0.929	0.975	0.807	0.813	0.804	0.784	0.933	0.921	0.909	0.944	0.972	0.900	0.899	0.921	0.977	0.828	0.975	0.881	1.000	0.977
Nearest Centroid	0.782	0.675	0.795	0.918	0.772	0.841	0.802	0.855	0.875	0.790	0.929	0.871	0.836	0.867	0.822	0.784	0.900	0.835	0.818	0.946	0.839	0.900	0.766	0.766	0.967	0.744	0.975	0.859	0.905	0.932
Nearest Neighbors	0.909	0.664	0.895	0.944	0.835	0.782	0.952	0.944	0.938	0.964	0.929	0.975	0.835	0.813	0.825	0.784	0.933	0.921	0.909	0.944	0.974	0.896	0.878	0.921	0.932	0.882	1.000	0.952	1.000	0.955
Passive Aggressive Classifier	0.617	0.748	0.862	0.890	0.664	0.794	0.850	0.865	0.668	0.585	0.881	0.871	0.890	0.787	0.801	0.784	0.900	0.859	0.935	0.833	0.500	0.950	0.808	0.887	0.977	0.941	0.975	0.802	0.969	0.944
Perceptron	0.631	0.834	0.829	0.944	0.772	0.719	0.833	0.585	0.804	0.689	0.881	0.975	0.890	0.734	0.801	0.900	0.877	0.945	0.864	0.863	0.705	0.946	0.742	0.599	0.921	0.824	0.975	0.921	0.952	0.977
Quadratic Discriminant Analysis	0.817	0.806	0.719	0.918	0.757	0.837	0.826	0.877	0.851	0.964	0.882	0.946	0.864	0.813	0.798	0.800	0.933	0.945	0.909	0.972	0.867	0.950	0.857	0.746	0.977	0.794	1.000	0.929	0.976	0.955
Random Forest	0.855	0.886	0.895	0.918	0.921	0.769	0.881	0.888	0.945	1.000	0.952	0.975	0.889	0.865	0.885	0.784	0.967	0.945	0.909	0.974	0.972	0.950	0.920	0.896	0.977	0.857	1.000	0.905	1.000	0.977
SVM - linear	0.835	0.823	0.879	0.890	0.765	0.749	0.787	0.888	0.875	0.943	0.929	0.975	0.890	0.865	0.784	0.800	0.900	0.969	0.864	0.946	0.890	0.900	0.825	0.796	0.977	0.794	1.000	0.929	0.969	0.977
SVM - poly	0.873	0.886	0.845	0.946	0.820	0.837	0.818	0.865	0.938	0.978	0.905	0.853	0.751	0.785	0.784	0.869	0.933	0.921	0.909	0.944	0.974	0.950	0.920	0.891	0.977	0.916	1.000	0.905	1.000	0.977
SVM - rbf	0.817	0.823	0.879	0.890	0.796	0.749	0.826	0.888	0.906	0.943	0.952	0.975	0.890	0.918	0.784	0.800	0.900	0.945	0.864	0.972	0.918	0.900	0.864	0.821	0.977	0.794	1.000	0.929	1.000	0.977

Table 4.4 | Balanced accuracy score for 30 correlation image feature datasets for cell line MDA-MD-231

Classifiers	P1	P2	P3	P4	P5	P6	P7	P8	P9	P10	P11	P12	P13	P14	P15	P16	P17	P18	P19	P20	P21	P22	P23	P24	P25	P26	P27	P28	P29	P30
AdaBoost	0.757	0.583	0.519	0.728	0.801	0.701	0.785	0.558	0.853	0.757	0.565	0.832	0.765	0.814	0.519	0.648	0.897	0.784	0.833	0.900	0.877	0.770	0.742	0.598	0.745	0.863	0.850	0.665	0.710	0.730
Bernoulli Naive Bayes	0.683	0.583	0.500	0.623	0.500	0.467	0.781	0.455	0.502	0.708	0.719	0.586	0.734	0.756	0.421	0.500	0.740	0.784	0.595	0.803	0.425	0.503	0.713	0.674	0.721	0.776	0.818	0.458	0.656	0.730
Dummy Classifier	0.602	0.596	0.570	0.538	0.516	0.500	0.596	0.426	0.506	0.500	0.500	0.523	0.600	0.500	0.500	0.500	0.500	0.500	0.484	0.603	0.510	0.500	0.500	0.500	0.500	0.500	0.472	0.500	0.500	0.500
Extra Trees	0.500	0.500	0.500	0.684	0.500	0.546	0.781	0.557	0.500	0.500	0.694	0.591	0.531	0.693	0.561	0.500	0.656	0.719	0.615	0.925	0.667	0.526	0.786	0.600	0.816	0.500	0.810	0.500	0.500	0.730
Gaussian Naive Bayes	0.813	0.483	0.583	0.678	0.652	0.560	0.754	0.565	0.649	0.814	0.694	0.871	0.781	0.778	0.481	0.648	0.780	0.741	0.668	0.853	0.712	0.770	0.800	0.709	0.816	0.744	0.850	0.710	0.632	0.730
Gradient Boosting	0.844	0.583	0.487	0.946	0.801	0.780	0.867	0.945	0.909	0.892	0.685	0.916	0.757	0.856	0.659	0.812	0.945	0.913	0.822	0.975	0.918	0.756	0.742	0.832	0.861	0.881	0.874	0.958	0.890	0.946
**Helstrom Quantum Classifier**	0.681	0.683	0.739	0.728	0.798	0.653	0.784	0.826	0.764	0.674	0.728	0.813	0.859	0.727	0.540	0.833	0.914	0.804	0.795	0.946	0.773	0.727	0.776	0.788	0.816	0.844	0.850	0.795	0.859	0.730
Linear Discriminant Analysis	0.813	0.483	0.500	0.652	0.566	0.615	0.754	0.558	0.500	0.681	0.669	0.650	0.882	0.677	0.481	0.576	0.741	0.784	0.553	0.882	0.505	0.640	0.756	0.686	0.816	0.744	0.850	0.500	0.577	0.703
Logistic Regression	0.831	0.483	0.500	0.652	0.566	0.583	0.754	0.558	0.500	0.681	0.669	0.650	0.875	0.655	0.481	0.450	0.741	0.784	0.553	0.912	0.505	0.640	0.778	0.686	0.837	0.763	0.850	0.500	0.570	0.703
Multi Layer Perceptron	0.713	0.615	0.769	0.756	0.861	0.835	0.811	0.826	0.762	0.787	0.728	0.890	0.827	0.784	0.697	0.862	0.969	0.891	0.840	0.971	0.898	0.806	0.877	0.755	0.899	1.000	0.881	0.938	0.906	0.838
Nearest Centroid	0.739	0.672	0.524	0.675	0.614	0.598	0.782	0.606	0.633	0.756	0.694	0.710	0.859	0.748	0.421	0.690	0.756	0.806	0.585	0.882	0.550	0.567	0.778	0.753	0.753	0.770	0.850	0.425	0.670	0.703
Nearest Neighbors	0.731	0.620	0.634	0.810	0.822	0.811	0.868	0.787	0.835	0.732	0.724	0.844	0.789	0.792	0.739	0.862	0.914	0.913	0.875	0.891	0.857	0.877	0.834	0.788	0.816	0.763	0.850	0.816	0.851	0.784
Passive Aggressive Classifier	0.607	0.478	0.435	0.456	0.524	0.600	0.699	0.604	0.631	0.678	0.449	0.572	0.890	0.734	0.551	0.657	0.625	0.828	0.620	0.837	0.473	0.450	0.691	0.665	0.415	0.807	0.826	0.418	0.531	0.458
Perceptron	0.726	0.559	0.500	0.677	0.500	0.594	0.640	0.582	0.615	0.417	0.500	0.598	0.710	0.627	0.620	0.724	0.747	0.733	0.638	0.757	0.548	0.446	0.756	0.683	0.591	0.789	0.818	0.500	0.670	0.678
Quadratic Discriminant Analysis	0.681	0.502	0.583	0.816	0.673	0.512	0.782	0.692	0.742	0.811	0.690	0.787	0.882	0.713	0.540	0.736	0.882	0.848	0.787	0.971	0.568	0.669	0.713	0.642	0.795	0.776	0.850	0.798	0.789	0.703
Random Forest	0.850	0.520	0.503	0.947	0.843	0.685	0.839	0.763	0.871	0.865	0.753	0.871	0.820	0.978	0.679	0.752	0.976	0.913	0.899	1.000	0.857	0.748	0.742	0.798	0.801	0.881	0.850	0.979	0.938	0.893
SVM - linear	0.731	0.483	0.500	0.652	0.500	0.591	0.754	0.558	0.500	0.500	0.690	0.650	0.882	0.748	0.386	0.483	0.772	0.828	0.574	0.853	0.547	0.711	0.800	0.732	0.837	0.813	0.850	0.500	0.500	0.730
SVM - poly	0.676	0.589	0.852	0.810	0.822	0.749	0.756	0.818	0.817	0.785	0.594	0.871	0.851	0.877	0.718	0.812	0.976	0.834	0.896	0.891	0.795	0.776	0.792	0.855	0.899	0.794	0.850	0.917	0.882	0.782
SVM - rbf	0.750	0.483	0.500	0.652	0.566	0.591	0.754	0.558	0.500	0.500	0.690	0.689	0.875	0.748	0.481	0.483	0.772	0.863	0.668	0.882	0.545	0.668	0.800	0.653	0.857	0.763	0.826	0.500	0.500	0.730

Table 4.5 | Balanced accuracy score for 30 energy image feature datasets for cell line MDA-MD-231

Classifiers	P1	P2	P3	P4	P5	P6	P7	P8	P9	P10	P11	P12	P13	P14
AdaBoost	0.944	0.923	0.857	0.929	1.000	0.836	0.943	1.000	0.785	0.866	0.978	0.971	0.969	0.944
Bernoulli Naive Bayes	0.944	0.846	0.967	0.821	0.833	0.861	0.848	0.781	0.725	0.850	0.857	0.950	0.938	0.933
Dummy Classifier	0.500	0.620	0.500	0.500	0.656	0.513	0.339	0.519	0.500	0.500	0.354	0.451	0.500	0.517
Extra Trees	0.778	0.692	0.500	0.551	0.553	0.750	0.891	0.810	0.500	0.735	0.929	1.000	0.929	0.911
Gaussian Naive Bayes	0.944	0.885	0.840	0.893	0.944	0.889	0.899	1.000	0.839	0.816	0.929	1.000	0.969	0.944
Gradient Boosting	0.982	0.923	0.929	0.929	1.000	0.863	0.921	1.000	0.839	0.837	0.978	1.000	0.969	0.944
**Helstrom Quantum Classifier**	0.927	0.846	0.857	0.929	0.918	1.000	0.957	0.969	0.813	0.925	0.964	0.971	0.969	0.944
Linear Discriminant Analysis	0.778	0.846	0.786	0.893	0.944	0.861	0.935	0.812	0.728	0.846	0.893	0.950	0.976	0.911
Logistic Regression	0.944	0.885	0.769	0.893	1.000	0.889	0.899	1.000	0.867	0.841	0.929	1.000	0.969	0.944
Multi Layer Perceptron	0.964	0.885	0.840	0.893	1.000	0.946	0.921	1.000	0.867	0.871	0.907	1.000	0.969	0.944
Nearest Centroid	0.798	0.846	0.929	0.857	0.861	0.702	0.899	0.812	0.756	0.825	0.929	0.975	0.969	0.821
Nearest Neighbors	0.982	0.825	0.857	0.943	0.946	0.944	0.921	1.000	0.865	0.950	0.929	1.000	0.969	0.921
Passive Aggressive Classifier	0.873	0.846	0.840	0.964	0.972	0.917	0.899	0.969	0.838	0.816	0.957	0.941	0.969	0.944
Perceptron	0.909	0.846	0.933	0.929	0.944	0.833	0.935	0.938	0.868	0.841	0.929	0.941	0.976	0.944
Quadratic Discriminant Analysis	0.944	0.885	0.840	0.893	0.944	0.889	0.899	1.000	0.839	0.841	0.907	0.971	0.969	0.944
Random Forest	0.944	0.923	0.857	0.907	1.000	0.917	0.921	1.000	0.867	0.866	0.978	1.000	0.969	0.944
SVM - linear	0.944	0.885	0.769	0.893	1.000	0.889	0.899	1.000	0.839	0.866	0.907	1.000	0.969	0.944
SVM - poly	0.927	0.902	0.786	0.964	0.974	0.918	0.899	0.969	0.841	0.871	0.871	0.971	0.969	0.888
SVM - rbf	0.944	0.885	0.769	0.893	1.000	0.889	0.921	1.000	0.839	0.866	0.907	1.000	0.969	0.944

Classifiers	P15	P16	P17	P18	P19	P20	P21	P22	P23	P24	P25	P26	P27	P28	P29	P30
AdaBoost	0.864	0.938	0.981	0.946	1.000	0.972	0.976	0.921	0.938	0.943	0.975	0.955	0.947	0.855	0.964	0.958
Bernoulli Naive Bayes	0.841	0.528	0.916	0.833	0.804	0.865	0.881	0.875	0.708	0.821	0.900	0.818	0.921	0.886	0.964	0.917
Dummy Classifier	0.512	0.500	0.516	0.500	0.500	0.500	0.442	0.500	0.343	0.483	0.500	0.547	0.648	0.500	0.500	0.460
Extra Trees	0.865	0.500	0.909	0.871	0.500	0.864	0.500	0.882	0.708	0.821	0.975	0.500	0.944	0.841	0.893	0.917
Gaussian Naive Bayes	0.867	0.688	0.981	0.946	1.000	0.892	0.976	0.941	0.917	0.964	1.000	0.890	0.974	0.932	1.000	0.938
Gradient Boosting	0.864	0.920	0.981	0.929	1.000	0.918	1.000	0.921	0.958	0.943	0.975	1.000	0.947	0.888	0.957	0.920
**Helstrom Quantum Classifier**	0.814	0.983	0.981	0.927	0.935	0.946	0.938	0.946	0.958	0.964	1.000	0.923	0.974	0.955	0.964	0.938
Linear Discriminant Analysis	0.867	0.500	0.916	0.778	0.935	0.892	0.945	0.846	0.917	0.857	0.900	0.773	0.867	0.864	0.893	0.896
Logistic Regression	0.893	0.858	0.981	0.929	1.000	0.893	0.945	0.971	0.958	0.964	0.975	1.000	0.974	0.888	0.978	0.938
Multi Layer Perceptron	0.867	1.000	0.981	0.927	0.978	0.972	0.969	0.946	0.958	0.964	0.975	1.000	0.974	0.798	0.978	0.958
Nearest Centroid	0.814	0.862	0.942	0.889	0.870	0.838	0.905	0.846	0.917	0.857	0.975	0.806	0.921	0.886	0.893	0.801
Nearest Neighbors	0.788	0.983	0.981	0.909	0.957	0.946	0.969	0.971	0.958	0.964	1.000	0.962	0.946	0.955	0.964	0.958
Passive Aggressive Classifier	0.839	0.692	0.890	0.893	0.964	0.893	0.945	0.971	0.917	0.921	0.975	0.909	0.974	0.855	0.943	0.885
Perceptron	0.865	0.726	0.935	0.893	0.957	0.918	0.945	0.941	0.875	0.964	0.975	0.923	0.974	0.855	0.964	0.938
Quadratic Discriminant Analysis	0.841	0.688	0.981	0.946	1.000	0.892	0.976	0.941	0.958	0.964	1.000	0.935	0.974	0.932	1.000	0.958
Random Forest	0.918	0.938	0.981	0.946	1.000	0.972	0.945	0.971	0.938	0.943	0.975	0.909	0.974	0.855	1.000	0.958
SVM - linear	0.920	0.858	0.981	0.946	0.964	0.920	0.945	0.921	0.958	0.964	0.975	0.955	0.974	0.888	0.964	0.938
SVM - poly	0.867	0.983	0.897	0.909	1.000	0.920	0.969	0.946	0.958	0.964	1.000	0.935	0.947	0.855	0.943	0.938
SVM - rbf	0.920	0.858	0.981	0.946	0.957	0.920	0.976	0.921	0.958	0.964	0.975	0.955	0.974	0.888	0.964	0.938

Table 4.6 | Balanced accuracy score for 30 homogeneity image feature datasets for cell line MDA-MD-231

Classifiers	P1	P2	P3	P4	P5	P6	P7	P8	P9	P10	P11	P12	P13	P14
AdaBoost	1.000	0.958	0.950	1.000	0.911	0.977	0.925	0.911	0.832	0.828	0.918	0.955	0.916	0.941
Bernoulli Naive Bayes	0.979	0.958	0.736	1.000	0.921	0.977	0.950	0.921	0.842	0.896	0.890	1.000	0.921	0.971
Dummy Classifier	0.345	0.603	0.510	0.418	0.500	0.500	0.490	0.500	0.539	0.560	0.380	0.427	0.500	0.500
Extra Trees	0.885	0.500	0.500	0.913	0.800	0.933	0.921	0.888	0.865	0.882	0.921	0.977	0.850	1.000
Gaussian Naive Bayes	1.000	1.000	0.967	1.000	0.821	0.977	0.946	0.921	0.865	0.912	0.890	0.977	0.891	0.971
Gradient Boosting	0.958	0.938	0.950	1.000	0.821	0.977	0.866	0.888	0.865	0.912	0.892	0.977	0.891	0.912
**Helstrom Quantum Classifier**	0.962	0.902	0.967	1.000	0.900	0.977	0.916	0.944	0.888	0.912	0.920	0.977	0.925	0.971
Linear Discriminant Analysis	1.000	0.923	0.983	1.000	0.821	1.000	0.946	0.909	0.865	0.912	0.890	1.000	0.862	0.971
Logistic Regression	1.000	0.920	1.000	1.000	0.833	0.977	0.946	0.898	0.865	0.912	0.890	0.977	0.921	0.971
Multi Layer Perceptron	1.000	0.917	0.983	1.000	0.877	0.977	0.891	0.955	0.888	0.887	0.500	1.000	0.921	0.912
Nearest Centroid	0.917	0.941	0.950	1.000	0.877	0.944	0.866	0.876	0.865	0.862	0.890	0.977	0.975	0.971
Nearest Neighbors	0.923	0.979	0.950	1.000	0.844	0.977	0.921	0.955	0.888	0.941	0.920	1.000	0.925	0.912
Passive Aggressive Classifier	0.798	0.979	0.950	0.964	0.833	0.977	0.946	0.909	0.865	0.891	0.895	0.955	0.950	1.000
Perceptron	0.899	0.917	0.983	1.000	0.921	0.921	0.875	0.932	0.729	0.799	0.946	0.955	0.866	0.891
Quadratic Discriminant Analysis	1.000	0.902	0.950	1.000	0.900	0.977	0.921	0.944	0.865	0.941	0.890	0.977	0.921	1.000
Random Forest	1.000	0.938	0.950	1.000	0.877	0.977	0.921	0.911	0.865	0.912	0.918	0.955	0.921	0.941
SVM - linear	1.000	0.881	0.967	1.000	0.833	0.977	0.946	0.921	0.865	0.912	0.864	0.955	0.921	1.000
SVM - poly	0.923	0.979	1.000	1.000	0.877	0.977	0.891	0.921	0.888	0.912	0.890	1.000	0.925	0.941
SVM - rbf	1.000	0.923	0.983	1.000	0.833	0.977	0.946	0.898	0.865	0.912	0.864	0.977	0.921	0.971

Classifiers	P15	P16	P17	P18	P19	P20	P21	P22	P23	P24	P25	P26	P27	P28	P29	P30
AdaBoost	0.864	0.879	0.978	0.971	1.000	0.969	0.893	0.947	0.955	0.971	1.000	0.955	0.946	0.957	0.945	1.000
Bernoulli Naive Bayes	0.947	0.862	0.921	0.971	0.942	0.969	1.000	0.947	0.955	1.000	0.944	0.865	0.946	0.978	0.976	1.000
Dummy Classifier	0.512	0.526	0.500	0.578	0.353	0.500	0.425	0.500	0.500	0.500	0.495	0.518	0.500	0.500	0.500	0.384
Extra Trees	0.920	0.500	0.929	0.941	0.818	1.000	0.679	0.946	1.000	0.941	0.967	0.733	0.835	0.870	0.812	0.794
Gaussian Naive Bayes	0.947	0.895	1.000	0.971	1.000	1.000	0.964	0.946	0.955	1.000	0.944	0.977	0.974	0.957	0.945	1.000
Gradient Boosting	0.863	0.824	0.978	0.971	1.000	0.969	0.929	0.947	0.955	0.971	1.000	0.977	0.974	0.921	0.914	0.941
**Helstrom Quantum Classifier**	0.921	0.895	0.978	1.000	1.000	1.000	1.000	0.974	0.955	1.000	1.000	0.977	0.974	0.957	1.000	0.971
Linear Discriminant Analysis	0.947	0.895	1.000	0.971	1.000	1.000	1.000	0.947	0.955	1.000	1.000	0.955	0.974	0.978	0.945	1.000
Logistic Regression	0.920	0.879	1.000	0.971	1.000	1.000	0.964	0.947	0.932	1.000	0.967	0.977	0.974	0.957	0.945	1.000
Multi Layer Perceptron	0.947	0.912	1.000	0.971	1.000	1.000	0.964	0.947	0.898	1.000	0.967	0.977	1.000	0.978	0.945	0.971
Nearest Centroid	0.974	0.883	0.978	0.941	1.000	1.000	0.964	0.920	1.000	0.916	0.944	0.955	0.946	0.978	0.976	0.946
Nearest Neighbors	0.893	0.912	1.000	0.946	1.000	1.000	1.000	0.974	0.921	1.000	0.944	0.977	0.974	0.978	0.969	0.971
Passive Aggressive Classifier	0.918	0.895	1.000	0.941	0.942	1.000	0.821	0.920	0.932	1.000	0.944	0.977	0.974	0.957	0.976	0.946
Perceptron	0.836	0.879	0.978	0.828	0.955	0.976	0.929	0.946	0.955	0.971	0.886	0.933	0.946	0.891	0.976	0.975
Quadratic Discriminant Analysis	0.947	0.895	1.000	0.971	1.000	1.000	1.000	0.947	0.932	1.000	0.967	0.955	0.974	0.957	0.976	1.000
Random Forest	0.920	0.912	0.978	0.971	1.000	1.000	0.929	0.947	0.955	1.000	0.967	0.977	0.974	0.957	0.945	1.000
SVM - linear	0.974	0.879	1.000	0.971	0.955	1.000	0.929	0.947	0.932	1.000	0.967	0.977	0.974	0.957	0.976	1.000
SVM - poly	0.893	0.879	1.000	0.971	1.000	1.000	0.964	0.947	0.932	1.000	0.967	0.955	0.974	0.957	1.000	0.941
SVM - rbf	0.920	0.879	1.000	0.941	1.000	1.000	0.964	0.920	0.932	1.000	0.967	0.977	0.974	0.957	0.945	1.000

Table 4.7 | AUROC score for 30 RGB image feature datasets for cell line MDA-MD-231

Classifiers	P1	P2	P3	P4	P5	P6	P7	P8	P9	P10	P11	P12	P13	P14
AdaBoost	0.855	0.804	0.618	0.882	0.917	0.764	0.882	0.906	0.885	0.789	0.865	0.885	0.858	0.925
Bernoulli Naive Bayes	0.738	0.756	0.755	0.897	0.889	0.720	0.850	0.906	0.857	0.741	0.814	0.891	0.916	0.866
Dummy Classifier	0.500	0.500	0.470	0.500	0.500	0.500	0.598	0.500	0.500	0.500	0.500	0.500	0.463	0.526
Extra Trees	0.855	0.500	0.500	0.921	0.917	0.765	0.882	0.938	0.893	0.812	0.865	0.935	0.818	0.866
Gaussian Naive Bayes	0.795	0.816	0.804	0.897	0.917	0.742	0.850	0.938	0.821	0.789	0.787	0.891	0.955	0.871
Gradient Boosting	0.897	0.686	0.769	0.851	0.890	0.776	0.938	1.000	0.907	0.827	0.811	0.871	0.935	0.896
**Helstrom Quantum Classifier**	0.857	0.837	0.618	0.795	0.917	0.727	0.890	0.938	0.964	0.851	0.814	0.885	0.916	0.950
Linear Discriminant Analysis	0.837	0.857	0.734	0.842	0.917	0.764	0.866	0.938	0.835	0.781	0.789	0.870	0.916	0.900
Logistic Regression	0.857	0.920	0.651	0.890	0.917	0.753	0.882	0.938	0.871	0.757	0.839	0.899	0.916	0.896
Multi Layer Perceptron	0.857	0.784	0.801	0.897	0.917	0.776	0.882	0.938	0.885	0.859	0.787	0.907	0.962	0.921
Nearest Neighbors	0.857	0.668	0.702	0.890	0.917	0.832	0.859	0.976	0.929	0.844	0.867	0.849	0.962	0.921
Quadratic Discriminant Analysis	0.877	0.760	0.884	0.890	0.889	0.752	0.921	0.914	0.835	0.812	0.893	0.863	0.916	0.896
Random Forest	0.855	0.784	0.785	0.882	0.917	0.720	0.969	0.945	0.871	0.796	0.839	0.907	0.935	0.925
SVM - linear	0.857	0.798	0.884	0.897	0.917	0.798	0.859	0.938	0.835	0.757	0.760	0.877	0.916	0.925
SVM - poly	0.835	0.784	0.817	0.866	0.917	0.786	0.914	0.938	0.893	0.820	0.814	0.893	0.916	0.921
SVM - rbf	0.857	0.857	0.884	0.695	0.500	0.798	0.914	0.938	0.857	0.789	0.787	0.899	0.916	0.925

Classifiers	P15	P16	P17	P18	P19	P20	P21	P22	P23	P24	P25	P26	P27	P28	P29	P30
AdaBoost	0.756	0.938	0.946	0.923	0.875	0.971	0.943	0.979	0.890	0.892	0.863	0.931	0.796	0.885	0.980	0.803
Bernoulli Naive Bayes	0.778	0.878	0.946	0.865	0.761	0.875	0.828	0.843	0.904	0.863	0.807	0.894	0.789	0.856	0.878	0.871
Dummy Classifier	0.500	0.485	0.510	0.463	0.500	0.500	0.500	0.500	0.477	0.500	0.500	0.500	0.558	0.500	0.548	0.544
Extra Trees	0.764	0.542	0.941	0.878	0.688	0.900	0.821	0.846	0.890	0.813	0.784	0.500	0.820	0.907	0.938	0.896
Gaussian Naive Bayes	0.742	0.938	0.946	0.904	0.795	0.900	0.750	0.864	0.890	0.917	0.807	0.913	0.789	0.856	0.918	0.846
Gradient Boosting	0.734	0.938	0.921	0.897	0.858	0.891	0.964	1.000	0.890	0.920	0.836	0.950	0.820	0.907	0.980	0.837
**Helstrom Quantum Classifier**	0.756	0.980	0.946	0.904	0.875	0.950	0.857	0.979	0.962	0.833	0.863	0.931	0.812	0.899	0.980	0.896
Linear Discriminant Analysis	0.778	0.897	0.971	0.885	0.688	0.850	0.835	0.938	0.825	0.833	0.756	0.881	0.812	0.899	0.940	0.866
Logistic Regression	0.778	0.938	0.946	0.885	0.795	0.900	0.792	0.979	0.890	0.917	0.836	0.931	0.820	0.907	0.980	0.891
Multi Layer Perceptron	0.863	0.960	0.921	0.923	0.875	0.896	0.871	1.000	0.909	0.920	0.889	0.913	0.789	0.907	0.980	0.891
Nearest Neighbors	0.800	0.938	0.946	0.942	0.875	0.921	0.857	0.958	0.935	0.892	0.863	0.881	0.851	0.907	0.980	0.866
Quadratic Discriminant Analysis	0.778	0.960	0.971	0.904	0.750	0.841	0.814	0.979	0.935	0.838	0.808	0.913	0.882	0.899	0.980	0.807
Random Forest	0.734	0.938	0.946	0.923	0.875	0.866	0.893	1.000	0.890	0.917	0.863	0.913	0.796	0.907	0.980	0.866
SVM - linear	0.778	0.938	0.971	0.904	0.795	0.900	0.835	0.979	0.890	0.917	0.863	0.963	0.820	0.907	0.980	0.921
SVM - poly	0.778	0.938	0.946	0.897	0.812	0.866	0.842	1.000	0.935	0.944	0.890	0.863	0.851	0.849	0.980	0.896
SVM - rbf	0.778	0.938	0.971	0.500	0.795	0.800	0.835	0.979	0.890	0.917	0.836	0.963	0.789	0.907	0.980	0.871

Results exclude Nearest Centroid, Passive Aggressive Classifier and Perceptron.

Table 4.8 | AUROC score for 30 L*u*v* image feature datasets for cell line MDA-MD-231

Classifiers	P1	P2	P3	P4	P5	P6	P7	P8	P9	P10	P11	P12	P13	P14
AdaBoost	0.825	0.851	0.838	0.943	0.890	0.804	0.895	0.775	0.866	0.878	0.920	0.967	0.795	0.906
Bernoulli Naive Bayes	0.904	0.866	0.844	0.907	0.906	0.804	0.784	0.750	0.701	0.712	0.918	0.944	0.692	0.859
Dummy Classifier	0.353	0.500	0.500	0.500	0.560	0.407	0.500	0.500	0.473	0.485	0.500	0.500	0.503	0.400
Extra Trees	0.799	0.632	0.500	0.943	0.906	0.784	0.836	0.771	0.859	0.500	0.918	0.967	0.762	0.929
Gaussian Naive Bayes	0.897	0.796	0.775	0.929	0.938	0.801	0.865	0.796	0.820	0.732	0.918	0.967	0.707	0.882
Gradient Boosting	0.780	0.749	0.838	0.943	0.914	0.766	0.921	0.825	0.890	0.815	0.867	0.921	0.795	0.835
**Helstrom Quantum Classifier**	0.844	0.921	0.838	0.943	0.938	0.822	0.892	0.771	0.890	0.772	0.918	0.967	0.756	0.882
Linear Discriminant Analysis	0.890	0.890	0.791	0.907	0.906	0.819	0.865	0.750	0.804	0.752	0.917	0.967	0.740	0.906
Logistic Regression	0.844	0.882	0.938	0.943	0.938	0.804	0.838	0.796	0.811	0.692	0.918	0.967	0.708	0.906
Multi Layer Perceptron	0.890	0.882	0.822	0.907	0.969	0.804	0.892	0.796	0.897	0.898	0.946	0.911	0.811	0.851
Nearest Neighbors	0.890	0.882	0.822	0.899	0.938	0.745	0.864	0.800	0.859	0.813	0.944	0.967	0.802	0.882
Quadratic Discriminant Analysis	0.935	0.757	0.922	0.943	0.906	0.804	0.839	0.825	0.859	0.732	0.918	0.967	0.802	0.906
Random Forest	0.825	0.851	0.853	0.907	0.938	0.784	0.895	0.796	0.842	0.795	0.946	0.944	0.732	0.859
SVM - linear	0.890	0.890	0.822	0.907	0.906	0.804	0.839	0.771	0.835	0.753	0.944	0.967	0.723	0.906
SVM - poly	0.799	0.827	0.838	0.943	0.945	0.843	0.864	0.796	0.827	0.775	0.944	0.933	0.676	0.835
SVM - rbf	0.890	0.921	0.822	0.929	0.906	0.825	0.867	0.771	0.780	0.733	0.722	0.967	0.762	0.906

Classifiers	P15	P16	P17	P18	P19	P20	P21	P22	P23	P24	P25	P26	P27	P28	P29	P30
AdaBoost	0.816	0.643	0.898	0.941	0.871	0.921	0.864	0.921	0.964	0.890	0.952	0.929	0.947	0.885	0.897	0.947
Bernoulli Naive Bayes	0.741	0.824	0.932	0.878	0.842	0.941	0.727	0.921	0.899	0.938	0.881	0.871	0.946	0.863	0.918	0.946
Dummy Classifier	0.500	0.500	0.500	0.500	0.500	0.500	0.500	0.500	0.705	0.500	0.464	0.685	0.462	0.604	0.485	0.355
Extra Trees	0.850	0.500	0.909	0.979	0.814	0.946	0.778	0.895	0.957	0.914	0.905	0.500	0.974	0.500	0.917	0.920
Gaussian Naive Bayes	0.821	0.681	0.932	0.958	0.885	0.941	0.781	0.895	0.957	0.938	0.976	0.821	1.000	0.899	0.958	0.947
Gradient Boosting	0.791	0.664	0.832	1.000	0.849	0.891	0.782	0.921	0.964	0.914	0.976	0.871	0.947	0.921	0.938	0.921
**Helstrom Quantum Classifier**	0.707	0.862	0.909	0.941	0.885	0.916	0.807	0.921	0.921	0.938	0.929	0.857	0.946	0.921	0.918	0.974
Linear Discriminant Analysis	0.821	0.626	0.909	0.938	0.849	0.921	0.753	0.895	0.935	0.906	0.929	0.821	0.946	0.891	0.938	0.974
Logistic Regression	0.821	0.626	0.932	0.958	0.798	0.946	0.781	0.921	0.957	0.938	0.921	0.893	0.946	0.863	0.958	0.974
Multi Layer Perceptron	0.796	0.752	0.921	0.902	0.885	0.866	0.838	0.895	0.964	0.914	0.952	0.964	0.946	0.929	0.938	0.946
Nearest Neighbors	0.766	0.714	0.898	0.941	0.863	0.837	0.835	0.921	0.921	0.914	0.929	0.929	0.974	0.863	0.918	0.974
Quadratic Discriminant Analysis	0.762	0.698	0.932	0.920	0.863	0.891	0.781	0.895	0.978	0.914	0.952	0.857	0.947	0.899	0.938	0.947
Random Forest	0.787	0.698	0.898	0.979	0.885	0.921	0.754	0.921	1.000	0.945	0.976	0.871	0.947	0.899	0.938	0.947
SVM - linear	0.821	0.698	0.932	0.958	0.842	0.946	0.779	0.921	0.913	0.938	0.952	0.857	0.972	0.877	0.938	0.974
SVM - poly	0.821	0.643	0.898	0.787	0.863	0.946	0.838	0.868	0.863	0.969	0.976	0.821	0.972	0.885	0.938	0.974
SVM - rbf	0.771	0.698	0.932	0.938	0.842	0.946	0.500	0.895	0.935	0.938	0.952	0.857	0.972	0.899	0.938	0.974

Results exclude Nearest Centroid, Passive Aggressive Classifier and Perceptron.

Table 4.9 | AUROC score for 30 contrast image feature datasets for cell line MDA-MD-231

Classifiers	P1	P2	P3	P4	P5	P6	P7	P8	P9	P10	P11	P12	P13	P14	P15	P16	P17	P18	P19	P20	P21	P22	P23	P24	P25	P26	P27	P28	P29	P30
AdaBoost	0.835	0.823	0.879	0.918	0.866	0.744	0.929	0.820	0.945	0.943	0.881	0.946	0.863	0.890	0.784	0.800	0.933	0.921	0.909	0.974	0.972	0.975	0.878	0.891	0.955	0.853	1.000	0.874	0.969	0.967
Bernoulli Naive Bayes	0.817	0.657	0.500	0.917	0.765	0.846	0.778	0.888	0.812	0.943	0.882	0.875	0.890	0.839	0.739	0.869	0.933	0.929	0.909	0.918	0.890	0.850	0.784	0.771	0.944	0.824	0.925	0.921	0.699	0.886
Dummy Classifier	0.500	0.500	0.438	0.431	0.500	0.500	0.490	0.500	0.500	0.526	0.500	0.515	0.500	0.567	0.481	0.500	0.373	0.464	0.360	0.563	0.386	0.500	0.500	0.500	0.500	0.500	0.494	0.473	0.473	0.500
Extra Trees	0.500	0.500	0.500	0.917	0.500	0.559	0.938	0.767	0.781	0.679	0.500	0.774	0.500	0.811	0.697	0.500	0.500	0.796	0.500	0.806	0.500	0.724	0.731	0.696	0.867	0.500	1.000	0.762	0.594	0.932
Gaussian Naive Bayes	0.817	0.886	0.862	0.918	0.789	0.782	0.881	0.888	0.875	0.821	0.914	0.975	0.890	0.839	0.878	0.800	0.933	0.945	0.864	0.946	0.841	0.950	0.840	0.796	0.977	0.824	1.000	0.897	1.000	0.955
Gradient Boosting	0.891	0.823	0.879	0.918	0.938	0.719	0.929	0.865	0.897	0.978	0.905	0.946	0.836	0.892	0.819	0.784	0.967	0.890	0.909	0.974	1.000	0.921	0.899	0.866	0.977	0.857	0.941	0.850	1.000	1.000
**Helstrom Quantum Classifier**	0.911	0.765	0.912	0.944	0.820	0.816	0.881	0.911	0.906	0.929	0.952	0.950	0.836	0.839	0.864	0.784	0.833	0.952	0.909	0.972	0.972	0.896	0.920	0.921	0.967	0.857	1.000	0.945	1.000	0.977
Linear Discriminant Analysis	0.800	0.795	0.643	0.917	0.695	0.807	0.802	0.855	0.844	0.857	0.882	0.900	0.863	0.865	0.801	0.800	0.867	0.842	0.773	0.972	0.762	0.875	0.724	0.771	0.967	0.794	0.950	0.859	0.905	0.909
Logistic Regression	0.873	0.858	0.895	0.890	0.789	0.715	0.787	0.888	0.875	0.907	0.952	0.975	0.890	0.839	0.843	0.800	0.900	0.945	0.818	0.972	0.893	0.950	0.843	0.821	0.977	0.824	0.975	0.929	1.000	0.955
Multi Layer Perceptron	0.891	0.886	0.845	0.890	0.859	0.715	0.897	0.888	0.938	0.964	0.929	0.921	0.836	0.785	0.804	0.800	0.933	0.945	0.909	0.972	0.920	0.950	0.920	0.821	0.944	0.853	0.975	0.857	1.000	0.977
Nearest Neighbors	0.873	0.789	0.895	0.944	0.749	0.782	0.905	0.921	0.906	0.964	0.952	0.946	0.836	0.813	0.846	0.800	0.933	0.929	0.909	0.972	0.946	0.925	0.920	0.921	0.977	0.853	1.000	0.952	0.952	0.955
Quadratic Discriminant Analysis	0.817	0.806	0.719	0.918	0.757	0.837	0.826	0.877	0.851	0.964	0.882	0.946	0.864	0.813	0.798	0.800	0.933	0.945	0.909	0.972	0.867	0.950	0.857	0.746	0.977	0.794	1.000	0.929	0.976	0.955
Random Forest	0.855	0.823	0.895	0.918	0.882	0.799	0.857	0.911	0.969	1.000	0.952	0.975	0.861	0.865	0.804	0.800	0.933	0.945	0.909	0.974	0.972	0.950	0.941	0.896	0.977	0.853	1.000	0.881	1.000	0.977
SVM - linear	0.835	0.823	0.879	0.890	0.765	0.740	0.787	0.888	0.844	0.964	0.929	0.975	0.890	0.865	0.819	0.800	0.900	0.969	0.818	0.972	0.890	0.950	0.825	0.796	0.977	0.794	1.000	0.929	0.976	0.932
SVM - poly	0.835	0.823	0.845	0.917	0.906	0.807	0.914	0.888	0.938	0.943	0.952	0.950	0.836	0.785	0.804	0.800	0.933	0.921	0.909	0.944	0.974	0.900	0.920	0.821	0.977	0.794	1.000	0.905	1.000	0.955
SVM - rbf	0.817	0.823	0.879	0.917	0.796	0.749	0.787	0.888	0.500	0.943	0.952	0.975	0.890	0.839	0.784	0.500	0.500	0.945	0.773	0.500	0.918	0.900	0.864	0.846	0.977	0.794	1.000	0.929	1.000	0.955

Results exclude Nearest Centroid, Passive Aggressive Classifier and Perceptron.

Table 4.10 | AUROC score for 30 correlation image feature datasets for cell line MDA-MD-231

Classifiers	P1	P2	P3	P4	P5	P6	P7	P8	P9	P10	P11	P12	P13	P14	P15	P16	P17	P18	P19	P20	P21	P22	P23	P24	P25	P26	P27	P28	P29	P30
AdaBoost	0.757	0.583	0.535	0.728	0.801	0.466	0.839	0.589	0.853	0.757	0.565	0.890	0.820	0.863	0.466	0.664	0.897	0.691	0.833	1.000	0.835	0.770	0.750	0.598	0.745	0.863	0.826	0.731	0.717	0.730
Bernoulli Naive Bayes	0.683	0.583	0.500	0.623	0.500	0.467	0.781	0.455	0.502	0.708	0.719	0.586	0.734	0.756	0.421	0.500	0.740	0.784	0.595	0.803	0.425	0.503	0.713	0.674	0.721	0.776	0.818	0.458	0.656	0.730
Dummy Classifier	0.500	0.346	0.519	0.408	0.502	0.500	0.491	0.567	0.500	0.463	0.500	0.580	0.500	0.425	0.500	0.598	0.500	0.612	0.575	0.500	0.500	0.500	0.500	0.500	0.285	0.500	0.440	0.627	0.500	0.461
Extra Trees	0.500	0.500	0.500	0.681	0.500	0.522	0.725	0.557	0.500	0.500	0.626	0.636	0.500	0.607	0.603	0.500	0.664	0.790	0.577	1.000	0.542	0.649	0.750	0.677	0.795	0.500	0.857	0.500	0.500	0.700
Gaussian Naive Bayes	0.813	0.483	0.583	0.678	0.652	0.560	0.754	0.565	0.649	0.814	0.694	0.871	0.781	0.778	0.481	0.648	0.780	0.741	0.668	0.853	0.712	0.770	0.800	0.709	0.816	0.744	0.850	0.710	0.632	0.730
Gradient Boosting	0.850	0.626	0.519	0.946	0.804	0.756	0.867	0.945	0.909	0.892	0.631	0.851	0.789	0.820	0.659	0.719	0.976	0.877	0.840	0.975	0.918	0.756	0.720	0.911	0.843	0.881	0.874	0.958	0.952	0.946
**Helstrom Quantum Classifier**	0.781	0.633	0.734	0.813	0.819	0.653	0.784	0.778	0.764	0.674	0.649	0.858	0.851	0.727	0.620	0.867	0.914	0.804	0.816	0.946	0.753	0.741	0.820	0.765	0.795	0.844	0.881	0.819	0.914	0.784
Linear Discriminant Analysis	0.813	0.483	0.500	0.652	0.566	0.615	0.754	0.558	0.500	0.681	0.669	0.650	0.882	0.677	0.481	0.576	0.741	0.784	0.553	0.882	0.505	0.640	0.756	0.686	0.816	0.744	0.850	0.500	0.577	0.703
Logistic Regression	0.831	0.483	0.500	0.652	0.545	0.615	0.754	0.558	0.500	0.681	0.669	0.650	0.906	0.655	0.481	0.433	0.741	0.784	0.553	0.971	0.505	0.640	0.778	0.686	0.837	0.763	0.850	0.500	0.570	0.703
Multi Layer Perceptron	0.763	0.633	0.785	0.838	0.745	0.747	0.784	0.881	0.500	0.709	0.585	0.871	0.820	0.828	0.739	0.790	0.741	0.913	0.861	1.000	0.940	0.733	0.806	0.877	0.899	0.844	0.850	0.917	0.906	0.807
Nearest Neighbors	0.781	0.620	0.685	0.787	0.784	0.747	0.728	0.850	0.835	0.785	0.757	0.844	0.882	0.792	0.620	0.845	0.914	0.913	0.875	0.891	0.775	0.877	0.770	0.788	0.816	0.763	0.850	0.784	0.906	0.732
Quadratic Discriminant Analysis	0.681	0.502	0.583	0.816	0.673	0.512	0.782	0.692	0.742	0.811	0.690	0.787	0.882	0.713	0.540	0.736	0.882	0.848	0.787	0.971	0.568	0.669	0.713	0.642	0.795	0.776	0.850	0.798	0.789	0.703
Random Forest	0.850	0.589	0.535	0.920	0.843	0.708	0.867	0.795	0.853	0.838	0.635	0.851	0.789	0.885	0.679	0.610	0.969	0.935	0.920	1.000	0.815	0.699	0.742	0.844	0.819	0.881	0.850	0.958	0.969	0.865
SVM - linear	0.750	0.502	0.500	0.652	0.500	0.591	0.782	0.558	0.500	0.500	0.690	0.605	0.875	0.748	0.442	0.500	0.710	0.828	0.500	0.853	0.547	0.689	0.800	0.653	0.837	0.744	0.850	0.500	0.500	0.730
SVM - poly	0.676	0.589	0.852	0.810	0.822	0.749	0.756	0.818	0.817	0.785	0.594	0.871	0.851	0.877	0.718	0.681	0.976	0.834	0.896	0.891	0.795	0.776	0.792	0.855	0.899	0.763	0.850	0.917	0.851	0.782
SVM - rbf	0.750	0.483	0.500	0.705	0.566	0.500	0.727	0.565	0.500	0.579	0.500	0.689	0.750	0.748	0.460	0.500	0.772	0.863	0.668	0.853	0.545	0.668	0.800	0.653	0.857	0.813	0.826	0.500	0.500	0.730

Results exclude Nearest Centroid, Passive Aggressive Classifier and Perceptron.

Table 4.11 | AUROC score for 30 energy image feature datasets for cell line MDA-MD-231

Classifiers	P1	P2	P3	P4	P5	P6	P7	P8	P9	P10	P11	P12	P13	P14	P15	P16	P17	P18	P19	P20	P21	P22	P23	P24	P25	P26	P27	P28	P29	P30
AdaBoost	0.871	0.885	0.857	0.929	1.000	0.836	0.943	1.000	0.788	0.866	0.978	0.971	0.969	0.944	0.890	0.920	0.935	0.929	1.000	0.972	0.976	0.946	0.980	0.943	0.975	0.909	0.947	0.888	0.964	0.958
Bernoulli Naive Bayes	0.944	0.846	0.967	0.821	0.833	0.861	0.848	0.781	0.725	0.850	0.857	0.950	0.938	0.933	0.841	0.528	0.916	0.833	0.804	0.865	0.881	0.875	0.708	0.821	0.900	0.818	0.921	0.886	0.964	0.917
Dummy Classifier	0.417	0.500	0.500	0.705	0.434	0.500	0.568	0.496	0.567	0.500	0.500	0.565	0.500	0.500	0.544	0.532	0.250	0.363	0.500	0.500	0.500	0.535	0.500	0.547	0.500	0.638	0.500	0.417	0.505	0.500
Extra Trees	0.833	0.500	0.500	0.543	0.579	0.806	0.856	0.740	0.500	0.588	0.907	0.971	0.897	0.877	0.892	0.500	0.909	0.944	0.500	0.864	0.500	0.896	0.875	0.679	0.850	0.500	0.944	0.500	0.821	0.917
Gaussian Naive Bayes	0.944	0.885	0.840	0.893	0.944	0.889	0.899	1.000	0.839	0.816	0.929	1.000	0.969	0.944	0.867	0.688	0.981	0.946	1.000	0.892	0.976	0.941	0.917	0.964	1.000	0.890	0.974	0.932	1.000	0.938
Gradient Boosting	0.927	0.846	0.929	0.842	0.972	0.863	0.921	1.000	0.839	0.812	0.978	0.971	0.969	0.944	0.811	0.920	0.962	0.929	1.000	0.946	1.000	0.950	0.958	0.943	0.975	1.000	0.921	0.855	0.957	0.958
**Helstrom Quantum Classifier**	0.927	0.846	0.786	0.893	0.946	1.000	0.921	0.969	0.865	0.866	0.929	1.000	0.969	0.921	0.814	0.983	0.981	0.927	0.804	0.947	1.000	0.946	0.958	0.893	1.000	0.942	0.946	0.955	0.964	0.917
Linear Discriminant Analysis	0.778	0.846	0.786	0.893	0.944	0.861	0.935	0.812	0.728	0.846	0.893	0.950	0.976	0.911	0.867	0.500	0.916	0.778	0.935	0.892	0.945	0.846	0.917	0.857	0.900	0.773	0.867	0.864	0.893	0.896
Logistic Regression	0.944	0.885	0.786	0.893	1.000	0.889	0.921	0.906	0.867	0.841	0.929	1.000	0.969	0.944	0.893	0.858	0.981	0.929	0.957	0.893	0.945	0.971	0.958	0.893	0.975	1.000	0.974	0.898	0.978	0.938
Multi Layer Perceptron	0.909	0.885	0.857	0.893	0.972	0.889	0.943	0.969	0.813	0.921	0.907	0.971	0.969	0.944	0.867	0.983	0.981	0.909	0.957	0.972	0.938	0.946	0.958	0.943	0.975	0.935	0.974	0.888	0.943	0.938
Nearest Neighbors	0.964	0.825	0.857	0.871	0.946	0.944	0.921	0.969	0.865	0.950	0.929	1.000	0.938	0.921	0.788	0.983	0.981	0.944	0.957	0.946	0.969	0.971	0.958	0.964	1.000	0.962	0.920	0.955	0.964	0.938
Quadratic Discriminant Analysis	0.944	0.885	0.840	0.893	0.944	0.889	0.899	1.000	0.839	0.841	0.907	0.971	0.969	0.944	0.841	0.688	0.981	0.946	1.000	0.892	0.976	0.941	0.958	0.964	1.000	0.935	0.974	0.932	1.000	0.958
Random Forest	0.927	0.923	0.857	0.964	1.000	0.917	0.943	1.000	0.867	0.866	0.943	0.971	0.969	0.944	0.865	0.938	0.981	0.946	1.000	0.946	0.945	0.941	0.958	0.943	0.975	0.955	0.974	0.888	1.000	0.958
SVM - linear	0.944	0.885	0.786	0.893	1.000	0.889	0.899	0.875	0.839	0.866	0.907	0.950	0.969	0.944	0.867	0.858	0.981	0.815	0.935	0.920	0.945	0.925	0.917	0.893	0.950	0.955	0.974	0.932	0.964	0.938
SVM - poly	0.927	0.825	0.786	0.857	0.944	0.946	0.957	0.938	0.839	0.900	0.964	1.000	0.969	0.888	0.841	0.983	0.897	0.909	0.913	0.920	0.969	0.946	0.958	0.929	0.975	0.864	0.947	0.886	0.943	0.979
SVM - rbf	0.889	0.500	0.500	0.893	1.000	0.889	0.921	1.000	0.811	0.866	0.929	0.875	0.969	0.944	0.867	0.858	0.981	0.946	0.500	0.920	0.976	0.925	0.958	0.750	0.975	0.955	0.974	0.898	0.964	0.938

Results exclude Nearest Centroid, Passive Aggressive Classifier and Perceptron.

Table 4.12 | AUROC score for 30 homogeneity image feature datasets for cell line MDA-MD-231

Classifiers	P1	P2	P3	P4	P5	P6	P7	P8	P9	P10	P11	P12	P13	P14	P15	P16	P17	P18	P19	P20	P21	P22	P23	P24	P25	P26	P27	P28	P29	P30
AdaBoost	1.000	0.920	0.950	1.000	0.911	0.977	0.891	0.911	0.832	0.882	0.918	0.955	0.916	0.941	0.838	0.840	1.000	0.971	1.000	1.000	0.929	0.947	0.955	0.971	1.000	0.977	0.974	0.957	0.945	0.975
Bernoulli Naive Bayes	0.979	0.958	0.736	1.000	0.921	0.977	0.950	0.921	0.842	0.896	0.890	1.000	0.921	0.971	0.947	0.862	0.921	0.971	0.942	0.969	1.000	0.947	0.955	1.000	0.944	0.865	0.946	0.978	0.976	1.000
Dummy Classifier	0.500	0.500	0.526	0.467	0.500	0.462	0.401	0.500	0.529	0.500	0.379	0.629	0.500	0.490	0.570	0.500	0.461	0.500	0.437	0.500	0.540	0.458	0.500	0.500	0.500	0.500	0.500	0.604	0.500	0.456
Extra Trees	0.846	0.500	0.500	1.000	0.767	0.900	0.812	0.842	0.865	0.794	0.893	1.000	0.946	0.971	0.947	0.500	1.000	0.912	0.545	1.000	0.643	0.972	1.000	0.853	0.944	0.767	0.972	0.739	0.969	0.706
Gaussian Naive Bayes	1.000	1.000	0.967	1.000	0.821	0.977	0.946	0.921	0.865	0.912	0.890	0.977	0.891	0.971	0.947	0.895	1.000	0.971	1.000	1.000	0.964	0.946	0.955	1.000	0.944	0.977	0.974	0.957	0.945	1.000
Gradient Boosting	0.958	0.938	0.950	1.000	0.944	0.977	0.891	0.888	0.865	0.853	0.918	0.955	0.891	0.912	0.865	0.895	0.978	0.971	1.000	0.945	0.929	0.947	0.955	0.971	0.967	0.955	0.974	0.899	0.914	0.941
**Helstrom Quantum Classifier**	0.962	0.979	0.950	1.000	0.900	0.977	0.946	0.911	0.774	0.916	0.920	1.000	0.891	1.000	0.947	0.967	1.000	0.971	1.000	1.000	1.000	0.974	0.955	1.000	1.000	0.955	0.946	0.957	0.976	0.853
Linear Discriminant Analysis	1.000	0.923	0.983	1.000	0.821	1.000	0.946	0.909	0.865	0.912	0.890	1.000	0.862	0.971	0.947	0.895	1.000	0.971	1.000	1.000	1.000	0.947	0.955	1.000	1.000	0.955	0.974	0.978	0.945	1.000
Logistic Regression	1.000	0.920	1.000	1.000	0.833	0.977	0.946	0.898	0.865	0.912	0.890	0.977	0.921	0.971	0.920	0.879	1.000	0.971	0.955	1.000	0.964	0.947	0.932	1.000	0.967	0.977	0.974	0.957	0.945	1.000
Multi Layer Perceptron	0.941	0.958	0.967	1.000	0.900	0.977	0.891	0.898	0.865	0.887	0.890	1.000	0.921	0.941	0.974	0.879	0.978	0.971	1.000	1.000	0.964	0.947	0.898	1.000	0.967	0.886	0.974	0.957	0.976	0.971
Nearest Neighbors	0.923	0.962	0.950	1.000	0.933	0.977	0.916	0.955	0.865	0.882	0.920	1.000	0.950	0.912	0.893	0.895	0.978	0.971	1.000	1.000	1.000	0.974	0.955	1.000	1.000	0.977	0.974	0.978	0.969	0.971
Quadratic Discriminant Analysis	1.000	0.902	0.950	1.000	0.900	0.977	0.921	0.944	0.865	0.941	0.890	0.977	0.921	1.000	0.947	0.895	1.000	0.971	1.000	1.000	1.000	0.947	0.932	1.000	0.967	0.955	0.974	0.957	0.976	1.000
Random Forest	1.000	0.938	0.950	1.000	0.877	0.977	0.921	0.911	0.865	0.882	0.892	0.955	0.921	0.941	0.920	0.840	0.978	0.971	1.000	1.000	0.929	0.947	0.955	1.000	0.967	0.977	0.974	0.957	0.945	1.000
SVM - linear	1.000	0.923	1.000	1.000	0.833	0.977	0.946	0.898	0.865	0.912	0.890	0.955	0.950	1.000	0.920	0.912	1.000	0.971	0.955	1.000	0.929	0.947	0.955	1.000	0.967	0.977	0.974	0.957	0.976	1.000
SVM - poly	0.923	0.979	1.000	1.000	0.877	0.977	0.946	0.921	0.795	0.912	0.890	0.977	0.950	0.941	0.974	0.840	1.000	0.971	1.000	0.976	0.964	0.947	0.977	0.971	0.967	0.977	0.974	0.957	0.969	0.941
SVM - rbf	1.000	0.881	0.500	1.000	0.500	0.500	0.916	0.898	0.865	0.912	0.890	0.977	0.921	0.971	0.920	0.500	0.500	0.971	0.955	1.000	0.929	0.920	0.500	1.000	0.967	0.977	0.974	0.957	0.500	1.000

Results exclude Nearest Centroid, Passive Aggressive Classifier and Perceptron.

Table 4.13 | Balanced accuracy score for 30 RGB image feature datasets for cell line U87-MG

Classifiers	P1	P2	P3	P4	P5	P6	P7	P8	P9	P10	P11	P12	P13	P14	P15	P16	P17	P18	P19	P20	P21	P22	P23	P24	P25	P26	P27	P28	P29	P30
AdaBoost	0.891	0.920	0.875	0.781	0.719	0.806	0.877	0.753	0.866	0.770	0.851	0.756	0.721	0.709	0.733	0.979	0.699	0.653	0.877	0.832	0.792	0.868	0.911	0.784	0.569	0.484	0.774	0.813	0.704	0.897
Bernoulli Naive Bayes	0.907	0.772	0.773	0.831	0.636	0.780	0.693	0.916	0.827	0.590	0.935	0.763	0.788	0.709	0.793	0.885	0.707	0.713	0.855	0.755	0.733	0.762	0.855	0.539	0.653	0.722	0.774	0.650	0.724	0.897
Dummy Classifier	0.500	0.500	0.500	0.602	0.500	0.411	0.528	0.500	0.466	0.500	0.516	0.543	0.500	0.500	0.500	0.500	0.506	0.675	0.500	0.360	0.505	0.500	0.429	0.500	0.522	0.406	0.500	0.500	0.500	0.490
Extra Trees	0.500	0.500	0.500	0.500	0.500	0.500	0.500	0.591	0.844	0.500	0.753	0.612	0.644	0.500	0.813	0.808	0.500	0.653	0.813	0.500	0.500	0.684	0.667	0.542	0.500	0.500	0.500	0.500	0.556	0.844
Gaussian Naive Bayes	0.786	0.806	0.710	0.750	0.626	0.734	0.668	0.851	0.827	0.671	0.844	0.707	0.677	0.716	0.772	0.808	0.662	0.753	0.813	0.657	0.813	0.737	0.933	0.564	0.700	0.553	0.664	0.668	0.667	0.890
Gradient Boosting	0.913	0.869	0.833	0.781	0.807	0.806	0.815	0.916	0.921	0.792	0.916	0.840	0.788	0.709	0.753	0.923	0.787	0.775	0.835	0.897	0.875	0.813	0.933	0.782	0.684	0.484	0.824	0.753	0.722	0.921
**Helstrom Quantum Classifier**	0.826	0.834	0.815	0.881	0.824	0.851	0.752	0.897	0.897	0.734	0.935	0.801	0.788	0.692	0.775	0.923	0.766	0.777	0.833	0.832	0.795	0.788	0.867	0.756	0.669	0.638	0.807	0.692	0.688	0.882
Linear Discriminant Analysis	0.821	0.903	0.833	0.750	0.643	0.689	0.772	0.773	0.765	0.742	0.844	0.780	0.733	0.653	0.813	0.885	0.644	0.690	0.833	0.799	0.772	0.788	0.900	0.724	0.600	0.484	0.698	0.668	0.667	0.882
Logistic Regression	0.871	0.886	0.813	0.750	0.643	0.741	0.773	0.818	0.921	0.742	0.916	0.819	0.711	0.716	0.813	0.885	0.669	0.753	0.813	0.844	0.752	0.816	0.900	0.699	0.600	0.484	0.698	0.690	0.722	0.945
Multi Layer Perceptron	0.957	0.869	0.813	0.644	0.790	0.767	0.712	0.844	0.921	0.762	0.916	0.819	0.755	0.698	0.795	1.000	0.757	0.775	0.833	0.897	0.752	0.865	0.900	0.731	0.438	0.684	0.807	0.733	0.631	0.914
Nearest Centroid	0.921	0.772	0.773	0.844	0.652	0.696	0.653	0.871	0.897	0.626	0.962	0.784	0.720	0.709	0.795	0.885	0.707	0.713	0.813	0.781	0.835	0.706	0.877	0.461	0.700	0.553	0.686	0.610	0.669	0.850
Nearest Neighbors	0.913	0.823	0.813	0.763	0.736	0.825	0.732	0.799	0.890	0.713	0.787	0.801	0.844	0.698	0.817	0.958	0.703	0.735	0.855	0.858	0.855	0.841	0.765	0.785	0.569	0.684	0.698	0.672	0.651	0.906
Passive Aggressive Classifier	0.978	0.817	0.875	0.631	0.681	0.741	0.773	0.727	0.890	0.691	0.820	0.780	0.765	0.675	0.675	0.792	0.463	0.713	0.777	0.573	0.730	0.730	0.855	0.621	0.506	0.569	0.719	0.612	0.744	0.738
Perceptron	0.943	0.869	0.855	0.831	0.598	0.794	0.835	0.677	0.897	0.742	0.806	0.822	0.664	0.446	0.813	0.920	0.787	0.710	0.793	0.755	0.710	0.756	0.865	0.545	0.481	0.606	0.500	0.240	0.657	0.844
Quadratic Discriminant Analysis	0.907	0.851	0.875	0.731	0.643	0.767	0.793	0.871	0.835	0.742	0.890	0.763	0.711	0.636	0.793	0.885	0.746	0.712	0.833	0.844	0.855	0.787	0.911	0.725	0.484	0.569	0.769	0.668	0.760	0.844
Random Forest	0.957	0.886	0.833	0.781	0.824	0.760	0.835	0.825	0.929	0.756	0.890	0.840	0.788	0.709	0.753	1.000	0.762	0.753	0.835	0.851	0.875	0.841	0.933	0.757	0.600	0.484	0.895	0.690	0.538	0.921
SVM - linear	0.821	0.841	0.855	0.750	0.643	0.741	0.793	0.773	0.835	0.742	0.916	0.840	0.767	0.716	0.813	0.846	0.699	0.753	0.813	0.844	0.813	0.842	0.855	0.699	0.700	0.484	0.698	0.690	0.722	0.914
SVM - poly	0.921	0.869	0.855	0.639	0.752	0.832	0.813	0.825	0.866	0.756	0.774	0.819	0.744	0.716	0.732	1.000	0.757	0.818	0.833	0.825	0.857	0.759	0.933	0.810	0.538	0.584	0.840	0.630	0.651	0.938
SVM - rbf	0.893	0.841	0.875	0.731	0.643	0.741	0.813	0.773	0.897	0.756	0.916	0.840	0.744	0.716	0.813	0.923	0.707	0.733	0.813	0.844	0.813	0.842	0.855	0.699	0.600	0.484	0.698	0.690	0.722	0.882

Table 4.14 | Balanced accuracy score for 30 L*u*v* image feature datasets for cell line U87-MG

Classifiers	P1	P2	P3	P4	P5	P6	P7	P8	P9	P10	P11	P12	P13	P14	P15	P16	P17	P18	P19	P20	P21	P22	P23	P24	P25	P26	P27	P28	P29	P30
AdaBoost	0.793	0.875	0.931	0.863	0.571	0.804	0.770	0.800	0.841	0.704	0.861	0.822	0.760	0.807	0.556	0.861	0.721	0.813	0.810	0.775	0.881	0.882	0.815	0.832	0.684	0.735	0.664	0.685	0.804	0.832
Bernoulli Naive Bayes	0.710	0.830	0.863	0.826	0.769	0.692	0.835	0.826	0.846	0.769	0.902	0.801	0.599	0.803	0.685	0.902	0.655	0.895	0.728	0.753	0.826	0.756	0.946	0.729	0.853	0.735	0.761	0.635	0.881	0.832
Dummy Classifier	0.500	0.549	0.500	0.500	0.500	0.500	0.500	0.707	0.500	0.508	0.446	0.484	0.500	0.500	0.635	0.500	0.502	0.759	0.500	0.467	0.483	0.622	0.500	0.608	0.500	0.439	0.504	0.500	0.500	0.500
Extra Trees	0.500	0.500	0.500	0.500	0.500	0.538	0.571	0.500	0.825	0.500	0.787	0.710	0.500	0.676	0.529	0.846	0.697	0.816	0.645	0.667	0.550	0.789	0.778	0.638	0.500	0.500	0.500	0.500	0.500	0.799
Gaussian Naive Bayes	0.750	0.610	0.881	0.713	0.643	0.577	0.786	0.881	0.821	0.748	0.902	0.784	0.632	0.710	0.563	0.769	0.652	0.868	0.756	0.753	0.844	0.804	0.909	0.671	0.669	0.735	0.841	0.660	0.787	0.828
Gradient Boosting	0.813	0.985	0.950	0.794	0.840	0.846	0.814	0.931	0.916	0.769	0.881	0.840	0.680	0.690	0.681	0.920	0.763	0.839	0.838	0.817	0.794	0.796	0.927	0.776	0.969	0.610	0.886	0.685	0.941	0.862
**Helstrom Quantum Classifier**	0.813	0.970	0.913	0.844	0.702	0.787	0.719	0.826	0.925	0.843	0.843	0.784	0.654	0.778	0.656	0.923	0.700	0.867	0.728	0.775	0.857	0.882	0.889	0.842	0.922	0.784	0.744	0.660	0.819	0.887
Linear Discriminant Analysis	0.750	0.750	0.800	0.700	0.643	0.538	0.671	0.850	0.775	0.769	0.846	0.748	0.604	0.681	0.568	0.885	0.715	0.813	0.728	0.833	0.863	0.741	0.833	0.673	0.700	0.625	0.761	0.706	0.731	0.824
Logistic Regression	0.750	0.750	0.850	0.813	0.643	0.538	0.778	0.831	0.896	0.769	0.902	0.825	0.604	0.656	0.597	0.885	0.718	0.893	0.757	0.733	0.913	0.772	0.889	0.729	0.700	0.610	0.761	0.685	0.731	0.853
Multi Layer Perceptron	0.855	1.000	0.831	0.781	0.555	0.689	0.792	0.950	0.975	0.763	0.881	0.825	0.576	0.774	0.665	0.885	0.718	0.838	0.785	0.795	0.913	0.827	0.944	0.830	0.953	0.735	0.789	0.685	0.979	0.841
Nearest Centroid	0.690	0.845	0.863	0.826	0.790	0.654	0.871	0.844	0.875	0.704	0.843	0.801	0.678	0.832	0.656	0.846	0.620	0.921	0.673	0.753	0.794	0.787	0.853	0.695	0.669	0.735	0.737	0.660	0.861	0.837
Nearest Neighbors	0.795	0.595	0.863	0.763	0.610	0.748	0.778	0.813	0.896	0.808	0.822	0.745	0.839	0.862	0.669	0.878	0.801	0.917	0.754	0.753	0.707	0.811	0.889	0.832	0.753	0.705	0.761	0.765	0.902	0.841
Passive Aggressive Classifier	0.750	0.735	0.839	0.844	0.807	0.748	0.750	0.826	0.812	0.825	0.798	0.760	0.577	0.699	0.538	0.769	0.609	0.838	0.652	0.857	0.894	0.772	0.855	0.497	0.753	0.735	0.636	0.685	0.710	0.799
Perceptron	0.752	0.735	0.813	0.850	0.500	0.654	0.800	0.881	0.774	0.662	0.881	0.780	0.652	0.812	0.568	0.689	0.665	0.865	0.785	0.878	0.802	0.558	0.909	0.452	0.753	0.735	0.595	0.635	0.840	0.816
Quadratic Discriminant Analysis	0.750	0.735	0.831	0.694	0.571	0.577	0.786	0.813	0.821	0.787	0.902	0.861	0.604	0.735	0.538	0.787	0.635	0.867	0.838	0.753	0.863	0.710	0.927	0.694	0.600	0.750	0.726	0.685	0.808	0.857
Random Forest	0.772	0.735	0.913	0.863	0.714	0.808	0.849	0.881	0.916	0.846	0.861	0.822	0.627	0.803	0.681	0.864	0.644	0.893	0.839	0.775	0.863	0.882	0.889	0.821	0.753	0.750	0.806	0.685	0.941	0.832
SVM - linear	0.750	0.750	0.900	0.794	0.571	0.577	0.764	0.831	0.846	0.769	0.881	0.804	0.576	0.740	0.597	0.885	0.718	0.865	0.700	0.753	0.913	0.772	0.833	0.752	0.600	0.735	0.778	0.685	0.846	0.853
SVM - poly	0.835	0.860	0.800	0.763	0.610	0.748	0.835	0.863	0.828	0.668	0.784	0.784	0.575	0.916	0.606	0.843	0.756	0.917	0.730	0.775	0.876	0.749	0.778	0.764	0.753	0.610	0.789	0.685	0.843	0.749
SVM - rbf	0.750	0.750	0.850	0.844	0.571	0.615	0.786	0.831	0.846	0.731	0.881	0.825	0.576	0.740	0.593	0.885	0.718	0.893	0.757	0.753	0.913	0.772	0.944	0.729	0.684	0.625	0.778	0.685	0.846	0.828

Table 4.15 | Balanced accuracy score for 30 contrast image feature datasets for cell line U87-MG

Classifiers	P1	P2	P3	P4	P5	P6	P7	P8	P9	P10	P11	P12	P13	P14	P15	P16	P17	P18	P19	P20	P21	P22	P23	P24	P25	P26	P27	P28	P29	P30
AdaBoost	0.897	1.000	0.946	0.800	0.806	0.898	0.946	0.863	0.887	0.923	0.921	0.893	0.777	0.814	0.812	0.742	0.826	0.835	0.842	0.844	0.885	0.890	0.780	0.850	0.879	0.807	0.752	0.897	0.800	0.810
Bernoulli Naive Bayes	0.813	0.931	0.927	0.950	0.841	0.875	0.918	0.831	0.857	0.885	0.907	0.697	0.844	0.893	0.757	0.732	0.738	0.780	0.871	0.781	0.921	0.844	0.843	0.844	0.955	0.807	0.752	0.820	0.900	0.725
Dummy Classifier	0.547	0.500	0.577	0.500	0.500	0.500	0.648	0.589	0.574	0.452	0.613	0.509	0.339	0.500	0.500	0.495	0.500	0.470	0.475	0.500	0.500	0.488	0.449	0.417	0.564	0.500	0.500	0.500	0.500	0.564
Extra Trees	0.500	0.500	0.500	0.500	0.500	0.500	0.500	0.500	0.853	0.500	0.500	0.556	0.500	0.500	0.529	0.600	0.531	0.500	0.500	0.500	0.500	0.667	0.500	0.820	0.500	0.500	0.500	0.500	0.500	0.611
Gaussian Naive Bayes	0.605	1.000	0.833	0.700	0.625	0.833	0.779	0.731	0.857	0.923	0.835	0.808	0.777	0.800	0.719	0.721	0.786	0.704	0.885	0.750	0.885	0.851	0.731	0.789	0.735	0.824	0.688	0.757	0.650	0.808
Gradient Boosting	0.793	1.000	0.964	0.813	0.761	0.940	0.835	0.781	0.862	0.958	0.921	0.920	0.844	0.899	0.841	0.798	0.881	0.762	0.863	0.844	0.885	0.890	0.843	0.835	0.845	0.879	0.793	0.818	0.831	0.782
**Helstrom Quantum Classifier**	0.773	0.963	0.891	0.813	0.823	0.980	0.918	0.863	0.857	0.979	0.921	0.836	0.855	0.776	0.757	0.832	0.881	0.762	0.877	0.950	0.907	0.818	0.840	0.882	0.924	0.950	0.730	0.835	0.863	0.861
Linear Discriminant Analysis	0.563	0.850	0.833	0.650	0.625	0.792	0.724	0.750	0.857	0.846	0.764	0.722	0.744	0.764	0.660	0.721	0.787	0.704	0.834	0.700	0.764	0.725	0.692	0.820	0.735	0.824	0.647	0.695	0.600	0.806
Logistic Regression	0.730	0.950	0.889	0.650	0.608	0.875	0.918	0.781	0.857	0.941	0.871	0.864	0.777	0.800	0.782	0.765	0.881	0.724	0.899	0.800	0.885	0.890	0.731	0.811	0.735	0.824	0.688	0.851	0.650	0.808
Multi Layer Perceptron	0.835	0.950	0.964	0.781	0.841	0.940	0.890	0.831	0.857	0.941	0.907	0.892	0.877	0.849	0.812	0.809	0.787	0.835	0.877	0.781	0.849	0.818	0.861	0.882	0.970	0.879	0.710	0.835	0.763	0.808
Nearest Centroid	0.813	0.876	0.946	0.757	0.778	0.835	0.946	0.863	0.837	0.902	0.806	0.673	0.877	0.863	0.732	0.709	0.810	0.760	0.834	0.781	0.748	0.851	0.843	0.772	0.659	0.807	0.772	0.882	0.894	0.779
Nearest Neighbors	0.855	0.950	0.833	0.800	0.823	0.938	0.890	0.881	0.857	0.923	0.863	0.863	0.921	0.792	0.782	0.798	0.826	0.835	0.863	0.850	0.871	0.826	0.822	0.882	0.985	0.879	0.710	0.842	0.750	0.864
Passive Aggressive Classifier	0.793	0.900	0.819	0.600	0.733	0.958	0.861	0.752	0.857	0.748	0.849	0.893	0.898	0.728	0.619	0.765	0.750	0.780	0.834	0.750	0.814	0.802	0.692	0.612	0.860	0.824	0.628	0.820	0.700	0.808
Perceptron	0.710	1.000	0.909	0.700	0.608	0.875	0.918	0.826	0.857	0.941	0.907	0.836	0.700	0.800	0.737	0.720	0.656	0.780	0.842	0.781	0.814	0.875	0.500	0.685	0.659	0.824	0.835	0.670	0.500	0.781
Quadratic Discriminant Analysis	0.625	1.000	0.889	0.700	0.625	0.833	0.779	0.800	0.857	0.962	0.907	0.808	0.777	0.764	0.719	0.721	0.786	0.760	0.921	0.750	0.885	0.780	0.692	0.851	0.610	0.824	0.688	0.757	0.600	0.781
Random Forest	0.855	0.950	0.982	0.800	0.858	0.940	0.918	0.831	0.862	0.881	0.907	0.946	0.877	0.814	0.841	0.776	0.826	0.835	0.877	0.781	0.885	0.914	0.902	0.835	0.970	0.807	0.752	0.897	0.800	0.810
SVM - linear	0.772	0.900	0.889	0.700	0.733	0.958	0.918	0.781	0.832	0.962	0.871	0.810	0.777	0.835	0.812	0.765	0.857	0.835	0.877	0.781	0.885	0.945	0.748	0.811	0.845	0.824	0.688	0.851	0.700	0.808
SVM - poly	0.813	0.950	0.927	0.844	0.812	0.918	0.918	0.863	0.857	0.923	0.835	0.864	0.877	0.871	0.799	0.809	0.857	0.780	0.899	0.813	0.849	0.795	0.899	0.851	0.970	0.790	0.668	0.780	0.900	0.702
SVM - rbf	0.813	0.900	0.889	0.700	0.733	1.000	0.918	0.831	0.832	0.962	0.871	0.810	0.777	0.835	0.724	0.765	0.857	0.780	0.877	0.781	0.885	0.945	0.710	0.811	0.845	0.824	0.688	0.851	0.700	0.808

Table 4.16 | Balanced accuracy score for 30 correlation image feature datasets for cell line U87-MG

Classifiers	P1	P2	P3	P4	P5	P6	P7	P8	P9	P10	P11	P12	P13	P14	P15	P16	P17	P18	P19	P20	P21	P22	P23	P24	P25	P26	P27	P28	P29	P30
AdaBoost	0.750	0.794	0.744	0.806	0.760	0.772	0.700	0.769	0.842	0.710	0.670	0.744	0.721	0.753	0.565	0.716	0.557	0.814	0.754	0.734	0.726	0.732	0.898	0.631	0.483	0.638	0.677	0.878	0.734	0.724
Bernoulli Naive Bayes	0.781	0.851	0.655	0.545	0.500	0.500	0.697	0.551	0.688	0.726	0.605	0.644	0.585	0.702	0.525	0.644	0.532	0.570	0.614	0.631	0.601	0.632	0.647	0.661	0.500	0.669	0.520	0.621	0.685	0.803
Dummy Classifier	0.500	0.573	0.615	0.500	0.430	0.500	0.537	0.505	0.500	0.500	0.547	0.500	0.500	0.491	0.500	0.424	0.500	0.469	0.500	0.547	0.500	0.440	0.467	0.552	0.390	0.500	0.354	0.500	0.500	0.500
Extra Trees	0.500	0.500	0.500	0.500	0.500	0.500	0.528	0.500	0.500	0.500	0.500	0.500	0.500	0.500	0.542	0.643	0.500	0.500	0.500	0.500	0.500	0.622	0.500	0.500	0.500	0.500	0.500	0.500	0.500	0.710
Gaussian Naive Bayes	0.700	0.753	0.706	0.591	0.643	0.730	0.699	0.618	0.665	0.695	0.587	0.611	0.606	0.753	0.607	0.644	0.532	0.707	0.699	0.820	0.695	0.707	0.752	0.624	0.500	0.669	0.635	0.729	0.702	0.832
Gradient Boosting	0.800	0.864	0.800	0.670	0.832	0.813	0.753	0.952	0.842	0.616	0.773	0.867	0.686	0.702	0.798	0.810	0.757	0.828	0.702	0.825	0.781	0.728	0.918	0.725	0.545	0.653	0.820	0.755	0.750	0.749
**Helstrom Quantum Classifier**	0.913	0.897	0.857	0.657	0.671	0.817	0.781	0.852	0.809	0.686	0.737	0.855	0.579	0.725	0.697	0.914	0.699	0.720	0.785	0.762	0.765	0.653	0.920	0.677	0.640	0.644	0.725	0.736	0.753	0.724
Linear Discriminant Analysis	0.531	0.773	0.688	0.573	0.572	0.585	0.728	0.618	0.598	0.695	0.545	0.677	0.630	0.702	0.587	0.723	0.507	0.627	0.697	0.878	0.609	0.707	0.690	0.528	0.500	0.653	0.585	0.593	0.602	0.774
Logistic Regression	0.531	0.773	0.688	0.591	0.526	0.585	0.728	0.618	0.598	0.695	0.545	0.644	0.630	0.702	0.587	0.699	0.507	0.627	0.697	0.878	0.609	0.707	0.690	0.528	0.500	0.653	0.585	0.593	0.602	0.744
Multi Layer Perceptron	0.731	0.851	0.744	0.789	0.696	0.775	0.753	0.753	0.865	0.757	0.737	0.821	0.526	0.699	0.737	0.866	0.674	0.697	0.700	0.897	0.664	0.719	0.878	0.725	0.636	0.584	0.834	0.612	0.833	0.703
Nearest Centroid	0.844	0.878	0.692	0.657	0.671	0.677	0.730	0.688	0.764	0.677	0.737	0.742	0.617	0.753	0.552	0.780	0.544	0.539	0.757	0.820	0.686	0.682	0.755	0.622	0.498	0.675	0.626	0.659	0.672	0.891
Nearest Neighbors	0.813	0.780	0.762	0.761	0.696	0.837	0.781	0.801	0.820	0.702	0.857	0.877	0.603	0.838	0.713	0.874	0.619	0.762	0.677	0.839	0.686	0.753	0.898	0.756	0.744	0.638	0.806	0.626	0.868	0.703
Passive Aggressive Classifier	0.583	0.878	0.500	0.573	0.526	0.480	0.592	0.567	0.642	0.567	0.445	0.688	0.750	0.753	0.533	0.500	0.500	0.661	0.725	0.677	0.749	0.565	0.673	0.561	0.636	0.644	0.500	0.476	0.435	0.703
Perceptron	0.857	0.846	0.694	0.606	0.657	0.545	0.620	0.559	0.632	0.600	0.673	0.765	0.606	0.760	0.482	0.653	0.499	0.512	0.700	0.657	0.644	0.682	0.630	0.560	0.500	0.500	0.562	0.654	0.484	0.762
Quadratic Discriminant Analysis	0.681	0.753	0.671	0.591	0.643	0.710	0.697	0.602	0.586	0.781	0.630	0.677	0.627	0.702	0.628	0.644	0.532	0.649	0.727	0.878	0.640	0.762	0.753	0.662	0.562	0.669	0.685	0.528	0.618	0.719
Random Forest	0.800	0.844	0.706	0.608	0.806	0.855	0.753	0.968	0.876	0.695	0.753	0.811	0.647	0.727	0.797	0.833	0.728	0.770	0.697	0.916	0.757	0.703	0.918	0.765	0.562	0.684	0.756	0.787	0.801	0.782
SVM - linear	0.500	0.780	0.706	0.608	0.500	0.500	0.728	0.500	0.598	0.695	0.565	0.611	0.500	0.728	0.587	0.692	0.503	0.500	0.700	0.813	0.577	0.707	0.690	0.521	0.500	0.500	0.500	0.547	0.602	0.774
SVM - poly	0.831	0.864	0.835	0.556	0.787	0.837	0.703	0.621	0.898	0.719	0.797	0.742	0.582	0.808	0.755	0.890	0.607	0.713	0.700	0.916	0.734	0.703	0.753	0.693	0.761	0.653	0.691	0.626	0.868	0.782
SVM - rbf	0.500	0.799	0.706	0.625	0.500	0.500	0.782	0.583	0.598	0.656	0.565	0.577	0.500	0.754	0.587	0.740	0.532	0.571	0.700	0.878	0.577	0.707	0.690	0.600	0.500	0.500	0.500	0.605	0.602	0.774

Table 4.17 | Balanced accuracy score for 30 energy image feature datasets for cell line U87-MG

Classifiers	P1	P2	P3	P4	P5	P6	P7	P8	P9	P10	P11	P12	P13	P14	P15	P16	P17	P18	P19	P20	P21	P22	P23	P24	P25	P26	P27	P28	P29	P30
AdaBoost	0.927	0.940	0.984	0.852	0.752	0.920	0.977	0.760	0.979	0.772	0.863	0.933	0.778	0.920	0.938	0.849	0.856	0.875	0.933	1.000	0.835	0.877	0.820	0.878	0.833	0.971	0.818	0.878	0.700	0.921
Bernoulli Naive Bayes	0.762	0.898	0.953	0.820	0.500	0.828	0.886	0.651	0.846	0.842	0.863	0.820	0.748	0.886	0.591	0.863	0.703	0.825	0.708	0.522	0.692	0.798	0.671	0.900	0.500	0.985	0.710	0.833	0.684	0.841
Dummy Classifier	0.560	0.500	0.528	0.500	0.442	0.500	0.518	0.500	0.500	0.500	0.500	0.500	0.500	0.578	0.659	0.500	0.452	0.500	0.438	0.463	0.588	0.500	0.500	0.593	0.500	0.382	0.500	0.490	0.375	0.500
Extra Trees	0.500	0.542	0.500	0.500	0.500	0.500	0.500	0.500	0.769	0.500	0.579	0.500	0.500	0.500	0.500	0.714	0.500	0.824	0.844	0.500	0.500	0.500	0.500	0.818	0.500	0.500	0.500	0.500	0.500	0.700
Gaussian Naive Bayes	0.944	0.958	0.984	0.887	0.835	0.920	0.955	0.853	0.941	0.897	0.972	0.888	0.849	0.869	0.906	0.871	0.682	0.850	0.876	1.000	0.877	0.838	0.839	0.920	0.785	0.985	0.916	0.837	0.900	0.866
Gradient Boosting	0.871	0.940	0.900	0.952	0.752	0.938	0.977	0.669	0.923	0.796	0.918	0.933	0.800	0.920	0.922	0.828	0.834	0.812	0.921	1.000	0.855	0.772	0.806	0.878	0.868	0.985	0.851	0.881	0.800	0.946
**Helstrom Quantum Classifier**	0.964	0.898	0.984	0.871	0.835	0.966	0.967	0.891	0.962	0.882	0.944	0.932	0.913	0.886	0.906	0.871	0.812	0.896	0.921	0.980	0.920	0.815	0.858	0.880	0.836	0.956	0.851	0.857	0.900	0.916
Linear Discriminant Analysis	0.889	0.898	0.984	0.919	0.710	0.920	0.932	0.778	0.979	0.859	0.917	0.832	0.828	0.920	0.922	0.849	0.595	0.850	0.876	0.688	0.815	0.857	0.820	0.880	0.500	0.985	0.799	0.857	0.800	0.816
Logistic Regression	0.927	0.938	0.984	0.935	0.835	0.920	0.955	0.778	0.979	0.804	0.944	0.933	0.800	0.841	0.906	0.835	0.747	0.925	0.898	0.958	0.813	0.878	0.858	0.837	0.817	0.985	0.799	0.819	0.800	0.841
Multi Layer Perceptron	0.944	0.918	0.984	0.935	0.752	0.920	0.944	0.835	0.979	0.804	0.944	0.911	0.778	0.841	0.938	0.800	0.812	0.921	0.876	0.958	0.813	0.878	0.774	0.775	0.817	0.971	0.851	0.819	0.900	0.946
Nearest Centroid	0.946	0.940	0.922	0.871	0.878	0.851	0.955	0.819	0.920	0.842	0.863	0.853	0.856	0.817	0.906	0.806	0.790	0.925	0.808	0.898	0.818	0.878	0.846	0.673	0.820	0.985	0.813	0.816	0.953	0.841
Nearest Neighbors	0.944	0.918	1.000	0.785	0.710	0.920	0.911	0.891	1.000	0.827	0.944	0.955	0.814	0.841	0.922	0.828	0.747	0.891	0.865	0.960	0.897	0.813	0.916	0.898	0.852	0.971	0.844	0.822	0.984	0.971
Passive Aggressive Classifier	0.909	0.960	0.984	0.836	0.835	0.938	0.977	0.667	0.979	0.787	0.944	0.833	0.800	0.879	0.706	0.885	0.545	0.853	0.808	0.750	0.708	0.585	0.755	0.878	0.801	0.985	0.734	0.840	0.800	0.816
Perceptron	0.964	0.940	0.800	0.968	0.710	0.938	0.944	0.889	0.979	0.686	0.895	0.888	0.849	0.670	0.922	0.849	0.562	0.950	0.911	0.980	0.688	0.752	0.904	0.940	0.583	0.868	0.663	0.756	0.800	0.950
Quadratic Discriminant Analysis	0.889	0.958	0.984	0.887	0.793	0.938	0.932	0.815	0.941	0.929	0.944	0.888	0.849	0.869	0.891	0.871	0.682	0.850	0.853	1.000	0.918	0.797	0.858	0.920	0.618	1.000	0.871	0.857	0.900	0.866
Random Forest	0.944	0.960	1.000	0.952	0.752	0.938	0.977	0.722	0.979	0.796	0.947	0.933	0.800	0.920	0.984	0.849	0.812	0.950	0.967	1.000	0.833	0.813	0.916	0.877	0.833	0.985	0.799	0.881	0.800	0.950
SVM - linear	0.927	0.960	0.984	0.919	0.835	0.920	0.977	0.778	0.979	0.835	0.972	0.911	0.814	0.920	0.906	0.871	0.725	0.900	0.898	0.958	0.813	0.878	0.858	0.878	0.833	0.985	0.890	0.840	0.900	0.841
SVM - poly	0.927	0.938	0.984	0.884	0.710	0.920	0.977	0.778	0.979	0.851	0.946	0.888	0.828	0.903	0.922	0.835	0.747	0.875	0.944	0.958	0.875	0.837	0.878	0.940	0.801	0.971	0.890	0.881	0.900	0.841
SVM - rbf	0.927	0.960	0.984	0.903	0.835	0.920	0.977	0.853	0.979	0.835	0.972	0.888	0.814	0.903	0.906	0.835	0.747	0.925	0.898	0.958	0.813	0.878	0.858	0.878	0.833	0.985	0.890	0.840	0.900	0.841

Table 4.18 | Balanced accuracy score for 30 homogeneity image feature datasets for cell line U87-MG

Classifiers	P1	P2	P3	P4	P5	P6	P7	P8	P9	P10	P11	P12	P13	P14	P15	P16	P17	P18	P19	P20	P21	P22	P23	P24	P25	P26	P27	P28	P29	P30
AdaBoost	0.931	0.970	0.969	0.858	0.653	1.000	0.981	0.920	0.978	0.842	0.855	0.921	0.837	0.878	0.859	0.979	0.946	0.811	0.876	0.955	0.929	0.865	0.897	0.876	0.799	0.938	1.000	0.864	1.000	0.895
Bernoulli Naive Bayes	0.931	0.970	0.952	0.744	0.789	1.000	0.962	0.869	0.935	0.826	0.858	0.871	0.854	0.833	0.897	0.896	0.946	0.810	0.876	0.942	0.957	0.893	0.904	0.757	0.715	0.886	0.918	0.840	0.631	0.816
Dummy Classifier	0.500	0.333	0.500	0.500	0.500	0.500	0.463	0.522	0.500	0.404	0.500	0.500	0.500	0.365	0.371	0.478	0.500	0.500	0.461	0.500	0.346	0.500	0.500	0.500	0.500	0.500	0.500	0.500	0.500	0.466
Extra Trees	0.500	0.750	0.625	0.500	0.500	0.500	0.818	0.500	0.929	0.500	0.750	0.500	0.500	0.500	0.664	0.731	0.861	0.838	0.900	0.636	0.500	0.864	0.500	0.857	0.500	0.500	0.500	0.500	0.500	0.658
Gaussian Naive Bayes	0.931	0.985	0.969	0.869	0.823	1.000	0.935	0.920	0.978	0.828	0.938	0.921	0.837	0.918	0.835	0.958	0.946	0.810	0.944	0.935	0.929	0.865	0.897	0.757	0.844	0.920	0.897	0.902	1.000	0.868
Gradient Boosting	0.931	0.985	0.844	0.858	0.653	1.000	0.981	0.841	0.978	0.778	0.855	0.921	0.899	0.877	0.679	0.958	0.972	0.811	0.898	0.890	0.907	0.867	0.916	0.876	0.844	0.938	0.958	0.822	0.833	0.895
**Helstrom Quantum Classifier**	0.931	0.985	1.000	0.834	0.869	1.000	0.962	0.869	0.943	0.891	0.938	0.921	0.878	0.898	0.835	0.979	0.972	0.865	0.865	0.962	0.964	0.867	0.942	0.876	0.871	0.886	0.980	0.881	0.946	0.947
Linear Discriminant Analysis	0.931	0.985	0.969	0.869	0.698	1.000	0.890	0.903	0.957	0.863	0.918	0.921	0.857	0.918	0.827	0.979	0.946	0.838	0.911	0.897	0.929	0.865	0.897	0.757	0.844	0.920	0.917	0.902	1.000	0.895
Logistic Regression	0.931	0.985	0.969	0.903	0.636	1.000	0.890	0.920	0.978	0.842	0.855	0.921	0.881	0.918	0.827	0.958	0.946	0.838	0.888	0.935	0.893	0.865	0.897	0.807	0.753	0.938	0.917	0.864	0.944	0.868
Multi Layer Perceptron	0.931	0.985	0.969	0.823	0.636	1.000	0.916	0.920	0.978	0.863	0.938	0.921	0.861	0.918	0.827	0.941	0.946	0.839	0.888	0.916	0.929	0.893	0.897	0.839	0.871	0.938	0.917	0.840	0.927	0.921
Nearest Centroid	0.931	0.985	0.976	0.851	0.851	1.000	0.942	0.886	0.935	0.877	0.940	0.921	0.875	0.920	0.749	0.958	0.946	0.893	0.898	0.897	0.929	0.867	0.923	0.776	0.878	0.903	0.938	0.840	0.964	0.865
Nearest Neighbors	0.931	0.985	0.969	0.806	0.716	0.964	0.890	0.920	0.964	0.935	0.897	0.971	0.878	0.898	0.686	0.902	0.972	0.920	0.888	0.916	0.893	0.865	0.916	0.876	0.825	0.903	0.813	0.902	0.944	0.947
Passive Aggressive Classifier	0.931	0.985	0.969	0.869	0.653	1.000	0.916	0.920	0.921	0.842	0.918	0.921	0.804	0.878	0.765	0.958	0.946	0.759	0.911	0.916	0.857	0.788	0.864	0.794	0.799	0.858	0.918	0.878	1.000	0.895
Perceptron	0.931	0.985	0.938	0.841	0.608	0.857	0.864	0.841	0.921	0.913	0.918	0.871	0.668	0.897	0.476	0.780	0.889	0.760	0.911	0.935	0.885	0.865	0.897	0.794	0.806	0.983	0.980	0.822	1.000	0.684
Quadratic Discriminant Analysis	0.931	0.985	0.969	0.869	0.823	1.000	0.890	0.903	0.978	0.863	0.855	0.921	0.878	0.918	0.859	0.958	0.946	0.865	0.911	0.935	0.929	0.865	0.916	0.857	0.799	0.920	0.938	0.902	1.000	0.868
Random Forest	0.931	0.985	0.938	0.841	0.653	1.000	0.981	0.920	0.964	0.863	0.855	0.921	0.861	0.938	0.820	1.000	0.946	0.811	0.911	0.935	0.929	0.865	0.897	0.907	0.799	0.938	0.980	0.843	0.889	0.895
SVM - linear	0.931	0.985	0.938	0.903	0.636	0.929	0.890	0.920	0.957	0.842	0.938	0.921	0.878	0.918	0.796	0.979	0.946	0.838	0.888	0.916	0.929	0.893	0.897	0.757	0.753	0.938	0.958	0.864	0.944	0.868
SVM - poly	0.931	0.985	0.938	0.903	0.653	0.964	0.962	0.920	0.943	0.863	0.917	0.971	0.881	0.958	0.796	0.979	0.946	0.865	0.911	0.916	0.929	0.867	0.897	0.857	0.871	0.938	0.813	0.822	0.944	0.842
SVM - rbf	0.931	0.985	0.969	0.903	0.653	0.929	0.890	0.903	0.978	0.842	0.938	0.921	0.881	0.918	0.796	0.979	0.946	0.864	0.888	0.935	0.929	0.865	0.897	0.807	0.753	0.938	0.958	0.902	0.944	0.868

Table 4.19 | AUROC score for 30 RGB image feature datasets for cell line U87-MG

Classifiers	P1	P2	P3	P4	P5	P6	P7	P8	P9	P10	P11	P12	P13	P14	P15	P16	P17	P18	P19	P20	P21	P22	P23	P24	P25	P26	P27	P28	P29	P30
AdaBoost	0.863	0.920	0.833	0.731	0.752	0.806	0.793	0.844	0.890	0.693	0.916	0.777	0.721	0.584	0.733	0.979	0.522	0.633	0.875	0.799	0.708	0.868	0.900	0.784	0.700	0.484	0.807	0.813	0.649	0.897
Bernoulli Naive Bayes	0.907	0.772	0.773	0.831	0.636	0.780	0.693	0.916	0.827	0.590	0.935	0.763	0.788	0.709	0.793	0.885	0.707	0.713	0.855	0.755	0.733	0.762	0.855	0.539	0.653	0.722	0.774	0.650	0.724	0.897
Dummy Classifier	0.512	0.500	0.568	0.483	0.500	0.528	0.527	0.463	0.500	0.500	0.509	0.500	0.500	0.453	0.500	0.404	0.447	0.552	0.500	0.500	0.500	0.380	0.500	0.500	0.659	0.569	0.500	0.487	0.577	0.500
Extra Trees	0.643	0.500	0.500	0.500	0.500	0.500	0.500	0.500	0.656	0.500	0.682	0.689	0.644	0.500	0.813	0.769	0.593	0.653	0.792	0.545	0.500	0.684	0.567	0.569	0.500	0.500	0.500	0.500	0.500	0.500
Gaussian Naive Bayes	0.786	0.806	0.710	0.750	0.626	0.734	0.668	0.851	0.827	0.671	0.844	0.707	0.677	0.716	0.772	0.808	0.662	0.753	0.813	0.657	0.813	0.737	0.933	0.564	0.700	0.553	0.664	0.668	0.667	0.890
Gradient Boosting	0.913	0.869	0.792	0.844	0.790	0.806	0.817	0.818	0.921	0.770	0.871	0.798	0.811	0.761	0.773	1.000	0.812	0.693	0.835	0.851	0.875	0.867	0.933	0.808	0.700	0.484	0.895	0.753	0.722	0.859
**Helstrom Quantum Classifier**	0.935	0.903	0.877	0.831	0.769	0.760	0.752	0.935	0.890	0.734	0.844	0.822	0.644	0.698	0.797	0.962	0.741	0.797	0.813	0.851	0.708	0.762	0.867	0.756	0.700	0.484	0.769	0.733	0.688	0.914
Linear Discriminant Analysis	0.821	0.903	0.833	0.750	0.643	0.689	0.772	0.773	0.765	0.742	0.844	0.780	0.733	0.653	0.813	0.885	0.644	0.690	0.833	0.799	0.772	0.788	0.900	0.724	0.600	0.484	0.698	0.668	0.667	0.882
Logistic Regression	0.871	0.841	0.813	0.750	0.643	0.760	0.773	0.818	0.921	0.742	0.916	0.801	0.711	0.716	0.813	0.846	0.669	0.753	0.833	0.844	0.752	0.759	0.900	0.699	0.600	0.469	0.698	0.690	0.760	0.938
Multi Layer Perceptron	0.935	0.886	0.855	0.750	0.769	0.760	0.857	0.844	0.890	0.598	0.897	0.798	0.711	0.698	0.878	1.000	0.762	0.775	0.813	0.851	0.813	0.839	0.867	0.731	0.584	0.469	0.824	0.693	0.722	0.882
Nearest Neighbors	0.913	0.795	0.813	0.831	0.769	0.825	0.732	0.935	0.835	0.685	0.955	0.801	0.811	0.698	0.795	0.979	0.703	0.838	0.855	0.851	0.855	0.734	0.877	0.785	0.600	0.484	0.698	0.795	0.651	0.906
Quadratic Discriminant Analysis	0.907	0.851	0.875	0.731	0.643	0.767	0.793	0.871	0.835	0.742	0.890	0.763	0.711	0.636	0.793	0.885	0.746	0.712	0.833	0.844	0.855	0.787	0.911	0.725	0.484	0.569	0.769	0.668	0.760	0.844
Random Forest	0.935	0.886	0.833	0.781	0.824	0.760	0.835	0.825	0.929	0.792	0.890	0.840	0.788	0.709	0.773	1.000	0.791	0.753	0.835	0.897	0.813	0.867	0.933	0.782	0.600	0.484	0.824	0.753	0.593	0.914
SVM - linear	0.821	0.841	0.855	0.750	0.571	0.734	0.793	0.773	0.897	0.742	0.916	0.822	0.677	0.716	0.793	0.846	0.699	0.753	0.813	0.844	0.792	0.842	0.867	0.699	0.600	0.484	0.698	0.648	0.722	0.914
SVM - poly	0.714	0.869	0.708	0.700	0.643	0.708	0.647	0.799	0.866	0.514	0.864	0.819	0.677	0.653	0.732	1.000	0.757	0.818	0.772	0.825	0.792	0.788	0.800	0.810	0.538	0.484	0.840	0.712	0.556	0.938
SVM - rbf	0.893	0.858	0.875	0.650	0.500	0.760	0.813	0.773	0.897	0.756	0.916	0.840	0.677	0.716	0.772	0.923	0.707	0.733	0.813	0.844	0.500	0.842	0.900	0.699	0.600	0.484	0.698	0.648	0.722	0.882

Results exclude Nearest Centroid, Passive Aggressive Classifier and Perceptron.

Table 4.20 | AUROC score for 30 L*u*v* image feature datasets for cell line U87-MG

Classifiers	P1	P2	P3	P4	P5	P6	P7	P8	P9	P10	P11	P12	P13	P14	P15	P16	P17	P18	P19	P20	P21	P22	P23	P24	P25	P26	P27	P28	P29	P30
AdaBoost	0.793	0.875	0.900	0.781	0.500	0.804	0.849	0.831	0.916	0.787	0.861	0.822	0.627	0.807	0.581	0.861	0.683	0.813	0.836	0.775	0.863	0.851	0.871	0.832	0.600	0.500	0.744	0.735	0.843	0.832
Bernoulli Naive Bayes	0.710	0.830	0.863	0.826	0.769	0.692	0.835	0.826	0.846	0.769	0.902	0.801	0.599	0.803	0.685	0.902	0.655	0.895	0.728	0.753	0.826	0.756	0.946	0.729	0.853	0.735	0.761	0.635	0.881	0.832
Dummy Classifier	0.403	0.614	0.500	0.594	0.471	0.365	0.519	0.500	0.549	0.500	0.442	0.446	0.500	0.518	0.500	0.421	0.599	0.545	0.500	0.510	0.594	0.500	0.542	0.500	0.522	0.500	0.500	0.500	0.532	0.438
Extra Trees	0.500	0.500	0.500	0.500	0.500	0.500	0.500	0.500	0.825	0.500	0.748	0.808	0.500	0.588	0.500	0.808	0.596	0.789	0.645	0.500	0.500	0.749	0.500	0.673	0.500	0.500	0.500	0.500	0.500	0.765
Gaussian Naive Bayes	0.750	0.610	0.881	0.713	0.643	0.577	0.786	0.881	0.821	0.748	0.902	0.784	0.632	0.710	0.563	0.769	0.652	0.868	0.756	0.753	0.844	0.804	0.909	0.671	0.669	0.735	0.841	0.660	0.787	0.828
Gradient Boosting	0.772	0.985	0.931	0.794	0.714	0.846	0.849	0.931	0.916	0.769	0.881	0.822	0.654	0.719	0.735	0.899	0.763	0.867	0.865	0.817	0.863	0.945	0.871	0.776	0.653	0.750	0.869	0.774	0.941	0.837
**Helstrom Quantum Classifier**	0.855	0.955	0.931	0.681	0.824	0.654	0.778	0.826	0.875	0.731	0.843	0.766	0.632	0.769	0.681	0.920	0.774	0.867	0.700	0.755	0.813	0.882	0.889	0.876	0.738	0.625	0.778	0.685	0.881	0.787
Linear Discriminant Analysis	0.750	0.750	0.800	0.700	0.643	0.538	0.671	0.850	0.775	0.769	0.846	0.748	0.604	0.681	0.568	0.885	0.715	0.813	0.728	0.792	0.863	0.741	0.833	0.673	0.700	0.625	0.761	0.706	0.731	0.824
Logistic Regression	0.750	0.750	0.850	0.813	0.643	0.538	0.778	0.831	0.925	0.769	0.923	0.804	0.604	0.656	0.597	0.885	0.718	0.893	0.757	0.775	0.913	0.772	0.889	0.729	0.700	0.625	0.761	0.685	0.731	0.853
Multi Layer Perceptron	0.855	0.625	0.876	0.813	0.643	0.538	0.814	0.913	0.946	0.769	0.843	0.804	0.605	0.774	0.597	0.885	0.676	0.865	0.757	0.815	0.913	0.827	0.944	0.853	0.700	0.625	0.761	0.660	0.941	0.891
Nearest Neighbors	0.792	0.750	0.950	0.763	0.571	0.748	0.742	0.813	0.896	0.808	0.825	0.745	0.656	0.857	0.656	0.899	0.801	0.917	0.756	0.753	0.881	0.765	0.889	0.832	0.838	0.595	0.761	0.765	0.902	0.857
Quadratic Discriminant Analysis	0.750	0.735	0.831	0.694	0.571	0.577	0.786	0.813	0.821	0.787	0.902	0.861	0.604	0.735	0.538	0.787	0.635	0.867	0.838	0.753	0.863	0.710	0.927	0.694	0.600	0.750	0.726	0.685	0.808	0.857
Random Forest	0.772	0.860	0.950	0.863	0.571	0.808	0.871	0.881	0.916	0.846	0.881	0.822	0.656	0.744	0.681	0.861	0.742	0.867	0.865	0.775	0.863	0.882	0.889	0.821	0.569	0.625	0.806	0.685	0.902	0.807
SVM - linear	0.750	0.625	0.900	0.794	0.500	0.577	0.764	0.850	0.846	0.654	0.902	0.804	0.576	0.681	0.597	0.885	0.718	0.865	0.839	0.753	0.913	0.772	0.833	0.752	0.600	0.625	0.778	0.685	0.846	0.853
SVM - poly	0.835	0.860	0.900	0.763	0.500	0.538	0.750	0.750	0.925	0.654	0.864	0.784	0.605	0.735	0.504	0.846	0.753	0.865	0.784	0.775	0.781	0.882	0.778	0.764	0.700	0.625	0.789	0.618	0.692	0.749
SVM - rbf	0.750	0.500	0.850	0.844	0.571	0.615	0.786	0.850	0.775	0.577	0.881	0.825	0.632	0.681	0.593	0.846	0.697	0.893	0.813	0.753	0.913	0.772	0.778	0.729	0.684	0.500	0.778	0.685	0.808	0.853

Results exclude Nearest Centroid, Passive Aggressive Classifier and Perceptron.

Table 4.21 | AUROC score for 30 contrast image feature datasets for cell line U87-MG

Classifiers	P1	P2	P3	P4	P5	P6	P7	P8	P9	P10	P11	P12	P13	P14	P15	P16	P17	P18	P19	P20	P21	P22	P23	P24	P25	P26	P27	P28	P29	P30
AdaBoost	0.897	1.000	0.964	0.800	0.841	0.898	0.918	0.831	0.921	0.923	0.921	0.865	0.855	0.814	0.787	0.798	0.826	0.835	0.877	0.944	0.885	0.890	0.769	0.866	0.924	0.807	0.752	0.897	0.800	0.810
Bernoulli Naive Bayes	0.813	0.931	0.927	0.950	0.841	0.875	0.918	0.831	0.857	0.885	0.907	0.697	0.844	0.893	0.757	0.732	0.738	0.780	0.871	0.781	0.921	0.844	0.843	0.844	0.955	0.807	0.752	0.820	0.900	0.725
Dummy Classifier	0.500	0.444	0.542	0.557	0.379	0.500	0.500	0.500	0.431	0.529	0.500	0.484	0.541	0.576	0.500	0.500	0.591	0.579	0.500	0.500	0.534	0.500	0.567	0.472	0.614	0.505	0.500	0.615	0.396	0.500
Extra Trees	0.500	0.500	0.500	0.500	0.500	0.500	0.500	0.500	0.824	0.500	0.500	0.500	0.500	0.500	0.500	0.500	0.531	0.500	0.607	0.500	0.500	0.643	0.500	0.765	0.500	0.500	0.500	0.500	0.500	0.500
Gaussian Naive Bayes	0.605	1.000	0.833	0.700	0.625	0.833	0.779	0.731	0.857	0.923	0.835	0.808	0.777	0.800	0.719	0.721	0.786	0.704	0.885	0.750	0.885	0.851	0.731	0.789	0.735	0.824	0.688	0.757	0.650	0.808
Gradient Boosting	0.855	1.000	0.964	0.831	0.858	0.940	0.890	0.781	0.921	0.979	0.921	0.972	0.811	0.899	0.866	0.765	0.850	0.835	0.842	0.944	0.885	0.890	0.843	0.827	0.955	0.879	0.752	0.905	0.881	0.782
**Helstrom Quantum Classifier**	0.752	0.800	0.778	0.750	0.733	0.958	0.807	0.881	0.857	0.885	0.907	0.836	0.877	0.806	0.816	0.765	0.881	0.780	0.877	0.950	0.871	0.874	0.861	0.875	0.970	0.824	0.688	0.851	0.863	0.781
Linear Discriminant Analysis	0.563	0.850	0.833	0.650	0.625	0.792	0.724	0.750	0.857	0.846	0.764	0.722	0.744	0.764	0.660	0.721	0.787	0.704	0.834	0.700	0.764	0.725	0.692	0.820	0.735	0.824	0.647	0.695	0.600	0.806
Logistic Regression	0.730	0.950	0.833	0.650	0.625	0.833	0.918	0.781	0.857	0.941	0.871	0.864	0.777	0.800	0.782	0.765	0.881	0.724	0.899	0.800	0.885	0.890	0.731	0.835	0.735	0.824	0.688	0.820	0.650	0.808
Multi Layer Perceptron	0.835	0.900	0.927	0.800	0.625	0.833	0.890	0.831	0.857	0.885	0.907	0.864	0.777	0.800	0.787	0.765	0.881	0.835	0.877	0.900	0.907	0.826	0.843	0.811	0.955	0.879	0.688	0.851	0.850	0.808
Nearest Neighbors	0.813	0.900	0.833	0.800	0.778	0.938	0.890	0.831	0.857	0.923	0.849	0.836	0.832	0.907	0.871	0.798	0.826	0.835	0.863	0.850	0.835	0.826	0.881	0.882	0.860	0.895	0.730	0.835	0.850	0.782
Quadratic Discriminant Analysis	0.625	1.000	0.889	0.700	0.625	0.833	0.779	0.800	0.857	0.962	0.907	0.808	0.777	0.764	0.719	0.721	0.786	0.760	0.921	0.750	0.885	0.780	0.692	0.851	0.610	0.824	0.688	0.757	0.600	0.781
Random Forest	0.855	0.950	0.964	0.750	0.795	0.920	0.918	0.831	0.857	0.941	0.907	0.946	0.877	0.849	0.841	0.776	0.826	0.835	0.877	0.781	0.885	0.914	0.881	0.890	0.860	0.879	0.752	0.897	0.800	0.782
SVM - linear	0.772	0.900	0.833	0.600	0.608	0.958	0.779	0.781	0.857	0.885	0.871	0.697	0.777	0.835	0.812	0.755	0.857	0.760	0.877	0.750	0.885	0.945	0.710	0.811	0.845	0.824	0.688	0.820	0.650	0.808
SVM - poly	0.730	0.850	0.778	0.650	0.625	0.833	0.724	0.731	0.857	0.885	0.835	0.668	0.777	0.800	0.799	0.721	0.857	0.760	0.899	0.800	0.871	0.866	0.899	0.851	0.625	0.824	0.647	0.780	0.650	0.806
SVM - rbf	0.813	0.500	0.500	0.500	0.500	0.667	0.890	0.500	0.832	0.731	0.871	0.808	0.500	0.835	0.812	0.732	0.857	0.649	0.877	0.500	0.500	0.976	0.615	0.811	0.500	0.500	0.688	0.500	0.700	0.808

Results exclude Nearest Centroid, Passive Aggressive Classifier and Perceptron.

Table 4.22 | AUROC score for 30 correlation image feature datasets for cell line U87-MG

Classifiers	P1	P2	P3	P4	P5	P6	P7	P8	P9	P10	P11	P12	P13	P14	P15	P16	P17	P18	P19	P20	P21	P22	P23	P24	P25	P26	P27	P28	P29	P30
AdaBoost	0.750	0.844	0.651	0.608	0.760	0.752	0.753	0.769	0.809	0.539	0.500	0.744	0.704	0.753	0.607	0.716	0.557	0.814	0.754	0.734	0.719	0.732	0.837	0.631	0.500	0.669	0.500	0.885	0.817	0.724
Bernoulli Naive Bayes	0.781	0.851	0.655	0.545	0.500	0.500	0.697	0.551	0.688	0.726	0.605	0.644	0.585	0.702	0.525	0.644	0.532	0.570	0.614	0.631	0.601	0.632	0.647	0.661	0.500	0.669	0.520	0.621	0.685	0.803
Dummy Classifier	0.409	0.500	0.500	0.500	0.372	0.500	0.500	0.470	0.500	0.379	0.588	0.541	0.500	0.500	0.528	0.534	0.500	0.526	0.500	0.528	0.403	0.594	0.500	0.500	0.500	0.500	0.427	0.180	0.505	0.406
Extra Trees	0.500	0.500	0.500	0.500	0.500	0.500	0.500	0.500	0.533	0.500	0.500	0.500	0.500	0.500	0.500	0.643	0.500	0.500	0.500	0.500	0.500	0.593	0.500	0.500	0.500	0.500	0.500	0.500	0.500	0.681
Gaussian Naive Bayes	0.700	0.753	0.706	0.591	0.643	0.730	0.699	0.618	0.665	0.695	0.587	0.611	0.606	0.753	0.607	0.644	0.532	0.707	0.699	0.820	0.695	0.707	0.752	0.624	0.500	0.669	0.635	0.729	0.702	0.832
Gradient Boosting	0.800	0.864	0.817	0.670	0.767	0.793	0.725	0.952	0.842	0.562	0.815	0.867	0.606	0.646	0.777	0.810	0.732	0.849	0.702	0.897	0.781	0.757	0.815	0.717	0.511	0.753	0.748	0.748	0.750	0.669
**Helstrom Quantum Classifier**	0.913	0.897	0.839	0.782	0.708	0.712	0.751	0.739	0.786	0.734	0.758	0.844	0.676	0.751	0.717	0.882	0.653	0.699	0.813	0.858	0.765	0.653	0.918	0.701	0.754	0.644	0.828	0.748	0.753	0.757
Linear Discriminant Analysis	0.531	0.773	0.688	0.573	0.572	0.585	0.728	0.618	0.598	0.695	0.545	0.677	0.630	0.702	0.587	0.723	0.507	0.627	0.697	0.878	0.609	0.707	0.690	0.528	0.500	0.653	0.585	0.593	0.602	0.774
Logistic Regression	0.531	0.773	0.706	0.591	0.526	0.585	0.728	0.618	0.598	0.695	0.545	0.644	0.630	0.702	0.587	0.699	0.507	0.627	0.697	0.878	0.609	0.707	0.690	0.528	0.500	0.653	0.585	0.593	0.602	0.744
Multi Layer Perceptron	0.800	0.916	0.690	0.608	0.696	0.797	0.784	0.720	0.898	0.750	0.755	0.833	0.606	0.702	0.778	0.921	0.778	0.790	0.754	0.916	0.726	0.728	0.755	0.780	0.716	0.669	0.856	0.736	0.817	0.774
Nearest Neighbors	0.831	0.780	0.762	0.608	0.696	0.837	0.781	0.820	0.820	0.702	0.777	0.777	0.603	0.728	0.650	0.874	0.619	0.705	0.700	0.897	0.695	0.632	0.898	0.765	0.653	0.638	0.806	0.626	0.702	0.782
Quadratic Discriminant Analysis	0.681	0.753	0.671	0.591	0.643	0.710	0.697	0.602	0.586	0.781	0.630	0.677	0.627	0.702	0.628	0.644	0.532	0.649	0.727	0.878	0.640	0.762	0.753	0.662	0.562	0.669	0.685	0.528	0.618	0.719
Random Forest	0.800	0.734	0.651	0.670	0.806	0.855	0.753	0.968	0.876	0.695	0.815	0.833	0.627	0.727	0.713	0.833	0.753	0.734	0.670	0.897	0.726	0.728	0.898	0.749	0.625	0.669	0.756	0.741	0.801	0.782
SVM - linear	0.500	0.799	0.482	0.608	0.500	0.500	0.728	0.500	0.598	0.500	0.585	0.544	0.500	0.728	0.587	0.708	0.532	0.500	0.727	0.813	0.601	0.707	0.585	0.521	0.500	0.500	0.500	0.547	0.551	0.774
SVM - poly	0.831	0.753	0.633	0.625	0.787	0.837	0.703	0.621	0.853	0.640	0.797	0.677	0.582	0.702	0.607	0.890	0.607	0.713	0.700	0.916	0.734	0.703	0.753	0.679	0.761	0.653	0.699	0.626	0.868	0.656
SVM - rbf	0.500	0.799	0.669	0.500	0.500	0.500	0.782	0.583	0.665	0.656	0.565	0.544	0.500	0.500	0.500	0.500	0.525	0.500	0.500	0.500	0.577	0.707	0.690	0.600	0.500	0.500	0.500	0.500	0.602	0.774

Results exclude Nearest Centroid, Passive Aggressive Classifier and Perceptron.

Table 4.23 | AUROC score for 30 energy image feature datasets for cell line U87-MG

Classifiers	P1	P2	P3	P4	P5	P6	P7	P8	P9	P10	P11	P12	P13	P14	P15	P16	P17	P18	P19	P20	P21	P22	P23	P24	P25	P26	P27	P28	P29	P30
AdaBoost	0.760	0.940	0.900	0.935	0.752	0.920	0.977	0.667	0.902	0.804	1.000	0.933	0.764	0.920	0.938	0.871	0.834	0.875	0.888	1.000	0.730	0.898	0.916	0.837	0.833	0.985	0.818	0.878	0.800	0.925
Bernoulli Naive Bayes	0.762	0.898	0.953	0.820	0.500	0.828	0.886	0.651	0.846	0.842	0.863	0.820	0.748	0.886	0.591	0.863	0.703	0.825	0.708	0.522	0.692	0.798	0.671	0.900	0.500	0.985	0.710	0.833	0.684	0.841
Dummy Classifier	0.500	0.500	0.500	0.500	0.500	0.500	0.500	0.500	0.446	0.488	0.500	0.500	0.500	0.500	0.500	0.500	0.500	0.500	0.500	0.500	0.500	0.590	0.689	0.490	0.500	0.500	0.334	0.466	0.544	0.612
Extra Trees	0.500	0.500	0.500	0.500	0.500	0.500	0.500	0.500	0.654	0.562	0.500	0.500	0.500	0.500	0.500	0.693	0.500	0.500	0.577	0.500	0.500	0.500	0.500	0.797	0.500	0.500	0.500	0.479	0.500	0.500
Gaussian Naive Bayes	0.944	0.958	0.984	0.887	0.835	0.920	0.955	0.853	0.941	0.897	0.972	0.888	0.849	0.869	0.906	0.871	0.682	0.850	0.876	1.000	0.877	0.838	0.839	0.920	0.785	0.985	0.916	0.837	0.900	0.866
Gradient Boosting	0.871	0.940	1.000	0.884	0.752	0.938	0.977	0.669	0.923	0.827	0.918	0.933	0.835	0.920	0.969	0.828	0.812	0.832	0.921	0.980	0.772	0.855	0.851	0.898	0.833	0.985	0.851	0.881	0.800	0.921
**Helstrom Quantum Classifier**	0.927	0.940	0.984	0.903	0.815	0.862	0.898	0.911	1.000	0.905	0.889	0.921	0.935	0.886	0.891	0.907	0.725	0.925	0.876	0.980	0.920	0.838	0.858	0.880	0.903	0.985	0.806	0.878	0.969	0.946
Linear Discriminant Analysis	0.889	0.898	0.984	0.919	0.688	0.920	0.932	0.778	0.979	0.859	0.917	0.832	0.828	0.920	0.922	0.849	0.595	0.850	0.876	0.688	0.815	0.857	0.820	0.880	0.500	0.985	0.799	0.857	0.800	0.816
Logistic Regression	0.927	0.938	0.984	0.935	0.752	0.920	0.977	0.833	0.979	0.804	0.972	0.933	0.800	0.841	0.906	0.835	0.747	0.925	0.898	0.958	0.813	0.878	0.858	0.837	0.817	0.985	0.799	0.819	0.800	0.841
Multi Layer Perceptron	0.944	0.938	0.984	0.968	0.752	0.920	0.911	0.815	0.979	0.804	0.972	0.933	0.800	0.841	0.906	0.814	0.747	0.946	0.944	0.980	0.813	0.878	0.897	0.857	0.500	0.985	0.844	0.819	0.900	0.921
Nearest Neighbors	0.944	0.918	0.900	0.785	0.793	0.920	0.911	0.927	1.000	0.827	0.944	0.955	0.814	0.841	0.938	0.828	0.747	0.946	0.888	0.960	0.877	0.795	0.832	0.900	0.718	1.000	0.844	0.801	0.984	0.971
Quadratic Discriminant Analysis	0.889	0.958	0.984	0.887	0.793	0.938	0.932	0.815	0.941	0.929	0.944	0.888	0.849	0.869	0.891	0.871	0.682	0.850	0.853	1.000	0.918	0.797	0.858	0.920	0.618	1.000	0.871	0.857	0.900	0.866
Random Forest	0.871	0.960	1.000	0.968	0.752	0.938	0.977	0.833	0.979	0.827	0.947	0.933	0.800	0.920	0.969	0.849	0.790	0.950	0.944	1.000	0.833	0.835	0.916	0.898	0.901	0.985	0.818	0.861	0.800	0.950
SVM - linear	0.927	0.960	0.984	0.935	0.835	0.920	0.955	0.778	0.979	0.835	0.972	0.911	0.863	0.903	0.906	0.835	0.703	0.900	0.898	0.958	0.813	0.858	0.858	0.900	0.833	0.985	0.935	0.857	0.900	0.841
SVM - poly	0.944	0.938	1.000	0.968	0.752	0.920	0.865	0.778	0.885	0.835	0.944	0.888	0.828	0.903	0.922	0.871	0.682	0.875	0.944	0.833	0.835	0.837	0.878	0.900	0.500	0.971	0.825	0.881	0.900	0.841
SVM - rbf	0.927	0.960	0.984	0.903	0.835	0.920	0.977	0.853	0.979	0.835	0.972	0.911	0.828	0.903	0.906	0.835	0.500	0.925	0.898	0.958	0.835	0.858	0.820	0.878	0.833	0.985	0.500	0.500	0.900	0.841

Results exclude Nearest Centroid, Passive Aggressive Classifier and Perceptron.

Table 4.24 | AUROC score for 30 homogeneity image feature datasets for cell line U87-MG

Classifiers	P1	P2	P3	P4	P5	P6	P7	P8	P9	P10	P11	P12	P13	P14	P15	P16	P17	P18	P19	P20	P21	P22	P23	P24	P25	P26	P27	P28	P29	P30
AdaBoost	0.931	0.985	0.938	0.733	0.653	0.964	0.981	0.920	0.978	0.842	0.855	0.921	0.819	0.918	0.789	0.979	0.946	0.810	0.888	0.935	0.929	0.865	0.897	0.876	0.935	0.938	1.000	0.864	0.944	0.895
Bernoulli Naive Bayes	0.931	0.970	0.952	0.744	0.789	1.000	0.962	0.869	0.935	0.826	0.858	0.871	0.854	0.833	0.897	0.896	0.946	0.810	0.876	0.942	0.957	0.893	0.904	0.757	0.715	0.886	0.918	0.840	0.631	0.816
Dummy Classifier	0.483	0.614	0.609	0.500	0.500	0.500	0.573	0.647	0.453	0.500	0.363	0.500	0.500	0.550	0.545	0.463	0.500	0.500	0.483	0.528	0.500	0.500	0.664	0.459	0.500	0.500	0.500	0.532	0.500	0.522
Extra Trees	0.500	0.500	0.500	0.500	0.500	0.500	0.799	0.500	0.893	0.500	0.708	0.500	0.500	0.667	0.656	0.577	0.918	0.893	0.877	0.500	0.500	0.864	0.500	0.870	0.500	0.500	0.500	0.500	0.500	0.658
Gaussian Naive Bayes	0.931	0.985	0.969	0.869	0.823	1.000	0.935	0.920	0.978	0.828	0.938	0.921	0.837	0.918	0.835	0.958	0.946	0.810	0.944	0.935	0.929	0.865	0.897	0.757	0.844	0.920	0.897	0.902	1.000	0.868
Gradient Boosting	0.931	0.985	0.844	0.858	0.716	1.000	0.935	0.778	0.943	0.863	0.855	0.921	0.692	0.855	0.679	0.938	0.972	0.810	0.898	0.935	0.871	0.841	0.942	0.857	0.760	0.920	1.000	0.801	0.833	0.895
**Helstrom Quantum Classifier**	0.931	0.985	1.000	0.920	0.869	1.000	0.935	0.903	0.943	0.891	0.898	0.921	0.837	0.898	0.804	0.979	0.946	0.893	0.865	0.962	0.929	0.839	0.962	0.876	0.871	0.903	0.960	0.902	0.946	0.947
Linear Discriminant Analysis	0.931	0.985	0.969	0.869	0.698	1.000	0.890	0.903	0.957	0.863	0.918	0.921	0.857	0.918	0.827	0.979	0.946	0.838	0.911	0.897	0.929	0.865	0.897	0.757	0.844	0.920	0.917	0.902	1.000	0.895
Logistic Regression	0.931	0.985	0.969	0.903	0.653	1.000	0.890	0.920	0.978	0.863	0.938	0.921	0.881	0.918	0.827	0.958	0.946	0.838	0.911	0.935	0.893	0.865	0.897	0.807	0.753	0.938	0.917	0.864	0.944	0.868
Multi Layer Perceptron	0.931	0.985	0.969	0.903	0.636	0.929	0.916	0.920	0.978	0.863	0.938	0.921	0.881	0.898	0.796	0.958	0.972	0.838	0.888	0.935	0.929	0.865	0.916	0.857	0.890	0.938	0.958	0.902	0.944	0.763
Nearest Neighbors	0.931	0.985	0.969	0.823	0.716	0.964	0.890	0.920	0.964	0.935	0.877	0.941	0.878	0.898	0.890	0.902	0.972	0.920	0.888	0.916	0.893	0.865	0.916	0.876	0.844	0.920	0.813	0.902	0.944	0.921
Quadratic Discriminant Analysis	0.931	0.985	0.969	0.869	0.823	1.000	0.890	0.903	0.978	0.863	0.855	0.921	0.878	0.918	0.859	0.958	0.946	0.865	0.911	0.935	0.929	0.865	0.916	0.857	0.799	0.920	0.938	0.902	1.000	0.868
Random Forest	0.931	0.985	0.938	0.858	0.653	1.000	0.981	0.903	0.943	0.863	0.855	0.921	0.878	0.938	0.859	1.000	0.972	0.811	0.911	0.935	0.929	0.865	0.897	0.907	0.799	0.938	0.938	0.864	0.889	0.895
SVM - linear	0.931	0.985	0.938	0.903	0.608	0.929	0.890	0.920	0.957	0.842	0.938	0.921	0.878	0.918	0.796	0.979	0.946	0.838	0.888	0.916	0.929	0.893	0.897	0.757	0.753	0.938	0.875	0.864	0.944	0.868
SVM - poly	0.800	1.000	0.938	0.858	0.545	0.964	0.935	0.920	0.943	0.863	0.938	0.891	0.881	0.708	0.796	0.941	0.946	0.865	0.888	0.916	0.929	0.867	0.871	0.857	0.753	0.875	0.813	0.861	0.722	0.842
SVM - rbf	0.500	1.000	0.500	0.903	0.636	0.500	0.935	0.920	0.500	0.500	0.500	0.921	0.861	0.918	0.796	0.979	0.946	0.864	0.888	0.935	0.500	0.865	0.844	0.757	0.753	0.938	0.500	0.864	0.722	0.868

Results exclude Nearest Centroid, Passive Aggressive Classifier and Perceptron.

Table 4.25 | Balanced accuracy score for 30 RGB image feature datasets for cell line MCF7

Classifiers	P1	P2	P3	P4	P5	P6	P7	P8	P9	P10	P11	P12	P13	P14	P15	P16	P17	P18	P19	P20	P21	P22	P23	P24	P25	P26	P27	P28	P29	P30
AdaBoost	0.885	1.000	0.944	0.899	0.920	1.000	0.944	0.938	0.982	0.972	0.957	1.000	0.941	0.940	0.941	0.800	1.000	0.980	1.000	1.000	0.929	1.000	0.884	1.000	0.970	0.971	1.000	0.982	1.000	1.000
Bernoulli Naive Bayes	0.863	1.000	0.944	0.881	0.958	1.000	0.944	0.938	0.964	0.972	0.907	1.000	0.979	1.000	0.941	0.969	0.942	0.980	0.985	1.000	0.929	1.000	0.869	1.000	0.955	0.946	0.977	0.800	0.913	0.984
Dummy Classifier	0.362	0.456	0.472	0.365	0.585	0.500	0.500	0.593	0.500	0.500	0.500	0.500	0.662	0.552	0.500	0.500	0.500	0.508	0.424	0.500	0.531	0.500	0.422	0.500	0.500	0.500	0.500	0.633	0.583	0.500
Extra Trees	0.885	1.000	0.778	0.902	0.923	0.792	0.944	0.938	0.944	0.974	0.929	0.923	0.979	0.958	0.941	0.600	0.955	0.938	1.000	1.000	0.929	0.800	0.500	0.950	0.500	0.912	0.867	0.500	0.781	0.700
Gaussian Naive Bayes	0.863	1.000	0.944	0.902	0.920	1.000	0.944	0.938	0.944	0.972	0.907	0.979	0.979	0.980	0.941	0.900	0.942	0.960	1.000	1.000	0.895	0.950	0.884	1.000	0.985	0.971	0.955	0.835	0.913	0.900
Gradient Boosting	0.885	1.000	0.833	0.958	0.902	0.917	0.944	0.938	0.927	0.944	0.943	0.923	0.979	0.940	0.941	0.700	1.000	0.980	1.000	1.000	1.000	1.000	0.769	1.000	0.720	0.941	1.000	0.927	1.000	1.000
**Helstrom Quantum Classifier**	0.935	1.000	0.944	0.902	0.902	1.000	0.944	0.938	0.982	0.946	0.964	1.000	0.979	1.000	0.941	0.984	0.962	0.938	1.000	1.000	0.912	0.900	0.884	1.000	0.750	0.971	0.888	0.964	0.981	0.969
Linear Discriminant Analysis	0.921	1.000	0.944	0.920	0.920	0.958	0.889	0.938	1.000	0.944	0.964	1.000	0.979	1.000	0.941	1.000	0.955	0.938	1.000	1.000	0.929	0.900	0.784	0.950	0.970	0.946	0.933	0.889	1.000	0.900
Logistic Regression	0.885	1.000	0.889	0.962	0.923	0.875	0.889	0.938	1.000	0.944	1.000	1.000	0.979	1.000	0.941	0.800	1.000	0.938	1.000	1.000	0.929	1.000	0.884	1.000	0.845	0.946	1.000	0.909	1.000	0.900
Multi Layer Perceptron	0.913	1.000	0.944	0.962	0.881	0.875	0.889	0.938	0.944	0.944	1.000	1.000	0.979	1.000	0.941	0.900	0.955	0.938	1.000	1.000	0.912	1.000	0.784	1.000	0.845	0.946	0.944	0.982	0.950	1.000
Nearest Centroid	0.899	1.000	0.889	0.902	0.881	0.958	0.944	0.855	0.944	0.946	0.907	0.902	0.979	1.000	0.941	1.000	0.909	0.938	1.000	1.000	0.912	0.850	0.784	1.000	0.955	0.916	0.911	0.817	0.863	0.900
Nearest Neighbors	0.935	1.000	1.000	0.979	0.881	0.958	0.982	0.938	0.944	0.974	0.964	1.000	0.902	1.000	0.941	0.784	0.981	0.938	1.000	1.000	0.912	0.950	0.784	1.000	0.985	0.971	0.967	0.982	1.000	0.884
Passive Aggressive Classifier	0.842	0.981	0.889	0.962	0.902	0.875	0.889	0.980	1.000	0.944	0.978	1.000	0.979	0.960	0.979	0.900	0.955	0.918	1.000	1.000	0.929	1.000	0.884	1.000	0.970	0.921	0.977	0.853	0.981	0.900
Perceptron	0.842	1.000	0.944	0.854	0.962	0.813	0.889	0.980	0.944	0.944	0.978	0.962	0.979	0.900	0.941	0.900	0.955	0.918	1.000	1.000	0.929	1.000	0.884	1.000	0.970	0.921	1.000	0.964	0.981	0.800
Quadratic Discriminant Analysis	0.921	1.000	1.000	0.941	0.902	0.958	0.944	0.938	0.982	0.944	0.978	1.000	0.979	0.960	0.941	1.000	0.962	0.960	0.985	1.000	1.000	1.000	0.884	1.000	0.750	0.946	0.955	0.944	0.981	1.000
Random Forest	0.885	1.000	0.889	0.896	0.941	0.917	0.944	0.938	0.982	0.972	0.943	1.000	0.979	0.940	0.941	0.700	1.000	0.980	1.000	1.000	0.929	1.000	0.869	1.000	0.845	0.971	1.000	0.982	1.000	1.000
SVM - linear	0.899	1.000	0.944	0.941	0.941	0.917	1.000	0.980	0.982	0.944	0.978	1.000	0.979	0.980	0.979	0.900	1.000	0.938	1.000	1.000	0.912	1.000	0.784	1.000	0.845	0.975	0.977	0.964	0.981	0.900
SVM - poly	0.863	1.000	0.927	0.902	0.920	0.917	1.000	0.938	1.000	0.918	0.978	1.000	0.979	1.000	0.941	0.800	1.000	0.938	1.000	1.000	0.912	0.900	0.900	1.000	0.860	0.975	0.967	0.927	0.850	0.884
SVM - rbf	0.885	1.000	0.889	0.899	0.941	0.917	0.944	0.938	1.000	0.944	0.978	1.000	0.979	0.980	0.979	0.900	1.000	0.938	1.000	1.000	0.929	1.000	0.784	1.000	0.845	0.975	0.977	0.982	1.000	0.900

Table 4.26 | Balanced accuracy score for 30 L*u*v* image feature datasets for cell line MCF7

Classifiers	P1	P2	P3	P4	P5	P6	P7	P8	P9	P10	P11	P12	P13	P14	P15	P16	P17	P18	P19	P20	P21	P22	P23	P24	P25	P26	P27	P28	P29	P30
AdaBoost	0.885	1.000	0.944	0.896	0.979	0.917	0.944	0.938	0.982	0.972	0.943	1.000	0.941	0.940	0.941	0.700	0.962	0.960	1.000	1.000	0.929	1.000	0.884	1.000	0.720	0.946	0.977	0.944	0.981	0.969
Bernoulli Naive Bayes	0.935	1.000	1.000	0.861	0.938	0.980	0.982	0.980	0.964	0.944	0.921	1.000	0.979	0.940	0.979	0.969	0.942	0.940	0.970	1.000	0.967	1.000	0.969	0.981	0.924	0.975	0.955	0.982	0.981	0.953
Dummy Classifier	0.500	0.500	0.500	0.522	0.484	0.443	0.435	0.500	0.562	0.500	0.475	0.500	0.500	0.467	0.502	0.606	0.500	0.500	0.500	0.500	0.500	0.539	0.500	0.639	0.500	0.500	0.518	0.500	0.500	0.575
Extra Trees	0.885	1.000	0.944	0.899	0.979	0.958	0.944	0.938	0.944	0.946	0.943	1.000	0.979	1.000	0.941	0.700	0.981	0.918	1.000	1.000	0.929	0.850	0.500	1.000	0.500	0.971	0.977	0.927	0.981	0.900
Gaussian Naive Bayes	0.899	1.000	1.000	0.861	0.979	1.000	1.000	0.938	0.982	0.944	0.943	1.000	0.979	0.940	0.979	1.000	0.942	0.940	0.985	1.000	0.983	1.000	0.984	1.000	0.985	0.946	0.955	0.927	0.981	0.984
Gradient Boosting	0.863	1.000	0.944	0.920	0.923	0.958	0.889	0.855	0.889	0.972	0.929	0.962	0.979	0.898	0.979	0.900	1.000	0.898	1.000	1.000	0.929	1.000	0.869	1.000	0.720	0.946	0.977	0.982	1.000	1.000
**Helstrom Quantum Classifier**	0.913	1.000	1.000	0.938	0.941	0.958	0.982	0.938	0.982	0.918	1.000	1.000	0.958	0.960	0.941	0.984	0.981	0.940	0.985	1.000	0.967	1.000	0.984	1.000	0.875	0.946	0.911	0.982	1.000	0.969
Linear Discriminant Analysis	0.921	1.000	0.944	0.920	0.979	1.000	0.889	0.938	1.000	0.944	1.000	1.000	0.979	0.960	0.941	1.000	1.000	0.960	1.000	1.000	0.929	1.000	0.784	1.000	0.970	0.946	1.000	0.944	0.981	0.900
Logistic Regression	0.885	1.000	0.833	0.941	0.941	0.917	0.889	0.938	1.000	0.918	1.000	1.000	0.979	0.960	0.979	1.000	1.000	0.898	1.000	1.000	0.929	1.000	0.784	1.000	0.860	0.946	1.000	0.853	1.000	1.000
Multi Layer Perceptron	0.899	1.000	1.000	0.941	0.941	0.958	1.000	0.980	0.982	0.918	1.000	1.000	0.979	0.960	0.979	0.900	1.000	0.940	0.985	1.000	0.929	1.000	0.984	1.000	0.860	0.975	1.000	0.853	0.981	1.000
Nearest Centroid	0.935	1.000	0.944	0.899	0.938	1.000	1.000	0.938	0.982	0.918	0.943	1.000	0.979	0.940	0.979	0.984	0.962	0.940	0.985	1.000	0.895	1.000	0.984	1.000	0.955	0.946	0.977	0.927	0.981	1.000
Nearest Neighbors	0.957	1.000	1.000	0.878	0.843	0.917	1.000	0.980	0.927	0.918	1.000	1.000	0.979	0.938	0.941	0.884	0.981	0.918	0.985	1.000	0.929	1.000	0.800	1.000	0.750	0.946	1.000	0.944	1.000	1.000
Passive Aggressive Classifier	0.863	1.000	0.833	0.920	0.885	0.875	0.889	0.855	0.944	0.944	1.000	1.000	0.979	0.940	0.979	0.900	0.962	0.918	1.000	1.000	0.929	1.000	0.884	1.000	0.985	0.946	1.000	0.927	0.950	0.984
Perceptron	0.835	1.000	0.778	0.962	0.923	0.875	0.889	0.980	0.944	0.918	1.000	0.885	0.979	0.960	0.941	0.800	1.000	0.878	1.000	1.000	0.929	1.000	0.584	1.000	0.750	0.975	0.967	0.964	0.981	1.000
Quadratic Discriminant Analysis	0.885	1.000	1.000	0.941	0.902	0.958	0.944	0.938	0.964	0.944	1.000	1.000	0.979	0.960	0.941	0.900	0.981	0.918	0.985	1.000	0.929	1.000	0.884	1.000	1.000	0.946	0.955	1.000	0.981	0.984
Random Forest	0.885	1.000	0.944	0.917	0.979	0.958	0.889	0.938	0.982	0.944	0.907	1.000	0.979	0.940	0.941	0.800	0.962	0.940	1.000	1.000	0.929	1.000	0.984	1.000	0.985	0.946	0.977	1.000	0.981	0.984
SVM - linear	0.921	1.000	1.000	0.920	0.941	0.958	0.889	0.980	1.000	0.918	1.000	0.962	0.979	0.940	0.979	1.000	1.000	0.898	0.985	1.000	0.929	1.000	0.784	1.000	0.845	0.946	1.000	0.927	1.000	0.984
SVM - poly	0.885	1.000	1.000	0.941	0.941	0.958	0.889	0.980	0.982	0.918	1.000	1.000	0.979	0.960	0.941	0.800	1.000	0.918	0.985	1.000	0.929	1.000	0.784	1.000	0.860	0.946	1.000	0.871	1.000	1.000
SVM - rbf	0.921	1.000	1.000	0.920	0.941	1.000	0.944	0.938	0.982	0.944	1.000	0.962	0.979	0.960	0.979	1.000	1.000	0.940	1.000	1.000	0.929	1.000	0.784	1.000	0.860	0.975	1.000	0.927	1.000	0.984

Table 4.27 | Balanced accuracy score for 30 contrast image feature datasets for cell line MCF7

Classifiers	P1	P2	P3	P4	P5	P6	P7	P8	P9	P10	P11	P12	P13	P14	P15	P16	P17	P18	P19	P20	P21	P22	P23	P24	P25	P26	P27	P28	P29	P30
AdaBoost	0.870	0.981	0.964	0.753	0.700	0.897	0.911	0.920	0.800	0.861	0.711	0.917	0.854	0.858	0.721	0.769	0.839	0.677	0.830	0.920	0.883	0.944	0.669	0.926	0.500	0.715	0.820	0.800	0.894	0.891
Bernoulli Naive Bayes	0.762	0.890	0.964	0.686	0.739	0.835	0.853	0.855	0.722	0.706	0.699	0.745	0.665	0.650	0.591	0.569	0.741	0.423	0.720	0.614	0.560	0.844	0.500	0.813	0.500	0.715	0.786	0.744	0.807	0.822
Dummy Classifier	0.500	0.500	0.562	0.345	0.500	0.500	0.526	0.500	0.506	0.491	0.584	0.500	0.421	0.487	0.500	0.500	0.500	0.500	0.500	0.500	0.481	0.500	0.538	0.370	0.485	0.434	0.500	0.579	0.500	0.500
Extra Trees	0.500	0.500	0.500	0.704	0.500	0.500	0.500	0.500	0.500	0.500	0.500	0.500	0.500	0.500	0.500	0.500	0.500	0.500	0.500	0.500	0.500	0.500	0.500	0.500	0.500	0.500	0.500	0.500	0.500	0.500
Gaussian Naive Bayes	0.778	0.799	0.760	0.668	0.627	0.625	0.760	0.750	0.611	0.632	0.542	0.612	0.591	0.565	0.686	0.500	0.598	0.503	0.735	0.532	0.417	0.750	0.584	0.631	0.610	0.538	0.776	0.722	0.631	0.684
Gradient Boosting	0.848	0.981	0.964	0.697	0.718	0.855	0.855	0.877	0.744	0.813	0.776	0.840	0.798	0.733	0.899	0.853	0.923	0.528	0.955	0.863	0.862	0.844	0.669	0.857	0.595	0.921	0.764	0.724	0.844	0.822
**Helstrom Quantum Classifier**	0.848	0.981	0.891	0.694	0.603	0.835	0.837	0.918	0.780	0.759	0.891	0.822	0.780	0.757	0.801	0.759	0.839	0.427	0.845	0.810	0.767	0.739	0.622	0.763	0.970	0.862	0.853	0.762	0.907	0.828
Linear Discriminant Analysis	0.756	0.708	0.631	0.707	0.588	0.627	0.760	0.792	0.611	0.550	0.562	0.612	0.591	0.565	0.532	0.500	0.500	0.442	0.735	0.531	0.510	0.750	0.584	0.650	0.610	0.538	0.776	0.760	0.731	0.569
Logistic Regression	0.756	0.818	0.687	0.686	0.683	0.668	0.760	0.833	0.667	0.602	0.699	0.612	0.609	0.565	0.532	0.500	0.500	0.462	0.735	0.531	0.510	0.731	0.484	0.631	0.485	0.568	0.776	0.760	0.713	0.569
Multi Layer Perceptron	0.762	0.890	0.927	0.638	0.620	0.855	0.946	0.833	0.889	0.784	0.719	0.728	0.780	0.733	0.822	0.553	0.897	0.548	0.720	0.836	0.740	0.744	0.584	0.713	0.610	0.744	0.720	0.817	0.857	0.806
Nearest Centroid	0.820	0.839	0.891	0.837	0.736	0.960	0.817	0.735	0.837	0.781	0.648	0.662	0.700	0.713	0.756	0.597	0.858	0.510	0.970	0.487	0.674	0.683	0.822	0.844	0.955	0.803	0.753	0.708	0.839	0.875
Nearest Neighbors	0.842	0.916	0.871	0.795	0.659	0.793	0.837	0.918	0.798	0.703	0.812	0.724	0.728	0.673	0.647	0.769	0.962	0.550	0.720	0.863	0.740	0.831	0.553	0.581	0.625	0.832	0.786	0.762	0.944	0.822
Passive Aggressive Classifier	0.734	0.818	0.909	0.647	0.774	0.675	0.817	0.837	0.800	0.579	0.683	0.500	0.712	0.563	0.593	0.600	0.624	0.480	0.860	0.436	0.674	0.776	0.584	0.713	0.799	0.656	0.842	0.780	0.683	0.544
Perceptron	0.734	0.773	0.798	0.513	0.662	0.710	0.817	0.918	0.611	0.538	0.604	0.476	0.309	0.512	0.434	0.600	0.491	0.403	0.485	0.518	0.631	0.813	0.822	0.763	0.985	0.685	0.853	0.744	0.489	0.875
Quadratic Discriminant Analysis	0.814	0.818	0.815	0.707	0.627	0.667	0.704	0.708	0.611	0.548	0.534	0.591	0.591	0.607	0.571	0.500	0.624	0.462	0.735	0.560	0.488	0.731	0.584	0.650	0.610	0.538	0.776	0.704	0.681	0.584
Random Forest	0.820	1.000	0.964	0.753	0.739	0.897	0.946	0.938	0.780	0.756	0.784	0.899	0.875	0.817	0.801	0.653	0.858	0.672	0.970	0.918	0.812	0.863	0.600	0.826	0.500	0.799	0.786	0.706	0.931	0.822
SVM - linear	0.770	0.890	0.853	0.686	0.721	0.710	0.853	0.897	0.667	0.550	0.699	0.630	0.665	0.565	0.434	0.500	0.500	0.462	0.860	0.529	0.433	0.650	0.500	0.631	0.500	0.597	0.786	0.780	0.713	0.600
SVM - poly	0.727	0.864	0.964	0.732	0.638	0.980	0.911	0.878	0.649	0.759	0.705	0.787	0.774	0.753	0.784	0.669	0.878	0.612	0.720	0.838	0.829	0.776	0.553	0.694	0.500	0.744	0.753	0.762	0.826	0.806
SVM - rbf	0.699	0.890	0.964	0.784	0.718	0.793	0.853	0.877	0.667	0.654	0.720	0.686	0.665	0.607	0.490	0.500	0.533	0.462	0.860	0.557	0.505	0.650	0.500	0.631	0.500	0.626	0.786	0.762	0.713	0.769

Table 4.28 | Balanced accuracy score for 30 correlation image feature datasets for cell line MCF7

Classifiers	P1	P2	P3	P4	P5	P6	P7	P8	P9	P10	P11	P12	P13	P14	P15	P16	P17	P18	P19	P20	P21	P22	P23	P24	P25	P26	P27	P28	P29	P30
AdaBoost	0.610	0.708	0.800	0.593	0.718	0.625	0.690	0.857	0.728	0.750	0.761	0.780	0.795	0.733	0.676	0.469	0.734	0.670	0.784	0.699	0.762	0.650	0.469	0.844	0.500	0.765	0.786	0.722	0.713	0.591
Bernoulli Naive Bayes	0.661	0.572	0.633	0.596	0.676	0.667	0.673	0.878	0.671	0.779	0.756	0.612	0.798	0.757	0.683	0.500	0.657	0.592	0.549	0.754	0.740	0.757	0.500	0.752	0.500	0.631	0.830	0.633	0.402	0.622
Dummy Classifier	0.500	0.500	0.500	0.484	0.383	0.500	0.415	0.500	0.500	0.500	0.500	0.490	0.500	0.500	0.500	0.500	0.500	0.467	0.394	0.500	0.500	0.557	0.344	0.470	0.470	0.500	0.500	0.500	0.500	0.544
Extra Trees	0.500	0.500	0.500	0.596	0.651	0.500	0.500	0.730	0.500	0.500	0.564	0.500	0.707	0.500	0.500	0.500	0.500	0.500	0.500	0.528	0.500	0.500	0.500	0.500	0.500	0.500	0.500	0.500	0.500	0.500
Gaussian Naive Bayes	0.669	0.806	0.780	0.596	0.736	0.750	0.653	0.918	0.706	0.702	0.762	0.651	0.819	0.755	0.623	0.500	0.832	0.652	0.705	0.725	0.812	0.813	0.684	0.844	0.625	0.656	0.686	0.780	0.576	0.622
Gradient Boosting	0.691	0.722	0.744	0.582	0.721	0.708	0.690	0.858	0.724	0.756	0.812	0.742	0.662	0.733	0.550	0.569	0.760	0.668	0.814	0.890	0.631	0.750	0.669	0.807	0.625	0.681	0.765	0.651	0.726	0.491
**Helstrom Quantum Classifier**	0.755	0.839	0.873	0.635	0.715	0.855	0.710	0.898	0.837	0.705	0.719	0.760	0.837	0.777	0.659	0.791	0.698	0.652	0.784	0.836	0.883	0.839	0.806	0.826	0.909	0.706	0.686	0.873	0.765	0.628
Linear Discriminant Analysis	0.612	0.670	0.780	0.596	0.676	0.688	0.671	0.877	0.706	0.677	0.719	0.651	0.819	0.795	0.567	0.600	0.832	0.547	0.595	0.697	0.812	0.781	0.569	0.794	0.500	0.543	0.653	0.817	0.676	0.622
Logistic Regression	0.598	0.670	0.780	0.596	0.676	0.688	0.688	0.877	0.651	0.677	0.683	0.651	0.819	0.773	0.567	0.600	0.832	0.567	0.595	0.670	0.669	0.781	0.584	0.794	0.500	0.543	0.653	0.835	0.594	0.622
Multi Layer Perceptron	0.727	0.767	0.873	0.561	0.676	0.708	0.746	0.775	0.726	0.677	0.748	0.878	0.742	0.773	0.606	0.706	0.691	0.547	0.830	0.810	0.740	0.626	0.784	0.844	0.610	0.660	0.698	0.762	0.726	0.522
Nearest Centroid	0.654	0.832	0.819	0.596	0.676	0.813	0.673	0.918	0.764	0.700	0.711	0.683	0.816	0.735	0.599	0.791	0.774	0.633	0.614	0.727	0.795	0.857	0.822	0.826	0.803	0.610	0.786	0.911	0.659	0.575
Nearest Neighbors	0.798	0.794	0.744	0.593	0.697	0.855	0.599	0.795	0.855	0.624	0.755	0.857	0.704	0.797	0.721	0.738	0.767	0.753	0.830	0.807	0.631	0.794	0.784	0.844	0.750	0.765	0.653	0.835	0.807	0.822
Passive Aggressive Classifier	0.492	0.827	0.714	0.593	0.479	0.542	0.510	0.612	0.161	0.621	0.624	0.822	0.753	0.757	0.508	0.500	0.767	0.612	0.549	0.567	0.414	0.563	0.791	0.626	0.500	0.551	0.786	0.802	0.789	0.644
Perceptron	0.548	0.365	0.429	0.596	0.490	0.813	0.728	0.898	0.688	0.598	0.620	0.497	0.641	0.838	0.495	0.428	0.722	0.570	0.739	0.500	0.571	0.850	0.569	0.826	0.500	0.665	0.698	0.911	0.500	0.606
Quadratic Discriminant Analysis	0.705	0.760	0.891	0.575	0.736	0.708	0.597	0.918	0.706	0.730	0.711	0.710	0.857	0.775	0.665	0.500	0.624	0.547	0.580	0.697	0.740	0.831	0.784	0.826	0.625	0.685	0.653	0.927	0.720	0.506
Random Forest	0.727	0.741	0.835	0.579	0.777	0.792	0.708	0.918	0.762	0.784	0.899	0.787	0.724	0.670	0.668	0.584	0.780	0.670	0.830	0.835	0.790	0.800	0.684	0.794	0.500	0.647	0.676	0.760	0.881	0.606
SVM - linear	0.683	0.689	0.835	0.575	0.676	0.688	0.635	0.857	0.726	0.702	0.769	0.689	0.798	0.755	0.606	0.500	0.832	0.610	0.580	0.725	0.829	0.831	0.500	0.844	0.500	0.601	0.686	0.853	0.676	0.622
SVM - poly	0.798	0.825	0.835	0.673	0.679	0.750	0.635	0.858	0.744	0.784	0.784	0.760	0.742	0.777	0.756	0.606	0.787	0.610	0.705	0.810	0.762	0.763	0.538	0.826	0.595	0.710	0.630	0.798	0.670	0.691
SVM - rbf	0.683	0.689	0.891	0.596	0.715	0.688	0.635	0.877	0.671	0.702	0.769	0.651	0.798	0.755	0.623	0.500	0.832	0.630	0.564	0.725	0.829	0.781	0.500	0.794	0.500	0.601	0.653	0.853	0.776	0.622

Table 4.29 | Balanced accuracy score for 30 energy image feature datasets for cell line MCF7

Classifiers	P1	P2	P3	P4	P5	P6	P7	P8	P9	P10	P11	P12	P13	P14	P15	P16	P17	P18	P19	P20	P21	P22	P23	P24	P25	P26	P27	P28	P29	P30
AdaBoost	0.812	1.000	0.964	0.774	0.756	0.897	0.835	0.980	0.964	0.806	0.699	0.822	0.875	0.878	0.837	0.669	0.878	0.778	0.970	0.944	0.829	1.000	0.669	0.863	0.500	0.921	0.898	0.946	0.876	0.875
Bernoulli Naive Bayes	0.783	0.677	0.837	0.771	0.792	0.690	0.839	0.920	0.837	0.807	0.856	0.630	0.683	0.860	0.816	0.500	0.865	0.695	0.500	0.868	0.779	0.500	0.500	0.652	0.500	0.626	0.864	0.575	0.565	0.753
Dummy Classifier	0.576	0.500	0.500	0.516	0.421	0.507	0.433	0.500	0.500	0.500	0.500	0.410	0.500	0.500	0.500	0.500	0.500	0.383	0.500	0.500	0.426	0.557	0.575	0.500	0.500	0.404	0.500	0.653	0.602	0.522
Extra Trees	0.500	0.500	0.500	0.763	0.538	0.500	0.500	0.855	0.500	0.500	0.500	0.500	0.500	0.500	0.500	0.500	0.545	0.500	0.500	0.500	0.500	0.500	0.500	0.500	0.500	0.500	0.500	0.500	0.500	0.500
Gaussian Naive Bayes	0.798	1.000	0.964	0.795	0.833	0.917	0.835	0.980	0.982	0.806	0.790	0.938	0.899	0.878	0.833	0.853	0.878	0.778	0.955	0.946	0.900	1.000	0.953	0.913	0.750	0.866	0.876	0.929	0.876	0.844
Gradient Boosting	0.742	1.000	0.964	0.777	0.816	0.855	0.835	0.980	0.817	0.892	0.699	0.861	0.742	0.837	0.795	0.769	0.787	0.737	0.955	0.944	0.829	1.000	0.953	0.844	0.595	0.832	0.898	0.891	0.863	0.806
**Helstrom Quantum Classifier**	0.848	0.942	0.946	0.792	0.736	0.875	0.929	0.980	0.893	0.811	0.870	0.917	0.798	0.838	0.837	0.853	0.923	0.800	0.909	0.920	0.867	0.907	0.838	0.907	0.985	0.925	0.876	0.817	0.907	0.797
Linear Discriminant Analysis	0.870	0.825	0.964	0.712	0.812	0.772	0.929	0.940	0.964	0.702	0.812	0.615	0.837	0.900	0.840	0.600	0.904	0.673	0.485	0.920	0.795	0.800	0.500	0.600	0.500	0.744	0.865	0.780	0.881	0.900
Logistic Regression	0.798	1.000	0.964	0.774	0.795	0.897	0.946	0.980	0.982	0.810	0.784	0.822	0.875	0.878	0.854	0.884	0.878	0.778	0.830	0.946	0.795	1.000	0.869	0.963	0.500	0.862	0.876	0.946	0.863	0.838
Multi Layer Perceptron	0.798	1.000	0.964	0.795	0.812	0.813	0.946	0.980	0.964	0.811	0.820	0.899	0.896	0.878	0.724	0.753	0.923	0.820	0.970	0.946	0.812	1.000	0.784	0.944	0.625	0.862	0.876	0.780	0.863	0.938
Nearest Centroid	0.804	0.885	0.911	0.712	0.812	0.920	0.893	0.920	0.893	0.725	0.870	0.641	0.774	0.900	0.833	0.922	0.904	0.800	0.864	0.839	0.850	0.926	0.906	0.889	0.879	0.900	0.830	0.837	0.889	0.797
Nearest Neighbors	0.877	0.962	0.964	0.816	0.875	0.833	0.893	0.960	0.780	0.756	0.848	0.822	0.837	0.797	0.665	0.869	0.942	0.755	0.939	0.946	0.883	0.881	0.900	0.876	0.750	0.916	0.865	0.762	0.963	0.906
Passive Aggressive Classifier	0.784	0.818	0.964	0.774	0.739	0.897	0.631	0.940	0.502	0.730	0.798	0.710	0.896	0.878	0.854	0.684	0.878	0.485	0.955	0.920	0.757	1.000	0.500	0.681	0.485	0.866	0.876	0.855	0.907	0.844
Perceptron	0.870	1.000	0.611	0.627	0.553	0.813	0.853	0.563	0.929	0.575	0.776	0.843	0.837	0.878	0.665	0.784	0.878	0.732	0.939	0.722	0.867	0.900	0.884	0.713	0.625	0.715	0.876	0.667	0.844	0.691
Quadratic Discriminant Analysis	0.798	1.000	0.964	0.795	0.833	0.958	0.835	0.980	0.964	0.806	0.755	0.938	0.840	0.878	0.833	0.853	0.878	0.798	0.830	0.946	0.900	1.000	1.000	0.913	0.750	0.891	0.898	0.929	0.894	0.844
Random Forest	0.770	1.000	0.964	0.795	0.756	0.897	0.853	0.980	0.798	0.890	0.792	0.861	0.917	0.878	0.857	0.569	0.878	0.715	0.970	0.944	0.900	0.950	0.700	0.863	0.500	0.891	0.898	0.909	0.900	0.922
SVM - linear	0.834	1.000	0.964	0.753	0.812	0.980	0.946	0.960	0.964	0.833	0.826	0.938	0.875	0.858	0.833	0.784	0.878	0.778	0.955	0.946	0.900	1.000	0.584	0.944	0.500	0.896	0.876	0.911	0.876	0.844
SVM - poly	0.848	1.000	0.964	0.792	0.736	0.897	0.835	0.940	0.911	0.811	0.870	0.857	0.854	0.838	0.833	0.853	0.923	0.757	0.814	0.946	0.883	1.000	0.800	0.944	0.610	0.832	0.876	0.873	0.863	0.891
SVM - rbf	0.834	1.000	0.964	0.753	0.812	0.980	0.946	0.960	0.964	0.833	0.790	0.938	0.875	0.858	0.833	0.784	0.878	0.778	0.955	0.946	0.900	1.000	0.584	0.944	0.500	0.925	0.909	0.929	0.826	0.844

Table 4.30 | Balanced accuracy score for 30 homogeneity image feature datasets for cell line MCF7

Classifiers	P1	P2	P3	P4	P5	P6	P7	P8	P9	P10	P11	P12	P13	P14	P15	P16	P17	P18	P19	P20	P21	P22	P23	P24	P25	P26	P27	P28	P29	P30
AdaBoost	0.848	0.981	0.964	0.774	0.812	0.855	0.891	0.980	0.891	0.917	0.776	0.938	0.875	0.838	0.854	0.869	0.878	0.758	0.830	0.890	0.883	0.981	0.969	0.963	0.500	0.891	0.909	0.911	0.900	0.938
Bernoulli Naive Bayes	0.848	0.981	0.964	0.792	0.812	0.817	0.929	0.980	0.946	0.778	0.806	0.766	0.857	0.838	0.854	0.638	0.885	0.758	0.769	0.946	0.900	0.844	0.500	0.944	0.500	0.950	0.886	0.911	0.870	0.838
Dummy Classifier	0.497	0.593	0.500	0.457	0.500	0.565	0.500	0.500	0.500	0.500	0.554	0.500	0.500	0.500	0.362	0.500	0.470	0.500	0.500	0.500	0.500	0.476	0.500	0.420	0.424	0.497	0.500	0.500	0.402	0.500
Extra Trees	0.571	0.500	0.500	0.777	0.707	0.500	0.760	0.897	0.500	0.553	0.585	0.500	0.595	0.625	0.630	0.500	0.935	0.500	0.500	0.751	0.648	0.500	0.500	0.500	0.500	0.500	0.521	0.500	0.500	0.500
Gaussian Naive Bayes	0.820	1.000	0.964	0.774	0.837	0.938	0.891	0.938	0.982	0.890	0.669	0.899	0.878	0.878	0.875	0.869	0.897	0.695	0.970	0.918	0.829	0.981	0.869	0.963	0.750	0.916	0.898	0.873	0.894	0.891
Gradient Boosting	0.820	1.000	0.964	0.795	0.854	0.980	0.891	0.980	0.946	0.944	0.727	0.784	0.801	0.838	0.816	0.669	0.923	0.758	0.970	0.835	0.740	0.981	0.900	0.913	0.500	0.921	0.932	0.946	0.894	0.953
**Helstrom Quantum Classifier**	0.848	0.981	0.964	0.753	0.756	0.897	0.946	0.897	0.871	0.890	0.790	0.938	0.857	0.838	0.875	0.938	0.878	0.758	0.955	0.890	0.900	0.981	0.953	0.963	0.970	0.950	0.932	0.873	0.863	0.859
Linear Discriminant Analysis	0.812	0.981	0.964	0.774	0.854	0.960	0.946	0.980	0.964	0.835	0.669	0.938	0.878	0.838	0.798	0.784	0.897	0.695	0.970	0.890	0.900	0.981	0.869	0.963	0.610	0.921	0.932	0.817	0.913	0.891
Logistic Regression	0.820	1.000	1.000	0.774	0.875	0.897	0.946	0.897	0.909	0.920	0.691	0.822	0.878	0.837	0.798	0.684	0.897	0.673	0.970	0.890	0.845	1.000	0.869	0.913	0.610	0.774	0.898	0.835	0.863	0.906
Multi Layer Perceptron	0.820	1.000	0.964	0.774	0.795	0.897	0.946	0.980	0.798	0.759	0.762	0.938	0.780	0.838	0.780	0.769	0.878	0.695	0.970	0.918	0.900	1.000	0.700	0.963	0.500	0.862	0.932	0.873	0.931	0.953
Nearest Centroid	0.870	0.923	0.964	0.753	0.812	0.960	0.946	0.980	0.946	0.806	0.790	0.899	0.875	0.838	0.854	0.953	0.885	0.758	0.939	0.811	0.900	0.981	0.922	0.963	0.909	0.921	0.886	0.911	0.876	0.859
Nearest Neighbors	0.842	0.981	0.909	0.753	0.812	0.855	0.946	0.980	0.891	0.730	0.820	0.920	0.763	0.858	0.724	0.669	0.858	0.717	0.970	0.946	0.900	0.931	0.700	0.963	0.625	0.950	0.898	0.817	0.963	0.938
Passive Aggressive Classifier	0.756	0.981	0.946	0.627	0.854	0.960	0.631	0.897	0.982	0.806	0.761	0.538	0.784	0.773	0.553	0.769	0.890	0.632	0.735	0.500	0.569	1.000	0.869	0.963	0.500	0.563	0.832	0.780	0.731	0.906
Perceptron	0.792	1.000	1.000	0.571	0.700	0.793	0.909	0.938	0.815	0.656	0.649	0.822	0.878	0.818	0.603	0.869	0.851	0.400	0.735	0.694	0.757	1.000	0.684	0.963	0.500	0.891	0.932	0.835	0.876	0.922
Quadratic Discriminant Analysis	0.856	1.000	0.964	0.736	0.875	0.938	0.891	0.938	0.964	0.918	0.669	0.938	0.840	0.878	0.837	0.869	0.878	0.735	0.970	0.890	0.900	0.981	0.800	0.963	0.625	0.891	0.898	0.817	0.913	0.791
Random Forest	0.877	1.000	0.964	0.774	0.857	0.813	0.909	0.980	0.853	0.839	0.770	0.861	0.801	0.838	0.896	0.769	0.878	0.715	0.970	0.944	0.883	0.981	0.600	0.963	0.500	0.862	0.898	0.946	0.900	0.953
SVM - linear	0.870	0.981	0.964	0.771	0.854	0.960	0.946	0.980	0.964	0.835	0.776	0.938	0.878	0.838	0.854	0.684	0.878	0.695	0.970	0.918	0.900	1.000	0.769	0.963	0.500	0.950	0.932	0.817	0.913	0.906
SVM - poly	0.870	1.000	0.964	0.771	0.837	0.980	0.946	0.980	0.780	0.839	0.762	0.938	0.917	0.838	0.854	0.969	0.878	0.695	0.970	0.946	0.900	1.000	0.584	0.963	0.625	0.862	0.898	0.817	0.913	0.922
SVM - rbf	0.848	1.000	0.964	0.812	0.854	0.960	0.946	0.980	0.964	0.917	0.705	0.938	0.878	0.838	0.854	0.784	0.878	0.737	0.970	0.918	0.900	0.981	0.769	0.963	0.500	0.832	0.932	0.873	0.913	0.906

Table 4.31 | AUROC score for 30 RGB image feature datasets for cell line MCF7

Classifiers	P1	P2	P3	P4	P5	P6	P7	P8	P9	P10	P11	P12	P13	P14	P15	P16	P17	P18	P19	P20	P21	P22	P23	P24	P25	P26	P27	P28	P29	P30
AdaBoost	0.921	1.000	0.944	0.920	0.941	1.000	0.944	0.938	0.982	0.972	0.957	0.962	0.979	0.940	0.941	0.700	1.000	0.980	1.000	1.000	0.929	1.000	0.784	1.000	0.720	0.971	0.977	0.927	1.000	1.000
Bernoulli Naive Bayes	0.863	1.000	0.944	0.881	0.958	1.000	0.944	0.938	0.964	0.972	0.907	1.000	0.979	1.000	0.941	0.969	0.942	0.980	0.985	1.000	0.929	1.000	0.869	1.000	0.955	0.946	0.977	0.800	0.913	0.984
Dummy Classifier	0.500	0.399	0.500	0.502	0.463	0.505	0.500	0.365	0.397	0.500	0.500	0.500	0.500	0.500	0.481	0.297	0.444	0.590	0.500	0.428	0.602	0.439	0.500	0.500	0.500	0.572	0.339	0.431	0.500	0.500
Extra Trees	0.679	1.000	0.833	0.902	0.923	0.833	0.889	0.938	0.944	0.946	0.929	0.923	0.979	0.958	0.941	0.600	0.955	0.938	1.000	1.000	0.929	0.850	0.500	0.950	0.500	0.912	0.944	0.556	0.731	0.700
Gaussian Naive Bayes	0.863	1.000	0.944	0.902	0.920	1.000	0.944	0.938	0.944	0.972	0.907	0.979	0.979	0.980	0.941	0.900	0.942	0.960	1.000	1.000	0.895	0.950	0.884	1.000	0.985	0.971	0.955	0.835	0.913	0.900
Gradient Boosting	0.921	1.000	0.833	0.878	0.902	0.875	0.944	0.855	0.927	0.944	0.943	0.962	0.979	0.940	0.941	0.700	1.000	0.500	1.000	1.000	1.000	1.000	0.769	1.000	0.720	0.941	1.000	0.871	1.000	1.000
**Helstrom Quantum Classifier**	0.957	1.000	0.944	0.920	0.899	1.000	0.944	0.980	1.000	0.946	0.964	1.000	0.941	1.000	0.941	0.969	0.981	0.938	1.000	1.000	0.912	1.000	0.900	1.000	0.939	0.946	0.888	0.944	1.000	0.800
Linear Discriminant Analysis	0.921	1.000	0.944	0.920	0.920	0.958	0.889	0.938	1.000	0.944	0.964	1.000	0.979	1.000	0.941	1.000	0.955	0.938	1.000	1.000	0.929	0.900	0.784	0.950	0.970	0.946	0.933	0.889	1.000	0.900
Logistic Regression	0.885	1.000	0.889	0.941	0.923	0.875	0.889	0.938	1.000	0.944	1.000	1.000	0.979	1.000	0.979	0.800	1.000	0.938	1.000	1.000	0.929	1.000	0.784	1.000	0.845	0.946	1.000	0.909	1.000	0.900
Multi Layer Perceptron	0.957	1.000	0.833	0.941	0.920	0.917	0.889	0.938	1.000	0.944	0.978	0.962	0.979	1.000	0.979	1.000	1.000	0.898	1.000	1.000	0.929	1.000	0.784	1.000	0.860	0.975	1.000	0.909	1.000	1.000
Nearest Neighbors	0.921	1.000	0.889	0.979	0.938	0.875	0.927	0.938	0.927	0.974	0.964	1.000	0.941	1.000	0.941	0.984	0.981	0.938	1.000	1.000	0.895	0.900	0.884	1.000	0.845	0.971	0.967	0.982	1.000	0.884
Quadratic Discriminant Analysis	0.921	1.000	1.000	0.941	0.902	0.958	0.944	0.938	0.982	0.944	0.978	1.000	0.979	0.960	0.941	1.000	0.962	0.960	0.985	1.000	1.000	1.000	0.884	1.000	0.750	0.946	0.955	0.944	0.981	1.000
Random Forest	0.885	1.000	0.833	0.917	0.941	1.000	0.944	0.938	0.982	0.972	0.943	1.000	0.979	0.940	0.941	0.700	1.000	0.980	1.000	1.000	0.929	1.000	0.869	1.000	0.845	0.971	0.977	0.982	1.000	1.000
SVM - linear	0.885	1.000	0.944	0.941	0.920	0.875	0.889	0.980	1.000	0.944	0.978	1.000	0.979	0.980	0.979	0.900	0.955	0.938	1.000	1.000	0.912	0.900	0.784	1.000	0.845	0.975	1.000	0.964	1.000	0.900
SVM - poly	0.863	1.000	0.927	0.938	0.920	0.855	0.889	0.938	0.944	0.918	0.964	0.962	0.692	0.708	0.941	0.884	1.000	0.772	1.000	0.806	0.929	0.650	0.700	0.900	0.860	0.946	0.967	0.927	0.900	0.900
SVM - rbf	0.885	1.000	0.556	0.899	0.920	0.875	0.889	0.980	1.000	0.944	0.978	1.000	0.615	0.667	0.979	0.900	1.000	0.792	1.000	0.778	0.857	1.000	0.784	0.600	0.500	0.975	0.977	0.982	0.931	0.900

Results exclude Nearest Centroid, Passive Aggressive Classifier and Perceptron.

Table 4.32 | AUROC score for 30 L*u*v* image feature datasets for cell line MCF7

Classifiers	P1	P2	P3	P4	P5	P6	P7	P8	P9	P10	P11	P12	P13	P14	P15	P16	P17	P18	P19	P20	P21	P22	P23	P24	P25	P26	P27	P28	P29	P30
AdaBoost	0.885	1.000	1.000	0.938	0.941	0.917	0.944	0.980	0.982	0.972	0.964	0.962	0.979	0.940	0.941	0.700	1.000	0.898	1.000	1.000	0.929	1.000	0.869	1.000	0.970	0.946	0.977	0.944	0.981	0.969
Bernoulli Naive Bayes	0.935	1.000	1.000	0.861	0.938	0.980	0.982	0.980	0.964	0.944	0.921	1.000	0.979	0.940	0.979	0.969	0.942	0.940	0.970	1.000	0.967	1.000	0.969	0.981	0.924	0.975	0.955	0.982	0.981	0.953
Dummy Classifier	0.519	0.500	0.500	0.500	0.500	0.443	0.526	0.500	0.500	0.500	0.497	0.389	0.306	0.500	0.500	0.500	0.451	0.500	0.708	0.500	0.500	0.563	0.500	0.739	0.500	0.497	0.500	0.500	0.539	0.500
Extra Trees	0.921	1.000	0.944	0.899	0.941	1.000	0.944	0.938	1.000	0.946	0.943	1.000	0.979	1.000	0.979	0.900	0.962	0.960	1.000	1.000	0.983	0.850	0.500	1.000	0.500	0.946	0.944	0.667	0.981	0.900
Gaussian Naive Bayes	0.899	1.000	1.000	0.861	0.979	1.000	1.000	0.938	0.982	0.944	0.943	1.000	0.979	0.940	0.979	1.000	0.942	0.940	0.985	1.000	0.983	1.000	0.984	1.000	0.985	0.946	0.955	0.927	0.981	0.984
Gradient Boosting	0.863	1.000	0.944	0.920	0.902	0.917	0.944	0.855	0.944	0.972	0.929	1.000	0.979	0.898	0.979	1.000	1.000	0.960	1.000	1.000	1.000	1.000	0.969	1.000	0.720	0.946	0.977	0.944	1.000	0.984
**Helstrom Quantum Classifier**	0.935	1.000	1.000	0.878	0.958	1.000	0.944	0.980	0.982	0.918	1.000	0.979	0.979	0.960	0.979	0.984	1.000	0.940	0.985	1.000	0.895	1.000	0.884	1.000	0.879	0.971	0.911	0.982	0.981	0.900
Linear Discriminant Analysis	0.921	1.000	0.944	0.920	0.979	1.000	0.889	0.938	1.000	0.944	1.000	1.000	0.979	0.960	0.941	1.000	1.000	0.960	1.000	1.000	0.929	1.000	0.784	1.000	0.970	0.946	1.000	0.944	0.981	0.900
Logistic Regression	0.885	1.000	0.944	0.941	0.923	0.875	0.889	0.938	1.000	0.918	0.964	1.000	0.979	0.960	0.979	0.900	1.000	0.898	1.000	1.000	0.929	1.000	0.784	1.000	0.860	0.946	1.000	0.982	1.000	1.000
Multi Layer Perceptron	0.877	1.000	1.000	0.941	0.923	0.875	0.889	0.938	0.982	0.918	0.964	1.000	0.979	0.940	0.979	0.884	1.000	0.940	1.000	1.000	0.929	1.000	0.784	1.000	1.000	0.946	1.000	0.853	0.981	0.984
Nearest Neighbors	0.957	1.000	1.000	0.878	0.920	0.917	0.889	0.980	0.927	0.918	1.000	1.000	0.979	0.960	0.941	0.984	0.981	0.918	0.985	1.000	0.912	1.000	0.800	1.000	0.750	0.946	1.000	0.982	1.000	0.884
Quadratic Discriminant Analysis	0.885	1.000	1.000	0.941	0.902	0.958	0.944	0.938	0.964	0.944	1.000	1.000	0.979	0.960	0.941	0.900	0.981	0.918	0.985	1.000	0.929	1.000	0.884	1.000	1.000	0.946	0.955	1.000	0.981	0.984
Random Forest	0.885	1.000	0.944	0.938	0.941	0.958	0.889	0.938	0.982	0.944	0.907	1.000	0.979	0.940	0.941	0.700	1.000	0.960	1.000	1.000	0.929	1.000	0.984	1.000	0.985	0.946	0.977	0.944	0.981	0.984
SVM - linear	0.921	1.000	1.000	0.920	0.920	0.917	1.000	0.980	1.000	0.918	1.000	1.000	0.979	0.940	0.941	1.000	0.981	0.898	0.985	1.000	0.929	1.000	0.784	1.000	0.845	0.946	1.000	1.000	1.000	1.000
SVM - poly	0.885	1.000	1.000	0.899	0.941	0.875	0.889	0.980	0.982	0.918	0.964	1.000	0.902	0.958	0.941	0.800	0.909	0.938	0.985	1.000	0.929	1.000	0.784	0.950	0.860	0.946	0.900	0.778	1.000	0.884
SVM - rbf	0.821	1.000	0.722	0.920	0.958	0.917	0.889	0.980	0.982	0.944	1.000	1.000	0.979	0.960	0.979	1.000	0.864	0.940	0.500	1.000	0.929	1.000	0.500	0.550	0.500	0.946	0.833	1.000	0.650	0.500

Results exclude Nearest Centroid, Passive Aggressive Classifier and Perceptron.

Table 4.33 | AUROC score for 30 contrast image feature datasets for cell line MCF7																														
**Classifiers**	**P1**	**P2**	**P3**	**P4**	**P5**	**P6**	**P7**	**P8**	**P9**	**P10**	**P11**	**P12**	**P13**	**P14**	**P15**	**P16**	**P17**	**P18**	**P19**	**P20**	**P21**	**P22**	**P23**	**P24**	**P25**	**P26**	**P27**	**P28**	**P29**	**P30**
AdaBoost	0.834	0.981	0.964	0.697	0.700	0.897	0.946	0.920	0.800	0.861	0.655	0.878	0.854	0.753	0.721	0.769	0.885	0.713	0.955	0.893	0.812	0.944	0.669	0.826	0.500	0.715	0.820	0.891	0.881	0.906
Bernoulli Naive Bayes	0.762	0.890	0.964	0.686	0.739	0.835	0.853	0.855	0.722	0.706	0.699	0.745	0.665	0.650	0.591	0.569	0.741	0.423	0.720	0.614	0.560	0.844	0.500	0.813	0.500	0.715	0.786	0.744	0.807	0.822
Dummy Classifier	0.500	0.500	0.500	0.561	0.500	0.507	0.500	0.468	0.500	0.520	0.500	0.603	0.500	0.500	0.582	0.406	0.500	0.500	0.455	0.500	0.500	0.520	0.500	0.520	0.500	0.500	0.500	0.577	0.396	0.500
Extra Trees	0.500	0.500	0.500	0.641	0.500	0.500	0.500	0.500	0.500	0.500	0.500	0.500	0.500	0.500	0.500	0.500	0.500	0.500	0.500	0.500	0.500	0.500	0.500	0.500	0.500	0.500	0.500	0.500	0.500	0.500
Gaussian Naive Bayes	0.778	0.799	0.760	0.668	0.627	0.625	0.760	0.750	0.611	0.632	0.542	0.612	0.591	0.565	0.686	0.500	0.598	0.503	0.735	0.532	0.417	0.750	0.584	0.631	0.610	0.538	0.776	0.722	0.631	0.684
Gradient Boosting	0.792	1.000	0.891	0.620	0.700	0.897	0.817	0.898	0.500	0.500	0.691	0.861	0.724	0.775	0.784	0.853	0.832	0.627	0.955	0.890	0.812	0.663	0.669	0.807	0.610	0.715	0.786	0.835	0.844	0.838
**Helstrom Quantum Classifier**	0.820	0.981	0.871	0.694	0.609	0.857	0.891	0.917	0.722	0.759	0.848	0.861	0.627	0.757	0.686	0.584	0.897	0.445	0.595	0.725	0.779	0.700	0.722	0.650	0.955	0.681	0.686	0.798	0.731	0.744
Linear Discriminant Analysis	0.756	0.708	0.631	0.707	0.588	0.627	0.760	0.792	0.611	0.550	0.562	0.612	0.591	0.565	0.532	0.500	0.500	0.442	0.735	0.531	0.526	0.750	0.584	0.650	0.610	0.538	0.776	0.760	0.731	0.569
Logistic Regression	0.756	0.818	0.687	0.686	0.683	0.668	0.760	0.833	0.667	0.550	0.699	0.612	0.647	0.565	0.532	0.500	0.500	0.462	0.735	0.531	0.510	0.731	0.484	0.631	0.485	0.568	0.776	0.760	0.713	0.569
Multi Layer Perceptron	0.820	0.981	0.982	0.543	0.683	0.813	0.891	0.833	0.667	0.810	0.719	0.920	0.745	0.628	0.822	0.553	0.897	0.588	0.610	0.892	0.795	0.894	0.800	0.644	0.625	0.803	0.786	0.817	0.731	0.822
Nearest Neighbors	0.877	0.916	0.815	0.795	0.659	0.793	0.837	0.918	0.631	0.785	0.891	0.899	0.745	0.673	0.647	0.569	0.962	0.525	0.720	0.784	0.740	0.813	0.584	0.663	0.625	0.832	0.786	0.762	0.944	0.822
Quadratic Discriminant Analysis	0.814	0.818	0.815	0.707	0.627	0.667	0.704	0.708	0.611	0.548	0.534	0.591	0.591	0.607	0.571	0.500	0.624	0.462	0.735	0.560	0.488	0.731	0.584	0.650	0.610	0.538	0.776	0.704	0.681	0.584
Random Forest	0.842	1.000	0.964	0.736	0.718	0.897	0.873	0.918	0.798	0.756	0.820	0.920	0.742	0.858	0.801	0.569	0.878	0.650	0.970	0.918	0.812	0.794	0.600	0.813	0.500	0.857	0.786	0.762	0.931	0.838
SVM - linear	0.699	0.909	0.853	0.707	0.721	0.585	0.853	0.897	0.611	0.523	0.649	0.553	0.630	0.565	0.434	0.500	0.500	0.462	0.735	0.529	0.433	0.650	0.500	0.631	0.500	0.538	0.776	0.780	0.731	0.600
SVM - poly	0.727	0.890	0.909	0.784	0.721	0.813	0.911	0.792	0.611	0.601	0.834	0.840	0.780	0.648	0.784	0.500	0.722	0.420	0.610	0.838	0.669	0.550	0.553	0.650	0.500	0.710	0.776	0.780	0.731	0.722
SVM - rbf	0.770	0.500	0.538	0.707	0.718	0.793	0.760	0.500	0.611	0.654	0.500	0.500	0.609	0.565	0.490	0.500	0.533	0.462	0.860	0.529	0.500	0.550	0.500	0.631	0.500	0.504	0.500	0.762	0.531	0.500

Results exclude Nearest Centroid, Passive Aggressive Classifier and Perceptron.

Table 4.34 | AUROC score for 30 correlation image feature datasets for cell line MCF7																														
**Classifiers**	**P1**	**P2**	**P3**	**P4**	**P5**	**P6**	**P7**	**P8**	**P9**	**P10**	**P11**	**P12**	**P13**	**P14**	**P15**	**P16**	**P17**	**P18**	**P19**	**P20**	**P21**	**P22**	**P23**	**P24**	**P25**	**P26**	**P27**	**P28**	**P29**	**P30**
AdaBoost	0.713	0.708	0.800	0.596	0.718	0.625	0.690	0.857	0.724	0.811	0.806	0.671	0.795	0.733	0.704	0.469	0.832	0.690	0.705	0.864	0.724	0.831	0.469	0.844	0.500	0.626	0.686	0.722	0.613	0.591
Bernoulli Naive Bayes	0.661	0.572	0.633	0.596	0.676	0.667	0.673	0.878	0.671	0.779	0.756	0.612	0.798	0.757	0.683	0.500	0.657	0.592	0.549	0.754	0.740	0.757	0.500	0.752	0.500	0.631	0.830	0.633	0.402	0.622
Dummy Classifier	0.500	0.586	0.500	0.537	0.543	0.525	0.433	0.343	0.500	0.500	0.547	0.500	0.484	0.547	0.466	0.606	0.540	0.500	0.500	0.513	0.500	0.539	0.500	0.500	0.500	0.500	0.586	0.393	0.500	0.500
Extra Trees	0.500	0.500	0.500	0.537	0.630	0.500	0.500	0.667	0.500	0.500	0.500	0.500	0.500	0.500	0.500	0.500	0.500	0.500	0.500	0.500	0.500	0.500	0.500	0.500	0.500	0.500	0.500	0.500	0.500	0.500
Gaussian Naive Bayes	0.669	0.806	0.780	0.596	0.736	0.750	0.653	0.918	0.706	0.702	0.762	0.651	0.819	0.755	0.623	0.500	0.832	0.652	0.705	0.725	0.812	0.813	0.684	0.844	0.625	0.656	0.686	0.780	0.576	0.622
Gradient Boosting	0.727	0.832	0.891	0.617	0.756	0.750	0.746	0.857	0.724	0.730	0.812	0.721	0.641	0.733	0.644	0.453	0.696	0.650	0.705	0.890	0.790	0.750	0.669	0.794	0.625	0.676	0.676	0.651	0.744	0.606
**Helstrom Quantum Classifier**	0.790	0.806	0.873	0.635	0.662	0.750	0.673	0.898	0.837	0.759	0.733	0.760	0.816	0.735	0.724	0.859	0.677	0.590	0.814	0.808	0.812	0.894	0.738	0.813	0.955	0.676	0.653	0.873	0.733	0.628
Linear Discriminant Analysis	0.612	0.670	0.780	0.596	0.676	0.688	0.671	0.877	0.706	0.677	0.719	0.651	0.819	0.795	0.567	0.600	0.832	0.547	0.595	0.697	0.812	0.781	0.569	0.794	0.500	0.543	0.653	0.817	0.676	0.622
Logistic Regression	0.598	0.670	0.780	0.596	0.676	0.688	0.688	0.877	0.651	0.677	0.683	0.651	0.819	0.773	0.567	0.600	0.832	0.567	0.595	0.670	0.669	0.731	0.584	0.794	0.500	0.543	0.620	0.853	0.594	0.622
Multi Layer Perceptron	0.762	0.806	0.891	0.620	0.659	0.750	0.673	0.837	0.651	0.705	0.705	0.819	0.742	0.715	0.571	0.500	0.710	0.483	0.580	0.863	0.581	0.731	0.784	0.844	0.625	0.719	0.686	0.780	0.600	0.622
Nearest Neighbors	0.870	0.794	0.744	0.593	0.697	0.750	0.599	0.795	0.855	0.757	0.804	0.784	0.704	0.797	0.721	0.784	0.722	0.673	0.845	0.807	0.900	0.794	0.700	0.844	0.500	0.765	0.653	0.835	0.739	0.706
Quadratic Discriminant Analysis	0.705	0.760	0.891	0.575	0.736	0.708	0.597	0.918	0.706	0.730	0.711	0.710	0.857	0.775	0.665	0.500	0.624	0.547	0.580	0.697	0.740	0.831	0.784	0.826	0.625	0.685	0.653	0.927	0.720	0.506
Random Forest	0.806	0.760	0.835	0.599	0.739	0.708	0.690	0.918	0.817	0.784	0.755	0.748	0.683	0.755	0.571	0.584	0.787	0.608	0.845	0.835	0.774	0.800	0.584	0.794	0.500	0.735	0.742	0.760	0.881	0.606
SVM - linear	0.683	0.689	0.780	0.575	0.676	0.688	0.653	0.857	0.726	0.702	0.769	0.651	0.819	0.755	0.606	0.500	0.832	0.610	0.580	0.725	0.829	0.781	0.500	0.844	0.500	0.601	0.653	0.909	0.500	0.622
SVM - poly	0.620	0.825	0.853	0.593	0.638	0.750	0.599	0.858	0.651	0.784	0.784	0.538	0.742	0.733	0.647	0.500	0.787	0.442	0.705	0.810	0.833	0.731	0.538	0.826	0.595	0.647	0.630	0.611	0.670	0.522
SVM - rbf	0.683	0.689	0.500	0.575	0.500	0.688	0.635	0.500	0.500	0.674	0.769	0.500	0.500	0.817	0.641	0.500	0.500	0.500	0.564	0.725	0.500	0.500	0.500	0.794	0.500	0.601	0.500	0.909	0.776	0.500

Results exclude Nearest Centroid, Passive Aggressive Classifier and Perceptron.

Table 4.35 | AUROC score for 30 energy image feature datasets for cell line MCF7																														
**Classifiers**	**P1**	**P2**	**P3**	**P4**	**P5**	**P6**	**P7**	**P8**	**P9**	**P10**	**P11**	**P12**	**P13**	**P14**	**P15**	**P16**	**P17**	**P18**	**P19**	**P20**	**P21**	**P22**	**P23**	**P24**	**P25**	**P26**	**P27**	**P28**	**P29**	**P30**
AdaBoost	0.812	1.000	0.964	0.774	0.756	0.897	0.835	0.980	0.964	0.864	0.699	0.861	0.833	0.878	0.780	0.700	0.760	0.778	0.845	0.944	0.829	1.000	0.769	0.881	0.500	0.862	0.898	0.891	0.900	0.891
Bernoulli Naive Bayes	0.783	0.677	0.837	0.771	0.792	0.690	0.839	0.920	0.837	0.807	0.856	0.630	0.683	0.860	0.816	0.500	0.865	0.695	0.500	0.868	0.779	0.500	0.500	0.652	0.500	0.626	0.864	0.575	0.565	0.753
Dummy Classifier	0.500	0.437	0.542	0.500	0.505	0.500	0.635	0.612	0.395	0.500	0.500	0.470	0.500	0.385	0.564	0.500	0.502	0.500	0.500	0.402	0.707	0.500	0.422	0.415	0.500	0.500	0.395	0.500	0.500	0.500
Extra Trees	0.500	0.500	0.500	0.739	0.538	0.500	0.500	0.500	0.500	0.500	0.500	0.500	0.500	0.500	0.500	0.500	0.500	0.500	0.500	0.500	0.500	0.500	0.500	0.500	0.500	0.500	0.500	0.500	0.500	0.500
Gaussian Naive Bayes	0.798	1.000	0.964	0.795	0.833	0.917	0.835	0.980	0.982	0.806	0.790	0.938	0.899	0.878	0.833	0.853	0.878	0.778	0.955	0.946	0.900	1.000	0.953	0.913	0.750	0.866	0.876	0.929	0.876	0.844
Gradient Boosting	0.742	0.955	0.853	0.774	0.816	0.813	0.853	0.813	0.835	0.836	0.627	0.822	0.700	0.795	0.739	0.669	0.806	0.757	0.955	0.944	0.757	1.000	0.884	0.844	0.610	0.832	0.898	0.891	0.844	0.922
**Helstrom Quantum Classifier**	0.826	0.923	0.946	0.753	0.574	0.792	0.911	0.920	0.893	0.779	0.848	0.941	0.854	0.880	0.875	0.953	0.923	0.800	0.909	0.920	0.867	0.781	0.884	0.926	0.924	0.950	0.876	0.873	0.876	0.797
Linear Discriminant Analysis	0.870	0.825	0.964	0.712	0.812	0.772	0.929	0.940	0.964	0.702	0.812	0.615	0.837	0.900	0.840	0.600	0.904	0.673	0.485	0.920	0.795	0.800	0.500	0.550	0.500	0.744	0.865	0.780	0.881	0.900
Logistic Regression	0.798	1.000	0.964	0.795	0.756	0.897	0.946	0.980	0.982	0.889	0.784	0.822	0.875	0.898	0.854	0.884	0.878	0.778	0.830	0.946	0.795	0.950	0.500	0.863	0.500	0.891	0.898	0.946	0.863	0.838
Multi Layer Perceptron	0.798	1.000	0.964	0.756	0.736	0.813	0.946	0.960	0.760	0.890	0.842	0.822	0.875	0.878	0.724	0.753	0.897	0.715	0.830	0.863	0.812	1.000	0.769	0.863	0.750	0.891	0.876	0.946	0.931	0.838
Nearest Neighbors	0.848	0.962	0.853	0.771	0.812	0.833	0.893	0.960	0.835	0.782	0.848	0.979	0.837	0.797	0.816	0.869	0.942	0.715	0.955	0.946	0.883	0.881	0.900	0.876	0.625	0.941	0.865	0.762	0.963	0.906
Quadratic Discriminant Analysis	0.798	1.000	0.964	0.795	0.833	0.958	0.835	0.980	0.964	0.806	0.755	0.938	0.840	0.878	0.833	0.853	0.878	0.798	0.830	0.946	0.900	1.000	1.000	0.913	0.750	0.891	0.898	0.929	0.894	0.844
Random Forest	0.784	1.000	0.964	0.795	0.777	0.855	0.853	0.980	0.760	0.892	0.685	0.861	0.878	0.898	0.840	0.569	0.878	0.652	0.970	0.972	0.900	0.950	0.500	0.863	0.500	0.891	0.898	0.946	0.900	0.938
SVM - linear	0.834	1.000	0.964	0.753	0.812	0.980	0.946	0.940	0.964	0.833	0.812	0.938	0.833	0.858	0.833	0.500	0.923	0.778	0.955	0.946	0.867	0.850	0.584	0.944	0.500	0.774	0.876	0.946	0.876	0.844
SVM - poly	0.891	1.000	0.964	0.753	0.736	0.897	0.929	0.940	0.911	0.811	0.870	0.857	0.854	0.838	0.833	0.500	0.897	0.757	0.955	0.946	0.867	0.800	0.669	0.813	0.610	0.921	0.876	0.780	0.863	0.891
SVM - rbf	0.848	1.000	0.964	0.753	0.812	0.980	0.835	0.940	0.964	0.833	0.790	0.938	0.833	0.858	0.833	0.500	0.500	0.778	0.955	0.946	0.867	1.000	0.584	0.944	0.500	0.925	0.876	0.929	0.826	0.844

Results exclude Nearest Centroid, Passive Aggressive Classifier and Perceptron.

Table 4.36 | AUROC score for 30 homogeneity image feature datasets for cell line MCF7																														
**Classifiers**	**P1**	**P2**	**P3**	**P4**	**P5**	**P6**	**P7**	**P8**	**P9**	**P10**	**P11**	**P12**	**P13**	**P14**	**P15**	**P16**	**P17**	**P18**	**P19**	**P20**	**P21**	**P22**	**P23**	**P24**	**P25**	**P26**	**P27**	**P28**	**P29**	**P30**
AdaBoost	0.848	0.981	0.964	0.774	0.854	0.772	0.891	0.980	0.891	0.890	0.792	0.843	0.837	0.858	0.739	0.684	0.832	0.758	0.845	0.944	0.829	0.981	0.969	0.963	0.500	0.921	0.909	0.835	0.900	0.938
Bernoulli Naive Bayes	0.848	0.981	0.964	0.792	0.812	0.817	0.929	0.980	0.946	0.778	0.806	0.766	0.857	0.838	0.854	0.638	0.885	0.758	0.769	0.946	0.900	0.844	0.500	0.944	0.500	0.950	0.886	0.911	0.870	0.838
Dummy Classifier	0.500	0.500	0.500	0.540	0.558	0.482	0.399	0.385	0.615	0.500	0.500	0.500	0.484	0.500	0.442	0.453	0.500	0.500	0.500	0.539	0.500	0.500	0.500	0.500	0.470	0.531	0.474	0.562	0.500	0.344
Extra Trees	0.500	0.500	0.500	0.816	0.651	0.500	0.500	0.500	0.500	0.500	0.536	0.500	0.500	0.647	0.556	0.500	0.500	0.500	0.500	0.500	0.500	0.500	0.500	0.500	0.500	0.500	0.598	0.500	0.500	0.500
Gaussian Naive Bayes	0.820	1.000	0.964	0.774	0.837	0.938	0.891	0.938	0.982	0.890	0.669	0.899	0.878	0.878	0.875	0.869	0.897	0.695	0.970	0.918	0.829	0.981	0.869	0.963	0.750	0.916	0.898	0.873	0.894	0.891
Gradient Boosting	0.786	1.000	0.964	0.736	0.854	0.813	0.891	0.938	0.780	0.864	0.727	0.881	0.784	0.858	0.683	0.769	0.741	0.757	0.970	0.835	0.500	0.981	1.000	0.913	0.610	0.774	0.909	0.946	0.881	0.969
**Helstrom Quantum Classifier**	0.870	0.981	0.964	0.736	0.718	0.855	0.946	0.897	0.946	0.835	0.812	0.938	0.780	0.838	0.875	0.853	0.839	0.758	0.955	0.946	0.900	0.944	0.953	0.913	0.955	0.950	0.932	0.873	0.900	0.828
Linear Discriminant Analysis	0.812	0.981	0.964	0.774	0.854	0.960	0.946	0.980	0.964	0.835	0.669	0.938	0.878	0.838	0.798	0.784	0.897	0.695	0.970	0.890	0.900	0.981	0.869	0.963	0.610	0.921	0.932	0.817	0.913	0.891
Logistic Regression	0.820	1.000	1.000	0.774	0.875	0.897	0.909	0.897	0.909	0.920	0.691	0.822	0.878	0.837	0.798	0.684	0.897	0.673	0.970	0.890	0.845	1.000	0.769	0.913	0.500	0.774	0.898	0.835	0.863	0.906
Multi Layer Perceptron	0.820	1.000	0.964	0.774	0.795	0.855	0.909	0.980	0.982	0.864	0.727	0.822	0.840	0.837	0.917	0.869	0.916	0.695	0.970	0.920	0.917	1.000	0.784	0.963	0.500	0.891	0.898	0.835	0.863	0.953
Nearest Neighbors	0.856	0.981	0.909	0.753	0.812	0.855	0.946	0.980	0.780	0.836	0.776	0.920	0.784	0.858	0.798	0.669	0.858	0.717	0.970	0.946	0.900	1.000	0.700	0.963	0.500	0.950	0.898	0.817	0.963	0.806
Quadratic Discriminant Analysis	0.856	1.000	0.964	0.736	0.875	0.938	0.891	0.938	0.964	0.918	0.669	0.938	0.840	0.878	0.837	0.869	0.878	0.735	0.970	0.890	0.900	0.981	0.800	0.963	0.625	0.891	0.898	0.817	0.913	0.791
Random Forest	0.820	1.000	0.964	0.816	0.837	0.772	0.909	0.980	0.853	0.839	0.842	0.861	0.801	0.838	0.763	0.769	0.878	0.673	0.970	0.944	0.883	0.981	0.700	0.963	0.500	0.862	0.898	0.817	0.900	0.938
SVM - linear	0.848	0.981	0.964	0.771	0.854	0.960	0.946	0.980	0.964	0.835	0.776	0.938	0.878	0.838	0.875	0.584	0.878	0.695	0.970	0.918	0.900	0.981	0.500	0.963	0.500	0.832	0.932	0.873	0.913	0.906
SVM - poly	0.870	0.981	0.964	0.792	0.812	0.897	0.946	0.980	0.964	0.811	0.798	0.938	0.917	0.627	0.854	0.969	0.916	0.695	0.970	0.920	0.900	1.000	0.584	0.963	0.500	0.862	0.932	0.873	0.881	0.853
SVM - rbf	0.500	0.981	0.500	0.812	0.854	0.500	0.946	0.980	0.964	0.500	0.705	0.938	0.500	0.838	0.500	0.500	0.897	0.500	0.500	0.918	0.900	0.500	0.684	0.500	0.500	0.832	0.932	0.500	0.913	0.906

Results exclude Nearest Centroid, Passive Aggressive Classifier and Perceptron.

Table 4.37 | Balanced accuracy score for 30 RGB image feature datasets for cell line U251

Classifiers	P1	P2	P3	P4	P5	P6	P7	P8	P9	P10	P11	P12	P13	P14	P15	P16	P17	P18	P19	P20	P21	P22	P23	P24	P25	P26	P27	P28	P29	P30
AdaBoost	0.917	0.925	0.946	0.955	0.900	0.943	0.839	0.921	0.962	1.000	0.900	0.929	0.921	0.832	0.923	0.871	0.917	0.927	0.893	0.907	0.900	0.920	1.000	0.938	0.955	0.912	0.929	0.786	0.795	0.871
Bernoulli Naive Bayes	0.917	0.875	0.944	0.955	0.933	0.913	0.895	0.886	0.955	0.866	0.825	0.929	0.906	0.932	0.923	0.846	0.854	0.927	0.867	0.857	0.850	0.868	0.921	0.858	0.955	0.840	0.946	0.757	0.833	0.871
Dummy Classifier	0.500	0.582	0.500	0.500	0.642	0.441	0.500	0.662	0.500	0.644	0.500	0.421	0.500	0.405	0.546	0.500	0.526	0.397	0.598	0.500	0.500	0.401	0.500	0.505	0.437	0.500	0.500	0.400	0.500	0.500
Extra Trees	0.875	0.875	0.889	0.955	0.800	0.978	0.918	0.898	0.955	0.891	0.950	0.929	0.945	0.955	0.923	0.812	0.896	0.778	0.763	0.821	0.800	0.895	0.933	0.667	0.682	0.643	0.500	0.500	0.750	0.778
Gaussian Naive Bayes	0.897	0.925	0.944	0.935	0.967	0.957	0.895	0.955	0.935	1.000	0.900	0.929	0.945	0.921	0.923	0.871	0.896	0.927	0.841	0.857	0.850	0.947	0.921	0.938	0.955	0.824	0.944	0.769	0.792	0.871
Gradient Boosting	0.917	0.925	0.946	0.955	1.000	0.943	0.921	0.888	0.981	1.000	0.887	0.929	0.897	0.977	0.902	0.896	0.878	0.927	0.893	0.929	0.831	0.920	0.967	0.938	0.955	0.912	0.929	0.769	0.835	0.871
**Helstrom Quantum Classifier**	0.917	0.950	0.944	1.000	0.933	0.935	0.947	0.932	0.981	0.950	0.875	0.929	0.906	0.955	0.923	0.900	0.854	0.871	0.893	0.929	0.850	0.921	0.944	0.960	0.955	0.807	0.944	0.807	0.917	0.871
Linear Discriminant Analysis	0.917	0.875	0.861	0.955	0.867	0.935	0.947	0.932	0.981	0.925	0.841	0.929	0.906	0.955	0.923	0.900	0.816	0.889	0.842	0.800	0.800	0.947	0.967	0.960	0.955	0.786	0.944	0.786	0.792	0.815
Logistic Regression	0.897	0.950	0.944	0.955	0.933	0.978	0.947	0.932	0.962	0.975	0.866	0.929	0.897	0.955	0.923	0.871	0.837	0.891	0.893	0.871	0.831	0.947	0.967	0.938	0.955	0.840	0.964	0.786	0.833	0.871
Multi Layer Perceptron	0.877	0.950	0.946	0.955	0.933	0.978	0.920	0.955	0.981	0.921	0.900	0.929	0.897	0.955	0.923	0.871	0.837	0.891	0.893	0.899	0.913	0.921	0.967	0.938	0.955	0.840	0.964	0.786	0.878	0.871
Nearest Centroid	0.897	0.900	0.889	0.955	0.867	0.935	0.895	0.909	1.000	0.900	0.850	0.929	0.906	0.955	0.923	0.900	0.854	0.889	0.867	0.857	0.850	0.895	0.967	0.900	0.955	0.840	0.889	0.752	0.772	0.871
Nearest Neighbors	0.897	0.950	0.944	0.955	0.933	0.957	0.921	0.955	0.962	0.971	0.891	0.929	0.866	0.888	0.923	0.900	0.798	0.889	0.920	0.964	0.850	0.921	0.921	0.980	0.909	0.840	0.982	0.840	0.837	0.927
Passive Aggressive Classifier	0.877	0.799	0.944	0.955	0.809	0.907	0.781	0.898	0.962	1.000	0.765	0.912	0.731	0.955	0.941	0.900	0.878	0.891	0.893	0.885	0.850	0.865	1.000	0.897	0.755	0.840	0.891	0.786	0.818	0.871
Perceptron	0.917	0.950	0.944	0.955	0.933	0.957	0.920	0.821	0.885	0.971	0.882	0.929	0.842	0.888	0.816	0.871	0.875	0.909	0.893	0.835	0.963	0.947	0.967	0.938	0.955	0.840	0.982	0.786	0.813	0.871
Quadratic Discriminant Analysis	0.897	0.975	0.972	0.935	0.967	0.978	0.974	0.898	0.962	0.971	0.925	0.929	0.921	0.977	0.902	0.871	0.875	0.909	0.893	0.893	0.831	0.947	1.000	0.980	0.955	0.840	0.982	0.786	0.833	0.927
Random Forest	0.917	0.900	0.972	0.955	0.967	0.943	0.921	0.955	0.981	1.000	0.950	0.929	0.921	0.977	0.923	0.896	0.896	0.927	0.893	0.964	0.850	0.946	1.000	0.938	0.955	0.840	0.929	0.786	0.875	0.871
SVM - linear	0.897	0.950	0.944	0.955	0.967	0.957	0.947	0.932	0.962	0.975	0.900	0.929	0.938	0.955	0.923	0.871	0.875	0.927	0.893	0.871	0.850	0.921	0.967	0.980	0.909	0.840	0.944	0.786	0.833	0.871
SVM - poly	0.877	0.950	0.972	0.955	1.000	0.957	0.947	0.955	0.962	1.000	0.900	0.929	0.906	0.955	0.923	0.896	0.875	0.891	0.893	0.964	0.850	0.947	0.977	0.980	0.955	0.840	0.964	0.769	0.898	0.853
SVM - rbf	0.917	0.950	0.944	0.955	0.933	0.957	0.947	0.909	0.962	0.975	0.900	0.929	0.914	0.955	0.923	0.871	0.875	0.927	0.895	0.835	0.850	0.921	0.967	0.980	0.955	0.840	0.944	0.786	0.792	0.871

Table 4.38 | Balanced accuracy score for 30 L*u*v* image feature datasets for cell line U251

Classifiers	P1	P2	P3	P4	P5	P6	P7	P8	P9	P10	P11	P12	P13	P14	P15	P16	P17	P18	P19	P20	P21	P22	P23	P24	P25	P26	P27	P28	P29	P30
AdaBoost	0.897	0.925	0.944	0.909	0.933	0.943	0.920	0.888	1.000	0.971	0.891	0.929	0.921	0.888	0.923	0.871	0.819	0.927	0.893	0.921	0.831	0.895	0.967	0.938	0.955	0.895	0.964	0.840	0.795	0.871
Bernoulli Naive Bayes	0.897	0.900	0.944	0.955	0.967	0.913	0.895	0.909	0.955	0.900	0.900	0.929	0.906	0.955	0.923	0.846	0.854	0.927	0.865	0.893	0.850	0.895	0.967	0.900	0.955	0.912	0.909	0.786	0.813	0.871
Dummy Classifier	0.508	0.500	0.539	0.500	0.405	0.500	0.542	0.527	0.500	0.500	0.500	0.350	0.479	0.500	0.404	0.431	0.561	0.500	0.461	0.500	0.420	0.500	0.500	0.500	0.500	0.636	0.500	0.433	0.632	0.500
Extra Trees	0.875	0.875	0.917	0.955	0.867	0.978	0.895	0.932	0.955	0.832	0.853	0.929	0.866	0.955	0.885	0.871	0.875	0.722	0.737	0.893	0.850	0.947	0.967	0.980	0.864	0.769	0.833	0.500	0.708	0.871
Gaussian Naive Bayes	0.897	0.950	0.944	0.955	0.900	0.978	0.947	0.932	0.935	0.946	0.900	0.929	0.890	0.977	0.902	0.841	0.875	0.927	0.893	0.893	0.831	0.947	1.000	0.980	0.955	0.824	0.927	0.786	0.833	0.871
Gradient Boosting	0.897	0.925	0.944	0.935	0.933	0.943	0.920	0.888	0.981	0.975	0.862	0.929	0.874	0.955	0.902	0.837	0.857	0.909	0.893	0.943	0.813	0.892	1.000	0.980	0.955	0.895	0.929	0.769	0.815	0.853
**Helstrom Quantum Classifier**	0.897	0.875	0.895	0.955	0.867	0.899	0.921	0.944	0.909	0.925	0.925	0.929	0.882	0.877	0.923	0.871	0.854	0.889	0.921	0.893	0.831	0.893	1.000	0.980	0.955	0.895	0.946	0.824	0.917	0.909
Linear Discriminant Analysis	0.917	0.900	0.861	0.955	0.867	0.935	0.947	0.932	0.955	0.950	0.841	0.929	0.906	0.955	0.923	0.900	0.854	0.889	0.868	0.871	0.850	0.947	0.967	0.960	0.955	0.840	0.944	0.786	0.833	0.871
Logistic Regression	0.897	0.950	0.944	0.955	0.933	0.978	0.947	0.932	0.962	0.950	0.896	0.929	0.921	0.955	0.923	0.871	0.875	0.891	0.893	0.893	0.881	0.947	1.000	0.938	0.955	0.840	0.946	0.786	0.875	0.871
Multi Layer Perceptron	0.897	0.950	1.000	0.955	0.933	0.978	0.920	0.932	0.962	0.946	0.875	0.929	0.897	0.977	0.923	0.871	0.875	0.891	0.893	1.000	0.831	0.947	1.000	0.938	0.955	0.879	0.964	0.840	0.897	0.871
Nearest Centroid	0.875	0.875	0.889	0.955	0.833	0.935	0.868	0.909	0.955	0.900	0.825	0.929	0.882	0.955	0.923	0.871	0.833	0.889	0.893	0.893	0.850	0.895	0.967	0.960	0.955	0.840	0.944	0.786	0.813	0.871
Nearest Neighbors	0.917	0.900	0.944	1.000	0.900	0.943	0.921	0.832	0.962	0.950	0.891	0.929	0.890	0.933	0.920	0.871	0.819	0.889	0.868	0.978	0.850	0.867	1.000	0.938	0.955	0.824	0.909	0.840	0.833	0.927
Passive Aggressive Classifier	0.917	0.900	0.839	0.955	0.877	0.907	0.920	0.898	0.942	0.912	0.896	0.929	0.969	0.732	0.902	0.841	0.784	0.909	0.893	0.856	0.950	0.725	0.967	0.980	0.864	0.840	0.946	0.719	0.817	0.837
Perceptron	0.897	0.950	0.841	0.981	0.821	0.957	0.861	0.876	0.923	1.000	0.857	0.929	0.874	0.888	0.923	0.778	0.801	0.909	0.893	0.871	0.850	0.836	0.967	0.938	0.955	0.912	0.944	0.752	0.815	0.873
Quadratic Discriminant Analysis	0.897	0.975	0.972	0.955	0.967	0.943	0.974	0.932	0.942	0.971	0.896	0.929	0.921	0.977	0.902	0.871	0.875	0.909	0.893	0.907	0.850	0.947	1.000	0.980	0.955	0.840	0.982	0.786	0.833	0.927
Random Forest	0.917	0.925	0.944	0.955	0.967	0.943	0.920	0.921	0.981	0.971	0.862	0.929	0.897	0.977	0.923	0.866	0.875	0.927	0.893	0.893	0.831	0.946	1.000	0.980	0.955	0.895	0.929	0.786	0.875	0.871
SVM - linear	0.897	0.950	0.944	0.955	0.967	0.978	0.947	0.932	1.000	0.950	0.925	0.929	0.906	0.955	0.923	0.871	0.875	0.927	0.867	0.871	0.850	0.921	0.967	0.938	0.955	0.840	0.927	0.786	0.792	0.871
SVM - poly	0.897	0.975	0.972	1.000	0.867	0.957	0.947	0.932	1.000	0.975	0.866	0.929	0.890	0.921	0.864	0.871	0.798	0.909	0.893	0.978	0.831	0.895	1.000	0.980	0.955	0.840	0.964	0.786	0.775	0.853
SVM - rbf	0.897	0.950	0.944	0.955	0.967	0.978	0.947	0.932	1.000	0.975	0.900	0.929	0.914	0.955	0.923	0.871	0.875	0.927	0.867	0.893	0.850	0.921	0.967	0.938	0.955	0.840	0.982	0.786	0.792	0.871

Table 4.39 | Balanced accuracy score for 30 contrast image feature datasets for cell line U251

Classifiers	P1	P2	P3	P4	P5	P6	P7	P8	P9	P10	P11	P12	P13	P14	P15	P16	P17	P18	P19	P20	P21	P22	P23	P24	P25	P26	P27	P28	P29	P30
AdaBoost	1.000	0.971	0.974	0.981	0.933	0.978	1.000	0.921	0.962	1.000	0.975	1.000	1.000	0.967	1.000	0.975	0.979	0.927	0.946	0.978	0.950	0.972	0.977	0.958	1.000	0.983	0.889	0.950	0.938	0.982
Bernoulli Naive Bayes	0.958	0.946	0.974	1.000	0.900	0.826	0.974	0.886	0.981	0.950	0.900	1.000	0.906	0.886	0.962	0.900	0.920	0.909	0.946	0.929	0.950	0.947	0.944	0.735	1.000	0.983	0.964	0.967	0.897	0.982
Dummy Classifier	0.500	0.500	0.500	0.509	0.500	0.447	0.500	0.561	0.549	0.500	0.500	0.367	0.519	0.506	0.484	0.481	0.502	0.452	0.547	0.467	0.500	0.500	0.500	0.547	0.500	0.581	0.500	0.500	0.500	0.321
Extra Trees	0.708	0.850	0.861	0.500	0.867	0.500	1.000	0.967	0.981	0.882	0.824	0.714	0.738	0.921	0.846	0.803	0.808	0.500	0.500	0.929	0.500	0.974	0.744	0.500	0.500	0.500	0.500	0.500	0.500	0.500
Gaussian Naive Bayes	1.000	0.971	0.974	1.000	0.967	1.000	1.000	0.944	0.962	0.971	0.975	1.000	1.000	0.911	1.000	0.975	0.979	0.909	0.972	0.929	0.981	0.974	0.977	0.960	1.000	0.983	0.964	0.950	0.875	0.982
Gradient Boosting	1.000	0.971	1.000	0.981	0.933	0.978	0.972	0.921	0.916	1.000	0.975	1.000	0.976	0.944	1.000	0.916	0.941	0.927	0.920	0.978	0.950	0.890	0.977	0.958	1.000	0.912	0.889	0.967	0.958	0.982
**Helstrom Quantum Classifier**	0.958	0.971	1.000	1.000	0.967	0.978	1.000	0.921	0.981	1.000	0.925	1.000	0.938	0.955	1.000	0.975	0.958	0.927	0.972	0.964	0.950	0.974	0.977	0.980	1.000	0.983	0.889	0.983	0.875	0.982
Linear Discriminant Analysis	0.958	0.921	0.946	0.955	0.867	0.978	0.974	0.921	0.981	1.000	0.950	0.929	0.938	0.932	0.962	0.900	0.938	0.871	0.893	0.893	0.900	0.947	0.911	0.980	0.909	0.912	0.889	0.643	0.875	0.815
Logistic Regression	1.000	0.946	0.974	1.000	0.933	0.978	1.000	0.944	0.962	1.000	0.950	0.929	1.000	0.911	1.000	0.950	0.979	0.927	0.972	0.964	0.950	0.974	0.977	0.980	1.000	0.983	0.944	0.967	0.875	0.982
Multi Layer Perceptron	1.000	0.946	0.974	1.000	0.933	0.978	1.000	0.876	0.962	1.000	0.950	1.000	1.000	0.967	1.000	0.950	0.958	0.927	0.972	0.964	0.950	0.974	0.977	0.980	1.000	0.983	0.889	0.983	0.875	0.982
Nearest Centroid	0.958	0.946	0.974	0.955	0.867	0.935	0.947	0.944	0.962	0.921	0.950	0.929	0.882	0.888	0.878	0.871	0.875	0.927	0.972	0.964	0.900	0.920	0.944	0.900	0.955	0.983	0.889	0.912	0.938	0.927
Nearest Neighbors	1.000	0.971	1.000	1.000	0.967	0.978	1.000	0.898	0.890	1.000	1.000	1.000	0.976	0.955	0.962	0.975	0.958	0.927	1.000	0.964	0.950	0.946	0.977	0.938	1.000	0.983	1.000	0.983	0.897	0.982
Passive Aggressive Classifier	1.000	0.946	0.974	1.000	0.967	0.978	1.000	0.898	0.962	1.000	0.853	1.000	0.952	0.911	0.979	0.900	0.958	0.927	0.972	0.978	0.950	0.808	0.977	0.958	1.000	0.983	0.889	0.983	0.838	0.982
Perceptron	1.000	0.946	0.974	0.923	0.967	1.000	1.000	0.944	0.981	1.000	0.916	1.000	1.000	0.888	0.923	0.850	0.958	0.927	0.946	0.929	0.950	0.694	0.977	0.938	1.000	0.983	0.944	0.917	0.897	0.982
Quadratic Discriminant Analysis	1.000	0.971	0.974	1.000	0.967	1.000	1.000	0.944	0.962	0.971	0.975	1.000	1.000	0.911	1.000	0.975	0.979	0.927	0.972	0.964	0.981	0.974	0.977	0.980	1.000	0.983	0.964	0.967	0.875	0.982
Random Forest	1.000	0.971	1.000	1.000	1.000	0.978	1.000	0.921	0.962	1.000	0.975	1.000	1.000	0.967	1.000	0.975	0.979	0.871	0.972	1.000	0.950	0.947	0.977	0.958	1.000	1.000	0.889	0.967	0.938	0.982
SVM - linear	1.000	0.971	0.974	1.000	0.967	0.978	1.000	0.944	0.962	1.000	0.950	1.000	1.000	0.911	1.000	0.975	0.958	0.927	0.972	0.964	0.931	0.974	0.977	0.980	1.000	0.983	0.944	0.967	0.897	0.982
SVM - poly	1.000	0.971	1.000	1.000	0.967	1.000	1.000	0.921	0.962	1.000	0.975	1.000	1.000	0.944	1.000	0.975	0.979	0.927	0.972	0.964	0.950	0.947	0.977	0.980	1.000	0.983	0.944	0.950	0.897	0.982
SVM - rbf	1.000	0.971	0.974	1.000	0.967	0.978	1.000	0.921	0.962	1.000	0.950	1.000	1.000	0.911	1.000	0.946	0.958	0.927	0.972	0.964	0.950	0.974	0.977	0.980	1.000	0.983	1.000	0.967	0.855	0.982

Table 4.40 | Balanced accuracy score for 30 correlation image feature datasets for cell line U251

Classifiers	P1	P2	P3	P4	P5	P6	P7	P8	P9	P10	P11	P12	P13	P14	P15	P16	P17	P18	P19	P20	P21	P22	P23	P24	P25	P26	P27	P28	P29	P30
AdaBoost	0.608	0.812	0.759	0.715	0.830	0.764	0.811	0.742	0.858	0.812	0.803	0.736	0.778	0.820	0.718	0.703	0.840	0.817	0.759	0.848	0.781	0.700	0.553	0.633	0.780	0.857	0.927	0.681	0.813	0.871
Bernoulli Naive Bayes	0.528	0.657	0.754	0.500	0.562	0.500	0.838	0.608	0.858	0.565	0.665	0.840	0.676	0.797	0.665	0.574	0.763	0.556	0.548	0.592	0.681	0.620	0.655	0.672	0.689	0.736	0.500	0.450	0.753	0.669
Dummy Classifier	0.500	0.494	0.500	0.500	0.618	0.500	0.355	0.500	0.500	0.447	0.500	0.405	0.424	0.500	0.561	0.500	0.428	0.377	0.572	0.500	0.500	0.500	0.483	0.490	0.444	0.500	0.431	0.526	0.425	0.577
Extra Trees	0.500	0.500	0.779	0.500	0.577	0.500	0.754	0.744	0.871	0.647	0.529	0.500	0.500	0.752	0.500	0.665	0.538	0.500	0.500	0.536	0.500	0.727	0.500	0.500	0.500	0.500	0.500	0.500	0.500	0.500
Gaussian Naive Bayes	0.523	0.607	0.731	0.526	0.742	0.693	0.811	0.686	0.813	0.703	0.732	0.752	0.723	0.718	0.739	0.703	0.739	0.502	0.577	0.635	0.813	0.730	0.755	0.732	0.591	0.769	0.760	0.643	0.730	0.760
Gradient Boosting	0.733	0.896	0.917	0.753	0.842	0.921	0.754	0.877	0.904	0.891	0.803	0.912	0.833	0.888	0.857	0.791	0.819	0.909	0.816	0.762	0.813	0.835	0.730	0.938	0.864	0.857	0.927	0.769	0.793	0.944
**Helstrom Quantum Classifier**	0.673	0.687	0.864	0.677	0.698	0.778	0.863	0.732	0.748	0.812	0.871	0.840	0.810	0.798	0.837	0.737	0.739	0.871	0.918	0.806	0.763	0.782	0.855	0.878	0.671	0.840	0.891	0.745	0.772	0.817
Linear Discriminant Analysis	0.545	0.657	0.731	0.526	0.620	0.585	0.728	0.653	0.767	0.649	0.694	0.786	0.771	0.741	0.700	0.728	0.662	0.484	0.550	0.542	0.794	0.649	0.709	0.648	0.617	0.840	0.722	0.643	0.710	0.760
Logistic Regression	0.545	0.657	0.731	0.545	0.620	0.585	0.728	0.653	0.767	0.649	0.694	0.786	0.795	0.741	0.721	0.753	0.662	0.484	0.550	0.542	0.794	0.649	0.709	0.648	0.617	0.840	0.722	0.643	0.710	0.760
Multi Layer Perceptron	0.690	0.678	0.946	0.981	0.967	0.885	0.864	0.753	0.767	0.821	0.853	0.912	0.787	0.865	0.979	0.841	0.801	0.982	0.920	0.806	0.744	0.727	0.855	0.918	0.864	0.824	0.780	0.719	0.752	0.817
Nearest Centroid	0.553	0.724	0.731	0.645	0.664	0.626	0.728	0.664	0.839	0.599	0.762	0.757	0.732	0.786	0.739	0.653	0.739	0.524	0.623	0.477	0.802	0.727	0.697	0.552	0.767	0.845	0.655	0.669	0.815	0.726
Nearest Neighbors	0.732	0.707	0.892	0.806	0.798	0.849	0.864	0.788	0.851	0.921	0.857	1.000	0.874	0.832	0.822	0.841	0.795	0.946	0.813	0.863	0.863	0.754	0.865	0.897	0.773	0.840	0.964	0.593	0.795	0.873
Passive Aggressive Classifier	0.470	0.410	0.320	0.528	0.483	0.641	0.643	0.509	0.664	0.581	0.549	0.779	0.676	0.686	0.756	0.782	0.413	0.562	0.500	0.477	0.600	0.542	0.548	0.500	0.500	0.667	0.500	0.664	0.595	0.710
Perceptron	0.490	0.381	0.626	0.469	0.609	0.620	0.813	0.568	0.724	0.732	0.543	0.707	0.549	0.291	0.739	0.678	0.593	0.526	0.570	0.669	0.683	0.623	0.559	0.500	0.559	0.733	0.484	0.450	0.425	0.671
Quadratic Discriminant Analysis	0.483	0.599	0.782	0.526	0.709	0.728	0.784	0.698	0.767	0.753	0.732	0.857	0.795	0.774	0.760	0.703	0.756	0.593	0.604	0.679	0.826	0.703	0.755	0.752	0.636	0.752	0.742	0.610	0.730	0.760
Random Forest	0.627	0.791	0.861	0.636	0.888	0.849	0.781	0.798	0.787	0.837	0.862	0.857	0.857	0.888	0.938	0.812	0.819	0.871	0.630	0.877	0.863	0.728	0.832	0.835	0.799	0.857	1.000	0.643	0.813	0.815
SVM - linear	0.545	0.657	0.731	0.500	0.620	0.500	0.784	0.653	0.767	0.649	0.703	0.500	0.740	0.741	0.739	0.753	0.623	0.484	0.550	0.542	0.794	0.648	0.721	0.708	0.617	0.840	0.500	0.571	0.730	0.760
SVM - poly	0.690	0.682	0.917	0.696	0.865	0.863	0.728	0.877	0.813	0.841	0.857	0.879	0.778	0.753	0.857	0.891	0.724	0.909	0.788	0.842	0.863	0.810	0.855	0.938	0.722	0.824	0.927	0.774	0.857	0.798
SVM - rbf	0.545	0.632	0.731	0.500	0.653	0.500	0.811	0.676	0.813	0.649	0.699	0.752	0.716	0.786	0.780	0.782	0.623	0.484	0.577	0.585	0.763	0.673	0.732	0.667	0.572	0.752	0.500	0.571	0.730	0.760

Table 4.41 | Balanced accuracy score for 30 energy image feature datasets for cell line U251

Classifiers	P1	P2	P3	P4	P5	P6	P7	P8	P9	P10	P11	P12	P13	P14	P15
AdaBoost	1.000	1.000	1.000	0.981	0.967	1.000	0.974	1.000	1.000	1.000	0.946	1.000	1.000	0.977	0.962
Bernoulli Naive Bayes	0.940	0.912	0.838	0.827	0.955	0.964	0.972	0.933	0.885	0.971	0.941	0.807	1.000	0.967	0.920
Dummy Classifier	0.388	0.524	0.458	0.500	0.494	0.500	0.484	0.500	0.500	0.500	0.500	0.500	0.500	0.548	0.500
Extra Trees	0.583	0.500	0.889	0.500	0.967	0.500	0.946	0.967	0.981	0.941	0.853	0.500	0.929	0.977	0.923
Gaussian Naive Bayes	0.917	1.000	0.947	1.000	1.000	1.000	0.944	1.000	1.000	1.000	0.941	1.000	1.000	0.977	0.962
Gradient Boosting	1.000	1.000	1.000	1.000	0.933	1.000	0.974	1.000	1.000	1.000	0.975	1.000	0.921	0.977	0.941
**Helstrom Quantum Classifier**	1.000	1.000	1.000	1.000	1.000	1.000	0.974	0.967	0.981	0.971	0.971	1.000	1.000	1.000	0.920
Linear Discriminant Analysis	0.960	1.000	0.921	1.000	1.000	0.929	0.972	0.933	0.923	0.971	0.941	0.983	0.976	0.967	0.917
Logistic Regression	1.000	1.000	0.974	1.000	1.000	0.929	1.000	0.967	0.962	0.971	0.941	1.000	1.000	0.977	0.941
Multi Layer Perceptron	1.000	1.000	1.000	1.000	1.000	1.000	1.000	0.967	0.962	1.000	0.941	1.000	1.000	0.977	0.941
Nearest Centroid	0.960	0.941	0.865	0.923	0.955	1.000	0.917	0.933	0.942	0.916	0.941	0.950	0.952	0.933	0.938
Nearest Neighbors	1.000	1.000	1.000	1.000	1.000	0.964	0.972	0.967	0.981	0.971	0.946	1.000	1.000	1.000	0.920
Passive Aggressive Classifier	1.000	1.000	0.947	0.981	1.000	1.000	0.972	0.967	1.000	0.900	0.912	1.000	1.000	0.977	0.941
Perceptron	1.000	1.000	0.947	0.981	0.967	0.964	0.918	0.967	0.909	1.000	0.941	0.929	1.000	1.000	0.885
Quadratic Discriminant Analysis	0.917	1.000	1.000	1.000	1.000	1.000	1.000	1.000	0.981	1.000	0.941	1.000	1.000	0.977	0.962
Random Forest	1.000	1.000	1.000	1.000	1.000	1.000	0.974	1.000	1.000	1.000	0.975	1.000	1.000	0.977	0.962
SVM - linear	1.000	1.000	1.000	1.000	1.000	0.964	1.000	0.967	0.981	1.000	0.971	1.000	1.000	1.000	0.962
SVM - poly	1.000	1.000	1.000	1.000	1.000	0.964	0.974	1.000	1.000	1.000	0.946	1.000	1.000	0.977	0.920
SVM - rbf	1.000	1.000	1.000	1.000	1.000	1.000	0.974	0.967	0.981	1.000	0.971	1.000	1.000	1.000	0.962

Classifiers	P16	P17	P18	P19	P20	P21	P22	P23	P24	P25	P26	P27	P28	P29	P30
AdaBoost	0.925	0.938	0.927	0.946	1.000	0.931	0.947	1.000	1.000	1.000	0.983	1.000	0.824	0.835	0.982
Bernoulli Naive Bayes	0.887	0.958	0.615	0.732	0.848	0.652	0.917	0.886	0.980	0.729	0.879	0.782	0.505	0.817	0.742
Dummy Classifier	0.500	0.500	0.377	0.500	0.604	0.500	0.500	0.362	0.612	0.500	0.455	0.522	0.500	0.527	0.486
Extra Trees	0.828	0.671	0.500	0.500	0.786	0.500	0.974	0.533	0.500	0.500	0.500	0.500	0.500	0.500	0.500
Gaussian Naive Bayes	0.950	0.938	0.927	0.946	0.978	0.931	0.972	1.000	0.980	1.000	0.912	0.964	0.895	0.918	0.927
Gradient Boosting	0.950	0.938	0.927	0.920	1.000	0.931	0.920	1.000	0.980	0.981	0.983	0.964	0.824	0.938	0.982
**Helstrom Quantum Classifier**	0.946	0.958	0.944	0.974	0.978	0.963	0.947	1.000	0.938	0.962	0.950	0.964	0.929	0.960	0.982
Linear Discriminant Analysis	0.887	0.920	0.927	0.762	0.891	0.844	0.944	0.909	0.958	1.000	0.967	0.964	0.769	0.920	0.982
Logistic Regression	0.900	0.958	0.927	0.946	0.978	0.931	0.918	1.000	0.958	0.981	0.983	0.964	0.895	0.940	0.982
Multi Layer Perceptron	0.946	0.958	0.927	0.946	0.978	0.931	0.893	1.000	0.958	0.981	0.983	0.964	0.879	0.918	0.982
Nearest Centroid	0.782	0.938	0.927	0.918	0.913	0.839	0.944	0.909	0.938	0.813	0.967	0.929	0.850	0.980	0.982
Nearest Neighbors	0.975	0.920	0.944	0.974	1.000	0.963	1.000	1.000	0.958	1.000	0.983	0.964	0.824	1.000	0.982
Passive Aggressive Classifier	0.946	0.766	0.927	0.946	0.978	0.931	0.974	0.932	0.958	1.000	0.983	0.964	0.900	0.960	0.982
Perceptron	0.875	0.899	0.927	0.946	1.000	0.931	0.918	0.977	0.958	0.981	0.983	0.946	0.917	0.877	0.982
Quadratic Discriminant Analysis	0.950	0.958	0.927	0.974	0.978	0.931	1.000	1.000	0.980	0.935	0.983	0.964	0.895	0.898	0.927
Random Forest	0.925	0.938	0.871	0.946	1.000	0.931	0.893	1.000	1.000	0.981	0.983	1.000	0.824	0.938	0.982
SVM - linear	0.946	0.958	0.927	0.946	1.000	0.931	0.974	1.000	0.958	1.000	0.983	0.964	0.895	0.940	0.982
SVM - poly	0.946	0.938	0.927	0.946	1.000	0.931	0.921	1.000	0.958	0.981	0.983	0.982	0.912	0.918	0.982
SVM - rbf	0.946	0.958	0.927	0.946	1.000	0.931	0.974	1.000	0.980	1.000	0.983	0.964	0.895	0.940	0.982

Table 4.42 | Balanced accuracy score for 30 homogeneity image feature datasets for cell line U251

Classifiers	P1	P2	P3	P4	P5	P6	P7	P8	P9	P10	P11	P12	P13	P14	P15
AdaBoost	1.000	1.000	1.000	1.000	0.967	1.000	0.974	1.000	1.000	1.000	0.975	1.000	0.952	0.955	0.962
Bernoulli Naive Bayes	1.000	1.000	1.000	1.000	1.000	0.978	0.974	1.000	1.000	1.000	0.975	0.983	1.000	1.000	0.962
Dummy Classifier	0.500	0.500	0.500	0.500	0.500	0.500	0.500	0.500	0.329	0.510	0.500	0.707	0.500	0.500	0.659
Extra Trees	0.958	1.000	1.000	0.818	0.967	0.679	0.974	0.933	1.000	1.000	0.971	0.857	0.952	0.977	0.962
Gaussian Naive Bayes	1.000	1.000	1.000	1.000	0.967	0.978	0.974	1.000	1.000	1.000	0.946	1.000	1.000	0.977	0.962
Gradient Boosting	1.000	1.000	1.000	1.000	0.967	1.000	0.974	1.000	0.981	1.000	0.946	1.000	0.976	1.000	0.941
**Helstrom Quantum Classifier**	1.000	1.000	1.000	1.000	1.000	1.000	0.974	1.000	1.000	1.000	0.975	1.000	1.000	1.000	0.962
Linear Discriminant Analysis	1.000	1.000	1.000	1.000	0.967	1.000	0.974	1.000	1.000	1.000	0.975	1.000	1.000	1.000	0.962
Logistic Regression	1.000	1.000	0.974	1.000	0.967	1.000	0.974	1.000	0.981	1.000	1.000	1.000	1.000	1.000	0.962
Multi Layer Perceptron	1.000	1.000	0.947	1.000	1.000	1.000	0.974	0.967	0.981	1.000	0.971	1.000	1.000	0.977	0.962
Nearest Centroid	1.000	1.000	0.974	1.000	1.000	1.000	0.974	0.967	1.000	0.946	0.975	1.000	0.976	1.000	0.938
Nearest Neighbors	1.000	1.000	1.000	1.000	0.967	0.978	0.974	1.000	1.000	1.000	0.975	1.000	1.000	1.000	0.962
Passive Aggressive Classifier	1.000	1.000	0.814	1.000	0.933	0.978	0.972	0.967	1.000	1.000	0.975	1.000	0.976	0.955	0.941
Perceptron	1.000	1.000	0.974	1.000	0.967	0.978	1.000	1.000	0.962	1.000	0.971	1.000	0.929	0.977	0.864
Quadratic Discriminant Analysis	1.000	1.000	1.000	1.000	0.967	0.978	1.000	1.000	1.000	1.000	0.946	1.000	1.000	0.977	0.962
Random Forest	1.000	1.000	1.000	1.000	0.967	1.000	0.974	1.000	1.000	1.000	0.971	1.000	1.000	0.977	0.962
SVM - linear	1.000	1.000	1.000	1.000	0.967	1.000	0.974	1.000	1.000	1.000	1.000	1.000	1.000	0.977	0.962
SVM - poly	1.000	1.000	0.972	1.000	0.977	1.000	0.946	1.000	1.000	1.000	1.000	1.000	1.000	0.977	0.962
SVM - rbf	1.000	1.000	1.000	1.000	1.000	1.000	0.974	1.000	1.000	1.000	1.000	1.000	1.000	1.000	0.962

Classifiers	P16	P17	P18	P19	P20	P21	P22	P23	P24	P25	P26	P27	P28	P29	P30
AdaBoost	0.950	0.958	0.927	0.946	0.978	0.931	0.974	1.000	0.980	1.000	0.983	0.982	0.862	0.917	0.982
Bernoulli Naive Bayes	0.946	0.917	0.909	0.972	1.000	0.926	0.974	1.000	0.980	0.962	0.983	0.946	0.681	0.960	0.982
Dummy Classifier	0.565	0.623	0.542	0.462	0.396	0.415	0.500	0.500	0.500	0.500	0.564	0.500	0.471	0.527	0.490
Extra Trees	0.946	0.938	0.889	0.500	1.000	0.750	0.974	0.967	0.500	0.818	0.500	1.000	0.500	0.792	0.889
Gaussian Naive Bayes	0.950	0.958	0.927	0.946	1.000	0.950	0.974	1.000	0.980	1.000	0.983	0.982	0.895	0.938	0.982
Gradient Boosting	0.950	0.920	0.927	0.946	0.978	0.931	0.892	1.000	1.000	1.000	0.983	0.964	0.862	0.980	0.982
**Helstrom Quantum Classifier**	0.975	0.896	0.927	0.946	1.000	0.950	0.974	1.000	0.980	1.000	0.983	0.982	0.895	0.980	0.982
Linear Discriminant Analysis	0.946	0.917	0.927	0.972	1.000	0.931	0.974	1.000	1.000	1.000	0.983	0.982	0.840	0.918	0.982
Logistic Regression	0.975	0.938	0.927	0.946	0.978	0.950	0.974	1.000	1.000	1.000	0.983	0.982	0.862	0.918	0.982
Multi Layer Perceptron	0.921	0.878	0.927	0.946	0.978	0.931	0.974	1.000	1.000	1.000	0.983	0.982	0.895	0.897	0.982
Nearest Centroid	0.871	0.917	0.927	0.946	1.000	0.913	0.946	0.977	1.000	1.000	0.983	0.964	0.862	0.980	0.982
Nearest Neighbors	0.950	0.899	0.927	0.972	1.000	0.931	0.974	1.000	0.980	1.000	0.983	0.982	0.912	0.877	0.982
Passive Aggressive Classifier	0.950	0.864	0.927	0.972	1.000	0.931	0.946	1.000	1.000	1.000	0.983	0.929	0.879	0.898	0.982
Perceptron	0.900	0.804	0.927	0.946	0.893	0.931	0.868	1.000	1.000	1.000	0.983	0.964	0.807	0.940	0.982
Quadratic Discriminant Analysis	0.950	0.958	0.927	0.946	1.000	0.950	0.974	1.000	1.000	1.000	0.983	0.982	0.895	0.918	0.982
Random Forest	0.900	0.958	0.927	0.946	0.978	0.950	0.947	1.000	1.000	1.000	1.000	0.982	0.862	0.938	0.982
SVM - linear	0.946	0.958	0.927	0.972	1.000	0.950	0.974	0.977	1.000	1.000	0.983	0.982	0.840	0.897	0.982
SVM - poly	0.950	0.938	0.927	0.974	1.000	0.950	1.000	1.000	0.958	1.000	0.983	0.982	0.912	0.958	0.982
SVM - rbf	0.975	0.958	0.927	0.946	1.000	0.950	0.974	0.977	1.000	1.000	0.983	0.982	0.840	0.958	0.982

Table 4.43 | AUROC score for 30 RGB image feature datasets for cell line U251

Classifiers	P1	P2	P3	P4	P5	P6	P7	P8	P9	P10	P11	P12	P13	P14	P15
AdaBoost	0.917	0.950	0.944	0.955	0.933	0.943	0.921	0.955	0.981	1.000	0.900	0.929	0.921	0.832	0.923
Bernoulli Naive Bayes	0.917	0.875	0.944	0.955	0.933	0.913	0.895	0.886	0.955	0.866	0.825	0.929	0.906	0.932	0.923
Dummy Classifier	0.500	0.440	0.500	0.500	0.500	0.635	0.377	0.494	0.500	0.599	0.500	0.471	0.500	0.482	0.540
Extra Trees	0.917	0.875	0.889	0.955	0.900	0.957	0.893	0.876	0.909	0.857	0.950	0.929	0.762	0.921	0.885
Gaussian Naive Bayes	0.897	0.925	0.944	0.935	0.967	0.957	0.895	0.955	0.935	1.000	0.900	0.929	0.945	0.921	0.923
Gradient Boosting	0.917	0.950	1.000	0.955	0.977	0.978	0.947	0.921	0.981	1.000	0.887	0.929	0.897	0.944	0.902
**Helstrom Quantum Classifier**	0.917	0.950	0.944	0.955	0.833	0.935	0.895	0.909	1.000	0.950	0.925	0.929	0.906	0.955	0.923
Linear Discriminant Analysis	0.917	0.875	0.861	0.955	0.867	0.935	0.947	0.932	0.981	0.925	0.841	0.929	0.906	0.955	0.923
Logistic Regression	0.917	0.950	0.944	0.955	0.933	0.978	0.947	0.932	0.962	0.975	0.866	0.929	0.897	0.955	0.923
Multi Layer Perceptron	0.917	0.950	0.972	0.955	0.933	0.978	0.920	0.955	1.000	1.000	0.946	0.929	0.897	0.921	0.923
Nearest Neighbors	0.917	0.925	0.944	0.955	0.967	0.943	0.921	0.932	1.000	0.975	0.900	0.929	0.882	0.955	0.923
Quadratic Discriminant Analysis	0.897	0.975	0.972	0.935	0.967	0.978	0.974	0.898	0.962	0.971	0.925	0.929	0.921	0.977	0.902
Random Forest	0.917	0.925	0.972	0.955	0.944	0.943	0.947	0.955	0.962	1.000	0.925	0.929	0.921	0.977	0.923
SVM - linear	0.917	0.950	0.944	0.955	0.967	0.957	0.921	0.932	1.000	0.975	0.896	0.929	0.945	0.955	0.923
SVM - poly	0.917	0.921	0.889	0.909	0.900	0.943	0.921	0.955	0.727	1.000	0.871	0.929	0.969	0.855	0.923
SVM - rbf	0.917	0.950	0.583	0.955	0.933	0.978	0.947	0.909	1.000	0.975	0.896	0.929	0.945	0.955	0.923

Classifiers	P16	P17	P18	P19	P20	P21	P22	P23	P24	P25	P26	P27	P28	P29	P30
AdaBoost	0.896	0.917	0.927	0.893	0.964	0.900	0.893	1.000	0.938	0.916	0.912	0.964	0.786	0.833	0.871
Bernoulli Naive Bayes	0.846	0.854	0.927	0.867	0.857	0.850	0.868	0.921	0.858	0.955	0.840	0.946	0.757	0.833	0.871
Dummy Classifier	0.500	0.500	0.500	0.500	0.500	0.500	0.500	0.494	0.590	0.500	0.367	0.500	0.500	0.500	0.500
Extra Trees	0.871	0.822	0.833	0.500	0.857	0.700	0.895	0.967	0.833	0.500	0.714	0.500	0.500	0.500	0.778
Gaussian Naive Bayes	0.871	0.896	0.927	0.841	0.857	0.850	0.947	0.921	0.938	0.955	0.824	0.944	0.769	0.792	0.871
Gradient Boosting	0.896	0.896	0.927	0.893	0.964	0.831	0.946	0.977	0.938	0.955	0.912	0.929	0.769	0.835	0.853
**Helstrom Quantum Classifier**	0.900	0.819	0.889	0.789	0.929	0.850	0.842	0.967	0.920	0.909	0.840	0.944	0.752	0.813	0.871
Linear Discriminant Analysis	0.900	0.816	0.889	0.842	0.800	0.800	0.947	0.967	0.960	0.955	0.786	0.944	0.786	0.792	0.815
Logistic Regression	0.871	0.837	0.891	0.893	0.871	0.831	0.921	0.967	0.938	0.955	0.840	0.964	0.786	0.833	0.871
Multi Layer Perceptron	0.871	0.854	0.891	0.867	0.943	0.931	0.921	0.932	0.938	0.897	0.840	0.964	0.786	0.878	0.909
Nearest Neighbors	0.900	0.798	0.889	0.921	0.929	0.850	0.921	1.000	0.980	0.909	0.840	0.889	0.786	0.875	0.871
Quadratic Discriminant Analysis	0.871	0.875	0.909	0.893	0.893	0.831	0.947	1.000	0.980	0.955	0.840	0.982	0.786	0.833	0.927
Random Forest	0.896	0.896	0.927	0.893	0.929	0.831	0.946	1.000	0.938	0.955	0.840	0.929	0.786	0.813	0.871
SVM - linear	0.871	0.854	0.927	0.895	0.871	0.850	0.921	0.967	0.980	0.909	0.840	0.944	0.786	0.833	0.871
SVM - poly	0.871	0.854	0.889	0.842	0.964	0.850	0.947	0.967	0.980	0.890	0.840	0.964	0.769	0.792	0.871
SVM - rbf	0.871	0.500	0.927	0.842	0.536	0.850	0.921	0.600	0.980	0.500	0.714	0.944	0.786	0.833	0.871

Results exclude Nearest Centroid, Passive Aggressive Classifier and Perceptron.

Table 4.44 | AUROC score for 30 L*u*v* image feature datasets for cell line U251

Classifiers	P1	P2	P3	P4	P5	P6	P7	P8	P9	P10	P11	P12	P13	P14	P15
AdaBoost	0.917	0.950	0.972	0.909	0.933	0.943	0.920	0.888	0.981	0.971	0.891	0.929	0.921	0.888	0.923
Bernoulli Naive Bayes	0.897	0.900	0.944	0.955	0.967	0.913	0.895	0.909	0.955	0.900	0.900	0.929	0.906	0.955	0.923
Dummy Classifier	0.500	0.500	0.500	0.500	0.483	0.491	0.459	0.500	0.500	0.606	0.500	0.500	0.500	0.448	0.500
Extra Trees	0.875	0.875	0.861	0.818	0.833	0.978	0.895	0.921	0.955	0.916	0.900	0.929	0.747	0.955	0.885
Gaussian Naive Bayes	0.897	0.950	0.944	0.955	0.900	0.978	0.947	0.932	0.935	0.946	0.900	0.929	0.890	0.977	0.902
Gradient Boosting	0.877	0.925	1.000	0.935	0.967	0.943	0.920	0.888	0.962	0.971	0.891	0.929	0.897	0.911	0.902
**Helstrom Quantum Classifier**	0.917	0.900	0.972	0.955	0.867	0.935	0.921	0.932	0.955	0.950	0.875	0.929	0.906	0.977	0.923
Linear Discriminant Analysis	0.917	0.900	0.861	0.955	0.867	0.935	0.947	0.932	0.955	0.950	0.841	0.929	0.906	0.955	0.923
Logistic Regression	0.897	0.950	0.944	0.955	0.967	0.978	0.947	0.921	0.981	0.950	0.896	0.929	0.874	0.955	0.923
Multi Layer Perceptron	0.897	0.950	1.000	0.955	0.967	0.921	0.947	0.932	0.981	0.971	0.900	0.929	0.850	0.977	0.923
Nearest Neighbors	0.917	0.900	0.944	0.955	0.900	0.943	0.921	0.898	1.000	0.950	0.925	0.929	0.906	0.888	0.920
Quadratic Discriminant Analysis	0.897	0.975	0.972	0.955	0.967	0.943	0.974	0.932	0.942	0.971	0.896	0.929	0.921	0.977	0.902
Random Forest	0.917	0.925	1.000	0.955	1.000	0.943	0.920	0.921	0.981	1.000	0.891	0.929	0.945	0.944	0.923
SVM - linear	0.897	0.950	0.944	0.955	0.900	0.957	0.947	0.932	0.955	0.950	0.925	0.929	0.890	0.955	0.923
SVM - poly	0.917	0.875	0.917	0.955	0.867	0.943	0.947	0.932	0.955	0.950	0.900	0.929	0.890	0.955	0.902
SVM - rbf	0.897	0.900	0.667	0.955	0.900	0.978	0.947	0.932	0.955	0.925	0.825	0.929	0.945	0.955	0.923

Classifiers	P16	P17	P18	P19	P20	P21	P22	P23	P24	P25	P26	P27	P28	P29	P30
AdaBoost	0.841	0.896	0.927	0.893	0.943	0.831	0.921	0.967	0.938	0.916	0.895	0.964	0.769	0.815	0.871
Bernoulli Naive Bayes	0.846	0.854	0.927	0.865	0.893	0.850	0.895	0.967	0.900	0.955	0.912	0.909	0.786	0.813	0.871
Dummy Classifier	0.500	0.421	0.500	0.500	0.467	0.409	0.433	0.406	0.550	0.514	0.636	0.413	0.531	0.500	0.484
Extra Trees	0.871	0.896	0.833	0.737	0.857	0.850	0.895	0.967	0.500	0.773	0.769	0.833	0.500	0.792	0.871
Gaussian Naive Bayes	0.841	0.875	0.927	0.893	0.893	0.831	0.947	1.000	0.980	0.955	0.824	0.927	0.786	0.833	0.871
Gradient Boosting	0.866	0.857	0.909	0.892	0.921	0.831	0.892	0.977	0.897	0.916	0.895	0.929	0.786	0.835	0.853
**Helstrom Quantum Classifier**	0.871	0.816	0.889	0.895	0.893	0.850	0.921	1.000	0.980	0.909	0.769	0.964	0.824	0.833	0.871
Linear Discriminant Analysis	0.900	0.854	0.889	0.868	0.871	0.850	0.947	0.967	0.960	0.955	0.840	0.944	0.786	0.833	0.871
Logistic Regression	0.871	0.896	0.891	0.867	0.885	0.881	0.947	0.967	0.938	0.955	0.840	0.982	0.786	0.875	0.871
Multi Layer Perceptron	0.871	0.875	0.891	0.867	1.000	0.831	0.947	0.932	0.938	0.955	0.895	0.927	0.786	0.857	0.871
Nearest Neighbors	0.871	0.816	0.889	0.868	0.893	0.850	0.867	1.000	0.938	0.955	0.840	1.000	0.786	0.875	0.871
Quadratic Discriminant Analysis	0.871	0.875	0.909	0.893	0.907	0.850	0.947	1.000	0.980	0.955	0.840	0.982	0.786	0.833	0.927
Random Forest	0.896	0.896	0.927	0.893	0.929	0.831	0.920	1.000	0.980	0.955	0.895	0.929	0.786	0.855	0.871
SVM - linear	0.871	0.875	0.889	0.895	0.871	0.850	0.921	0.967	0.938	0.955	0.840	0.944	0.786	0.792	0.871
SVM - poly	0.871	0.798	0.889	0.842	0.978	0.850	0.895	1.000	0.980	0.955	0.714	0.927	0.786	0.833	0.871
SVM - rbf	0.871	0.875	0.871	0.500	0.893	0.850	0.895	0.567	0.980	0.500	0.500	0.927	0.786	0.833	0.500

Results exclude Nearest Centroid, Passive Aggressive Classifier and Perceptron.

Table 4.45 | AUROC score for 30 contrast image feature datasets for cell line U251

Classifiers	P1	P2	P3	P4	P5	P6	P7	P8	P9	P10	P11	P12	P13	P14	P15	P16	P17	P18	P19	P20	P21	P22	P23	P24	P25	P26	P27	P28	P29	P30
AdaBoost	1.000	0.971	1.000	0.981	0.933	0.978	1.000	0.967	0.962	1.000	0.975	1.000	1.000	0.967	1.000	0.975	0.979	0.927	0.972	0.957	0.950	0.918	0.977	0.958	1.000	0.983	0.889	0.983	0.938	0.982
Bernoulli Naive Bayes	0.958	0.946	0.974	1.000	0.900	0.826	0.974	0.886	0.981	0.950	0.900	1.000	0.906	0.886	0.962	0.900	0.920	0.909	0.946	0.929	0.950	0.947	0.944	0.735	1.000	0.983	0.964	0.967	0.897	0.982
Dummy Classifier	0.385	0.500	0.500	0.612	0.552	0.500	0.405	0.492	0.406	0.500	0.347	0.500	0.500	0.517	0.588	0.500	0.500	0.653	0.500	0.500	0.500	0.513	0.500	0.548	0.500	0.460	0.500	0.500	0.500	0.500
Extra Trees	0.708	0.825	0.806	0.500	0.667	0.500	1.000	0.944	0.962	0.882	0.500	0.500	0.500	0.932	0.692	0.828	0.538	0.500	0.500	0.821	0.500	0.808	0.677	0.500	0.500	0.500	0.500	0.500	0.500	0.500
Gaussian Naive Bayes	1.000	0.971	0.974	1.000	0.967	1.000	1.000	0.944	0.962	0.971	0.975	1.000	1.000	0.911	1.000	0.975	0.979	0.909	0.972	0.929	0.981	0.974	0.977	0.960	1.000	0.983	0.964	0.950	0.875	0.982
Gradient Boosting	1.000	0.971	1.000	0.981	0.944	0.978	1.000	0.898	0.935	1.000	0.975	1.000	0.976	0.944	1.000	0.925	1.000	0.927	0.920	0.957	0.950	0.946	0.977	0.958	1.000	0.983	0.889	0.967	0.938	0.982
**Helstrom Quantum Classifier**	1.000	0.971	1.000	0.955	0.933	0.978	1.000	0.898	0.981	1.000	0.925	1.000	0.938	0.955	1.000	0.950	0.938	0.927	0.737	0.929	0.750	0.947	0.977	0.980	1.000	0.983	0.944	0.983	0.875	0.982
Linear Discriminant Analysis	0.958	0.921	0.946	0.955	0.867	0.978	0.974	0.921	0.981	1.000	0.950	0.929	0.938	0.932	0.962	0.900	0.938	0.871	0.893	0.893	0.900	0.947	0.911	0.980	0.909	0.912	0.889	0.643	0.875	0.815
Logistic Regression	1.000	0.946	0.974	1.000	0.933	0.978	1.000	0.944	0.962	1.000	0.950	0.929	1.000	0.921	1.000	0.950	0.979	0.927	0.972	0.929	0.950	0.947	0.944	0.980	1.000	0.983	0.944	0.967	0.875	0.982
Multi Layer Perceptron	1.000	0.946	1.000	1.000	0.933	0.978	1.000	0.898	0.962	1.000	0.950	0.929	1.000	0.967	1.000	0.925	0.979	0.927	0.972	0.957	1.000	0.947	0.977	0.980	0.981	0.983	0.944	0.967	0.855	0.982
Nearest Neighbors	1.000	0.971	1.000	1.000	0.967	0.907	1.000	0.921	0.981	1.000	0.950	1.000	1.000	0.888	0.962	0.975	0.958	0.927	1.000	0.964	0.950	0.974	0.977	0.938	1.000	0.983	1.000	0.857	0.875	0.982
Quadratic Discriminant Analysis	1.000	0.971	0.974	1.000	0.967	1.000	1.000	0.944	0.962	0.971	0.975	1.000	1.000	0.911	1.000	0.975	0.979	0.927	0.972	0.964	0.981	0.974	0.977	0.980	1.000	0.983	0.964	0.967	0.875	0.982
Random Forest	1.000	0.971	1.000	1.000	1.000	0.978	1.000	0.921	0.962	1.000	0.975	1.000	1.000	0.933	1.000	0.975	0.979	0.927	0.972	1.000	0.950	0.947	0.977	0.958	1.000	0.983	0.944	0.967	0.938	0.982
SVM - linear	1.000	0.971	0.974	1.000	0.933	0.978	1.000	0.898	0.962	1.000	0.950	1.000	1.000	0.911	0.962	0.975	0.958	0.909	0.946	0.964	0.950	0.947	0.944	0.960	1.000	0.983	0.944	0.967	0.897	0.982
SVM - poly	1.000	0.971	0.974	1.000	0.933	1.000	1.000	0.921	0.962	1.000	0.975	1.000	0.938	0.944	1.000	0.975	0.958	0.927	0.946	0.929	0.950	0.947	0.944	0.938	1.000	0.983	0.944	0.950	0.875	0.667
SVM - rbf	0.500	0.500	0.500	1.000	0.967	0.978	1.000	0.944	0.962	1.000	0.950	0.500	1.000	0.911	0.962	0.925	0.958	0.500	0.500	0.964	0.500	0.974	0.500	0.980	0.818	0.500	0.500	0.643	0.855	0.500

Results exclude Nearest Centroid, Passive Aggressive Classifier and Perceptron.

Table 4.46 | AUROC score for 30 correlation image feature datasets for cell line U251

Classifiers	P1	P2	P3	P4	P5	P6	P7	P8	P9	P10	P11	P12	P13	P14	P15	P16	P17	P18	P19	P20	P21	P22	P23	P24	P25	P26	P27	P28	P29	P30
AdaBoost	0.608	0.816	0.759	0.500	0.830	0.607	0.756	0.811	0.839	0.812	0.803	0.752	0.778	0.820	0.700	0.703	0.801	0.744	0.759	0.820	0.700	0.700	0.653	0.750	0.572	0.840	0.909	0.521	0.710	0.871
Bernoulli Naive Bayes	0.528	0.657	0.754	0.500	0.562	0.500	0.838	0.608	0.858	0.565	0.665	0.840	0.676	0.797	0.665	0.574	0.763	0.556	0.548	0.592	0.681	0.620	0.655	0.672	0.689	0.736	0.500	0.450	0.753	0.669
Dummy Classifier	0.447	0.500	0.500	0.521	0.500	0.500	0.405	0.500	0.621	0.500	0.500	0.526	0.653	0.500	0.502	0.500	0.500	0.377	0.500	0.500	0.500	0.405	0.500	0.500	0.500	0.493	0.500	0.500	0.488	0.617
Extra Trees	0.500	0.500	0.611	0.500	0.567	0.500	0.784	0.721	0.871	0.651	0.500	0.500	0.500	0.558	0.591	0.576	0.538	0.500	0.500	0.500	0.500	0.753	0.533	0.500	0.500	0.500	0.500	0.500	0.500	0.500
Gaussian Naive Bayes	0.523	0.607	0.731	0.526	0.742	0.693	0.811	0.686	0.813	0.703	0.732	0.752	0.723	0.718	0.739	0.703	0.739	0.502	0.577	0.635	0.813	0.730	0.755	0.732	0.591	0.769	0.760	0.643	0.730	0.760
Gradient Boosting	0.733	0.841	0.917	0.734	0.842	0.921	0.782	0.855	0.904	0.837	0.803	0.929	0.778	0.865	0.878	0.866	0.798	0.891	0.788	0.856	0.794	0.835	0.730	0.897	0.864	0.786	0.871	0.769	0.710	0.815
**Helstrom Quantum Classifier**	0.525	0.657	0.836	0.677	0.721	0.728	0.864	0.776	0.755	0.871	0.846	0.950	0.802	0.855	0.857	0.791	0.753	0.891	0.784	0.899	0.863	0.756	0.844	0.710	0.825	0.912	0.871	0.643	0.733	0.909
Linear Discriminant Analysis	0.545	0.657	0.731	0.526	0.620	0.585	0.728	0.653	0.767	0.649	0.694	0.786	0.771	0.741	0.700	0.728	0.662	0.484	0.550	0.542	0.794	0.649	0.709	0.648	0.617	0.840	0.722	0.643	0.710	0.760
Logistic Regression	0.545	0.657	0.731	0.526	0.620	0.585	0.728	0.653	0.767	0.649	0.694	0.786	0.795	0.741	0.721	0.753	0.662	0.484	0.550	0.542	0.794	0.649	0.709	0.648	0.617	0.840	0.722	0.643	0.710	0.760
Multi Layer Perceptron	0.772	0.678	0.917	0.825	0.832	0.849	0.754	0.755	0.813	0.896	0.887	0.983	0.874	0.865	0.917	0.841	0.780	0.982	0.813	0.842	0.744	0.807	0.855	0.877	0.591	0.840	0.964	0.648	0.730	0.964
Nearest Neighbors	0.693	0.707	0.892	0.708	0.798	0.849	0.864	0.777	0.851	0.812	0.887	0.895	0.874	0.832	0.881	0.841	0.795	0.780	0.867	0.863	0.863	0.759	0.865	0.857	0.773	0.840	0.944	0.752	0.730	0.835
Quadratic Discriminant Analysis	0.483	0.599	0.782	0.526	0.709	0.728	0.784	0.698	0.767	0.753	0.732	0.857	0.795	0.774	0.760	0.703	0.756	0.593	0.604	0.679	0.826	0.703	0.755	0.752	0.636	0.752	0.742	0.610	0.730	0.760
Random Forest	0.710	0.791	0.889	0.682	0.921	0.893	0.892	0.832	0.787	0.837	0.916	0.857	0.881	0.888	0.958	0.862	0.840	0.909	0.709	0.877	0.863	0.728	0.798	0.835	0.734	0.857	0.982	0.714	0.813	0.815
SVM - linear	0.545	0.657	0.703	0.500	0.653	0.500	0.784	0.653	0.767	0.649	0.694	0.500	0.740	0.741	0.739	0.728	0.623	0.484	0.550	0.542	0.763	0.648	0.721	0.708	0.617	0.840	0.500	0.500	0.730	0.760
SVM - poly	0.690	0.741	0.917	0.696	0.809	0.764	0.728	0.721	0.813	0.841	0.857	0.895	0.778	0.753	0.857	0.891	0.724	0.909	0.788	0.957	0.763	0.810	0.821	0.938	0.545	0.769	0.927	0.719	0.752	0.853
SVM - rbf	0.545	0.632	0.731	0.500	0.720	0.500	0.811	0.500	0.813	0.649	0.500	0.752	0.716	0.500	0.780	0.500	0.500	0.520	0.500	0.585	0.794	0.673	0.732	0.667	0.572	0.752	0.500	0.571	0.730	0.760

Results exclude Nearest Centroid, Passive Aggressive Classifier and Perceptron.

Table 4.47 | AUROC score for 30 energy image feature datasets for cell line U251

Classifiers	P1	P2	P3	P4	P5	P6	P7	P8	P9	P10	P11	P12	P13	P14	P15	P16	P17	P18	P19	P20	P21	P22	P23	P24	P25	P26	P27	P28	P29	P30
AdaBoost	1.000	1.000	1.000	0.981	0.967	1.000	0.974	1.000	1.000	0.971	0.975	1.000	1.000	0.977	0.941	0.925	0.938	0.927	0.946	1.000	0.931	0.947	1.000	1.000	1.000	0.983	1.000	0.824	0.835	0.982
Bernoulli Naive Bayes	0.940	0.912	0.838	0.827	0.955	0.964	0.972	0.933	0.885	0.971	0.941	0.807	1.000	0.967	0.920	0.887	0.958	0.615	0.732	0.848	0.652	0.917	0.886	0.980	0.729	0.879	0.782	0.505	0.817	0.742
Dummy Classifier	0.508	0.419	0.500	0.463	0.518	0.500	0.349	0.562	0.500	0.594	0.500	0.500	0.589	0.505	0.500	0.599	0.519	0.540	0.466	0.500	0.533	0.500	0.473	0.447	0.500	0.500	0.613	0.400	0.500	0.500
Extra Trees	0.792	0.500	0.500	0.500	0.800	0.500	0.918	0.967	0.935	0.882	0.765	0.500	0.762	0.977	0.769	0.799	0.748	0.667	0.500	0.929	0.500	0.974	0.500	0.500	0.500	0.500	0.500	0.500	0.500	0.500
Gaussian Naive Bayes	0.917	1.000	0.947	1.000	1.000	1.000	0.944	1.000	1.000	1.000	0.941	1.000	1.000	0.977	0.962	0.950	0.938	0.927	0.946	0.978	0.931	0.972	1.000	0.980	1.000	0.912	0.964	0.895	0.918	0.927
Gradient Boosting	1.000	1.000	1.000	0.981	0.967	1.000	0.974	1.000	1.000	1.000	0.971	1.000	0.921	0.977	0.941	0.950	0.938	0.927	0.920	1.000	0.931	0.920	1.000	0.980	1.000	0.983	0.964	0.824	0.918	0.982
**Helstrom Quantum Classifier**	0.917	1.000	1.000	0.981	1.000	0.964	0.974	0.967	0.962	0.971	0.971	0.983	1.000	1.000	0.920	0.946	0.958	0.927	1.000	0.978	0.944	0.974	1.000	0.980	0.962	0.983	0.964	0.862	0.960	0.927
Linear Discriminant Analysis	0.960	1.000	0.921	1.000	1.000	0.929	0.972	0.933	0.923	0.971	0.941	0.983	0.976	0.967	0.917	0.887	0.920	0.927	0.762	0.891	0.844	0.944	0.909	0.958	1.000	0.967	0.964	0.769	0.920	0.982
Logistic Regression	1.000	1.000	0.974	0.981	1.000	0.929	1.000	0.967	0.942	0.971	0.941	1.000	1.000	1.000	0.941	0.946	0.958	0.927	0.946	0.957	0.931	0.918	1.000	0.958	0.981	0.983	0.964	0.824	0.940	0.982
Multi Layer Perceptron	1.000	1.000	1.000	0.981	1.000	0.929	0.974	0.967	0.942	0.971	0.971	1.000	1.000	0.977	0.938	0.916	0.958	0.927	0.946	0.957	0.931	0.921	1.000	0.958	1.000	0.983	0.964	0.857	0.918	0.982
Nearest Neighbors	1.000	1.000	1.000	1.000	1.000	0.964	0.972	0.967	0.981	1.000	0.946	1.000	1.000	1.000	0.920	0.946	0.920	0.944	0.974	1.000	0.963	1.000	0.977	0.958	1.000	0.983	0.964	0.824	0.980	0.982
Quadratic Discriminant Analysis	0.917	1.000	1.000	1.000	1.000	1.000	1.000	1.000	0.981	1.000	0.941	1.000	1.000	0.977	0.962	0.950	0.958	0.927	0.974	0.978	0.931	1.000	1.000	0.980	0.935	0.983	0.964	0.895	0.898	0.927
Random Forest	1.000	1.000	1.000	1.000	1.000	1.000	0.974	1.000	1.000	1.000	0.971	1.000	1.000	0.977	0.962	0.950	0.938	0.927	0.946	1.000	0.931	0.920	1.000	1.000	0.981	0.983	1.000	0.824	0.938	0.982
SVM - linear	1.000	1.000	1.000	1.000	1.000	0.964	1.000	0.967	0.962	1.000	0.971	1.000	1.000	1.000	0.962	0.946	0.958	0.927	0.946	0.957	0.931	0.974	1.000	0.958	1.000	0.983	0.964	0.895	0.898	0.982
SVM - poly	1.000	1.000	1.000	1.000	1.000	0.929	0.974	0.967	0.962	0.971	0.946	1.000	1.000	0.977	0.962	0.946	0.938	0.927	0.974	1.000	0.931	0.974	1.000	0.958	1.000	0.983	0.964	0.879	0.938	0.982
SVM - rbf	1.000	1.000	1.000	1.000	1.000	0.500	1.000	0.867	0.885	0.971	0.500	1.000	0.952	1.000	0.958	0.946	0.958	0.500	0.946	0.935	0.931	0.974	1.000	0.500	1.000	0.983	0.500	0.895	0.940	0.500

Results exclude Nearest Centroid, Passive Aggressive Classifier and Perceptron.

Table 4.48 | AUROC score for 30 homogeneity image feature datasets for cell line U251

Classifiers	P1	P2	P3	P4	P5	P6	P7	P8	P9	P10	P11	P12	P13	P14	P15	P16	P17	P18	P19	P20	P21	P22	P23	P24	P25	P26	P27	P28	P29	P30
AdaBoost	1.000	1.000	1.000	1.000	1.000	1.000	0.974	1.000	1.000	1.000	1.000	1.000	0.976	0.955	0.962	0.950	0.958	0.927	0.946	0.957	0.931	0.974	1.000	1.000	1.000	0.983	0.982	0.845	0.938	0.982
Bernoulli Naive Bayes	1.000	1.000	1.000	1.000	1.000	0.978	0.974	1.000	1.000	1.000	0.975	0.983	1.000	1.000	0.962	0.946	0.917	0.909	0.972	1.000	0.926	0.974	1.000	0.980	0.962	0.983	0.946	0.681	0.960	0.982
Dummy Classifier	0.428	0.500	0.500	0.500	0.495	0.533	0.542	0.550	0.500	0.485	0.500	0.500	0.542	0.338	0.571	0.500	0.500	0.669	0.572	0.500	0.470	0.500	0.500	0.610	0.500	0.500	0.522	0.471	0.530	0.500
Extra Trees	0.833	1.000	0.972	0.727	0.967	0.857	0.890	0.967	1.000	1.000	0.912	0.786	0.786	0.977	0.962	0.921	0.938	0.722	0.500	1.000	0.750	0.974	0.900	0.917	0.682	0.500	0.778	0.500	0.583	0.833
Gaussian Naive Bayes	1.000	1.000	1.000	1.000	0.967	0.978	0.974	1.000	1.000	1.000	0.946	1.000	1.000	0.977	0.962	0.950	0.958	0.927	0.946	1.000	0.950	0.974	1.000	0.980	1.000	0.983	0.982	0.895	0.938	0.982
Gradient Boosting	1.000	1.000	0.974	1.000	0.967	1.000	0.918	1.000	0.981	1.000	0.975	1.000	1.000	0.977	0.941	0.950	0.920	0.927	0.946	0.957	0.931	0.920	0.977	1.000	1.000	0.983	0.964	0.862	0.980	0.982
**Helstrom Quantum Classifier**	1.000	1.000	1.000	1.000	0.967	1.000	0.974	1.000	1.000	1.000	0.975	1.000	1.000	1.000	0.962	0.946	0.917	0.927	0.972	1.000	0.950	0.974	1.000	0.980	1.000	0.983	0.982	0.912	0.938	0.982
Linear Discriminant Analysis	1.000	1.000	1.000	1.000	0.967	1.000	0.974	1.000	1.000	1.000	0.975	1.000	1.000	1.000	0.962	0.946	0.917	0.927	0.972	1.000	0.931	0.974	1.000	1.000	1.000	0.983	0.982	0.840	0.918	0.982
Logistic Regression	1.000	1.000	0.974	1.000	0.967	1.000	0.974	1.000	0.981	1.000	1.000	1.000	1.000	1.000	0.962	0.975	0.938	0.927	0.946	0.978	0.950	0.974	1.000	1.000	1.000	0.983	0.982	0.840	0.918	0.982
Multi Layer Perceptron	1.000	1.000	1.000	1.000	0.967	1.000	1.000	0.967	0.981	1.000	1.000	1.000	1.000	0.977	0.962	0.946	0.878	0.927	0.946	0.978	0.931	0.974	1.000	1.000	1.000	0.983	0.982	0.840	0.897	0.982
Nearest Neighbors	1.000	1.000	1.000	1.000	0.967	0.978	0.974	1.000	1.000	1.000	0.975	1.000	1.000	1.000	0.962	0.950	0.896	0.927	0.946	1.000	0.950	0.974	1.000	0.980	1.000	0.983	0.982	0.912	1.000	0.982
Quadratic Discriminant Analysis	1.000	1.000	1.000	1.000	0.967	0.978	1.000	1.000	1.000	1.000	0.946	1.000	1.000	0.977	0.962	0.950	0.958	0.927	0.946	1.000	0.950	0.974	1.000	1.000	1.000	0.983	0.982	0.895	0.918	0.982
Random Forest	1.000	1.000	1.000	1.000	0.967	1.000	0.974	1.000	1.000	1.000	0.946	1.000	1.000	0.977	0.962	0.950	0.958	0.871	0.946	0.978	0.950	0.947	1.000	1.000	1.000	0.983	0.982	0.862	0.938	0.982
SVM - linear	1.000	1.000	1.000	1.000	0.967	1.000	0.974	1.000	1.000	1.000	1.000	1.000	1.000	1.000	0.962	0.946	0.917	0.927	0.972	1.000	0.950	0.974	1.000	1.000	1.000	0.983	0.982	0.840	0.958	0.982
SVM - poly	0.833	0.950	1.000	0.955	0.967	1.000	0.974	1.000	1.000	1.000	1.000	1.000	1.000	0.977	0.885	0.946	0.938	0.927	0.946	0.913	0.950	0.947	1.000	1.000	1.000	0.983	0.982	0.857	0.875	0.833
SVM - rbf	0.500	0.500	1.000	1.000	1.000	1.000	0.974	1.000	1.000	0.500	1.000	0.500	1.000	0.977	0.500	0.500	0.917	0.500	0.500	0.500	0.500	0.974	0.500	0.500	1.000	0.500	0.500	0.840	0.500	0.500

Results exclude Nearest Centroid, Passive Aggressive Classifier and Perceptron.

References

1. Haralick, R. M., Shanmugam, K. *et al.* Textural features for image classification. *IEEE Trans. Syst. Man Cybern.* **SMC-3**, 610–621, DOI: 10.1109/TSMC.1973.4309314 (1973).

2. Haralick, R. M. Statistical and structural approaches to texture. *Proc. IEEE* **67**, 786–804, DOI: 10.1109/PROC.1979.11328 (1979).

3. Rundo, L. *et al.* HaraliCU: GPU-powered Haralick feature extraction on medical images exploiting the full dynamics of gray-scale levels. In Malyshkin, V. (ed.) *Parallel Computing Technologies (PaCT)*, vol. 11657 of *LNCS*, 304–318, DOI: 978-3-030-25636-4_24 (Springer International Publishing, Cham, Switzerland, 2019).
